# Transcriptional profiling of a fungal granuloma reveals a low metabolic activity of *Paracoccidioides brasiliensis* yeasts and an actively regulated host immune response

**DOI:** 10.3389/fcimb.2023.1268959

**Published:** 2023-10-05

**Authors:** Bruno Montanari Borges, Rafael Berton Correia Ramos, Nycolas Willian Preite, Valéria de Lima Kaminski, Patrícia Alves de Castro, Maurício Camacho, Marina Ferreira Maximo, Taicia Pacheco Fill, Vera Lúcia Garcia Calich, Aimee M. Traynor, Özlem Sarikaya-Bayram, Sean Doyle, Özgür Bayram, Claudia Barbosa Ladeira de Campos, André Zelanis, Gustavo H. Goldman, Flávio Vieira Loures

**Affiliations:** ^1^ Institute of Science and Technology (ICT), Federal University of São Paulo (UNIFESP), São José dos Campos, SP, Brazil; ^2^ Faculty of Pharmaceutical Science of Ribeirão Preto (FCFRP), University of São Paulo (USP), Ribeirão Preto, SP, Brazil; ^3^ Institute of Chemistry, Universidade Estadual de Campinas, Campinas, SP, Brazil; ^4^ Department of Immunology, Institute of Biomedical Sciences, University of São Paulo (USP), São Paulo, Brazil; ^5^ Department of Biology, Maynooth University, Maynooth, County Kildare, Ireland

**Keywords:** paracoccidioidomycosis, granuloma, transcriptomic, proteomic, fungal infection

## Abstract

Granulomas are important immunological structures in the host defense against the fungus *Paracoccidioides brasiliensis*, the main etiologic agent of Paracoccidioidomycosis (PCM), a granulomatous systemic mycosis endemic in Latin America. We have performed transcriptional and proteomic studies of yeasts present in the pulmonary granulomas of PCM aiming to identify relevant genes and proteins that act under stressing conditions. C57BL/6 mice were infected with 1x10^6^ yeasts and after 8- and 12-weeks of infection, granulomatous lesions were obtained for extraction of fungal and murine RNAs and fungal proteins. Dual transcriptional profiling was done comparing lung cells and *P. brasiliensis* yeasts from granulomas with uninfected lung cells and the original yeast suspension used in the infection, respectively. Mouse transcripts indicated a lung malfunction, with low expression of genes related to muscle contraction and organization. In addition, an increased expression of transcripts related to the activity of neutrophils, eosinophils, macrophages, lymphocytes as well as an elevated expression of IL-1β, TNF-α, IFN-γ, IL-17 transcripts were observed. The increased expression of transcripts for CTLA-4, PD-1 and arginase-1, provided evidence of immune regulatory mechanisms within the granulomatous lesions. Also, our results indicate iron as a key element for the granuloma to function, where a high number of transcripts related to fungal siderophores for iron uptake was observed, a mechanism of fungal virulence not previously described in granulomas. Furthermore, transcriptomics and proteomics analyzes indicated a low fungal activity within the granuloma, as demonstrated by the decreased expression of genes and proteins related to energy metabolism and cell cycle.

## Introduction

Paracoccidioidomycosis (PCM) is a systemic mycosis characterized by granulomatous lesions caused by the thermically dimorphic fungus *Paracoccidioides brasiliensis*, *P. lutzii* and the recently described *P. restrepiensis, P. venezuelensis, and P. americana*. PCM is restricted to Latin America countries and has a high incidence in Brazil, Venezuela, and Colombia (Montenegro and Franco, 1994; Turissini et al., 2017). In Brazil, PCM ranges from 3,360 to 5,600 cases yearly, and represents the eighth cause of death among infectious and parasitic diseases. The infection occurs after the inhalation of mycelia fragments or conidia that remains as a localized benign lesion or evolve as an overt disease. The disease mainly affects rural works and can progress from a primary lesion or reactivation of a latent pulmonary focus (McEwen et al., 1987; Coutinho et al., 2015; de Oliveira et al., 2016; Martinez, 2017).

Granulomas are important immunological structures in the host defense against *P. brasiliensis* (Montenegro and Franco, 1994). The interaction with the host tissue initially triggers a congestive-exudative inflammatory reaction with a predominant influx of neutrophils. Progressively, these cells are replaced by macrophages, which are arranged in nodules and multinucleated giant cells, many of which contain fungi (Montenegro and Franco, 1994). With the evolution of the process, epithelioid cells are found and, concomitantly, lymphoplasmacytic halo forms. The presence of a central area of suppuration is a common finding as well as inflammatory exudate rich in lymphocytes, plasma cells, and eosinophils permeating or surrounding the granulomatous lesions. Fibrosis of varying intensity is usually found around the granuloma or areas of necrosis, which are gradually replaced by fibrous scar tissue (San-Blas, 1992; Egen et al., 2008).

Granuloma formation depends on the genetics of the host and the virulence factors of the fungus (San-Blas, 1992; Calich and Kashino, 1998; Bocca et al., 2013). Studies with granulomas from tuberculosis showed a reduced replication of *Mycobacterium tuberculosis*, and the induced expression of genes related to low pH, oxygen depletion, iron limitation, nitrosative stress, and nutrient starvation, revealing new stress-adaptive genes (Timm et al., 2003; Talaat et al., 2007; Garton et al., 2008). In the context of PCM, a change of environment associated with infection requires an adaptation of the fungal pathogen aiming to survive from the host immune response. This adaptation process involves changes in the pattern of gene expression, mainly of genes related to virulence and pathogenicity (San-Blas, 1992; Bocca et al., 2013). However, there is no information about which pathways are modulated in the host and in the fungus after the development of granulomatous pulmonary lesions. In contrast, previous *P. brasiliensis* transcriptomics and proteomics studies revealed potential virulence factors when yeasts were recovered from hepatic lesions of mice (Costa et al., 2007). In these studies, a high degree of virulence was correlated with an increase in the expression of genes involved in the glyoxylate cycle, nitrogen metabolism, and lipid biosynthesis (Costa et al., 2007). In another *in vivo* assay, after 6 hours of infection, yeasts had increased gene and protein expression for β-oxidation, glyoxylate cycle, pentose-phosphate pathway, and oxidative stress defense (Lacerda Pigosso et al., 2017).

Here, a transcriptomic approach was performed by using a dual-RNAseq assay from RNA extracted from pulmonary granulomatous lesions of mice 8- and 12-weeks post-infection with *P. brasiliensis*. We also carry out proteomic analysis of yeasts isolated from these lesions and from yeasts that were cultivated after recovery from lesions. Our data suggested an active and regulated host immune innate response, with elevated expression of genes that were not previously described in PCM, such as Ptx3, Lcn2, Clec4a2, when compared with healthy, uninfected lungs. Furthermore, our data indicated prominent Th1 and Th17 responses tightly regulated by checkpoint inhibition molecules. Additionally, there is an indication that iron is a major player in the chronic murine PCM granuloma, where the repression of the fungus energy metabolism occurs along with an increase in siderophore synthesis for iron uptake, revealing virulence mechanisms that have never been described in fungal granulomas.

## Materials and methods

### Ethics statement

All the experiments were performed in strict accordance with the Brazilian Federal Law 11,794 establishing procedures for the scientific use of animals, and the State Law establishing the Animal Protection Code of the State of São Paulo. All efforts were made to minimize animal suffering. The procedures were approved by the Ethics Committee on Animal Experiments of the Federal University of São Paulo (N° 9596270919).

### Animals

8 to 12 weeks old male C57BL/6 mice obtained from the Center for the Development of Experimental Models for Biology and Medicine (CEDEME-UNIFESP) and bred as specific pathogen-free mice in the animal facility of the Institute of Science and Technology at UNIFESP were used throughout this study.

### Paracoccidioides brasiliensis

The virulent strain Pb18 of the fungus *P. brasiliensis* was used and maintained by weekly sub-cultivation in a semi-solid medium of Fava Netto (Netto, 1955) at 37°C. The fungus was collected and washed with phosphate-buffered saline (PBS, pH 7.4). The number of yeasts was evaluated using a hemocytometer and their viability was determined using the Janus-Green vital dye. All procedures were performed with fungal suspension with viability greater than 95%.

### Infection of mice

**Figure 1:**
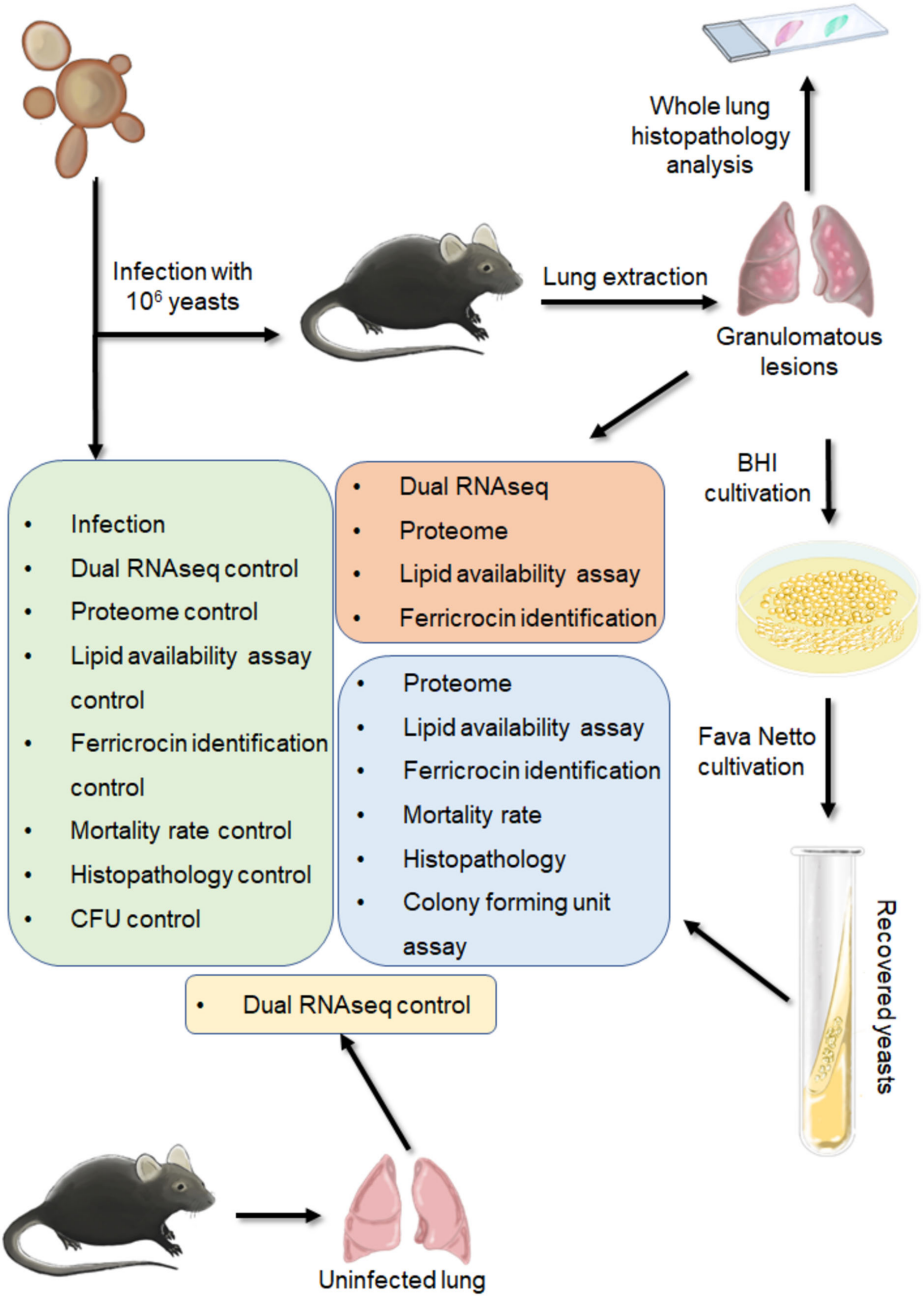
Schematic figure of the experiments.

Groups of C57BL/6 WT mice were anesthetized with intraperitoneal (ip.) injection of ketamine (90 mg/kg) and xylazine (10 mg/kg) and subjected to intratracheal (IT.) infection as previously described (Cano et al., 1995). Briefly, after anesthesia the animals were infected with 1 x 10^6^ yeasts of *P. brasiliensis* contained in 50 μL of PBS by surgical (IT.) inoculation, allowing the direct dispense of the yeasts into the lungs. After the infection the experiments were performed as showed in **Figure 1**.

### Histological analysis and colony-forming unit assays

To analyze the histopathology of the infected lungs, groups of 3-4 animals with two, six, eight and twelve weeks of infection were euthanized with xylazine (30 mg/kg) and ketamine (180 mg/kg) and the removed lungs were stored in 10% formaldehyde at 4 °C. Sections of 5 µm were stained with Hematoxylin-Eosin (H&E) for analysis of lesions and stained with Grocott for fungal evaluation. The anatomy of the lesion was analyzed according to size, morphology, and presence of fungi. The measurement of the area of the lesions was performed using the Leica DM750 microscope and the Leica Application Suite V4.8 software, thus allowing the observation of the establishment of the granuloma through time. To evaluate the fungal load a colony-forming unit (CFU) assay was performed. Briefly, the granulomatous lesions were separated from the apparently healthy regions with the help of a scalpel, and the tissue was macerated with a Doucer. Then, the cells were centrifuged at 1,200 x g for 10 minutes and the supernatant was discarded. The pellet was resuspended in 1 mL before cultivation of the yeasts in BHI. The colonies were counted daily.

### Granulomatous lesions extraction and yeast recovery

After eight and twelve weeks of infection, 3 independent groups with 2-4 animals were euthanized, the lungs were removed, and the granulomatous lesions were separated from the apparently healthy lung regions with the help of a scalpel. For the transcriptomics analysis the entire granulomas were frozen before the RNA extraction. For the proteomic analyzes of yeasts present in the granulomas, the lesions were enzymatically digested with collagenase (2 mg/mL) for 40 minutes at 37°C, as previously described (Lindell et al., 2006). The digested tissue was gently macerated with a Doucer and then centrifuged at 1,200 x g for 10 minutes. The supernatant was discarded, and the pellet resuspended in 2 mL of distilled water. Then 1 mL of this suspension was dispensed in two different Petri dishes, and the volume completed with 4 mL of distilled water. The dishes were incubated at 37 °C for 1 h to lyse the mouse cells. The suspension was then centrifuged (1,200 x g, 10 minutes) and the supernatant discarded, remaining only fungi cells in the tube. To evaluate the virulence and the proteins expressed by yeasts recovered from granulomas, these lesions were macerated with a Doucer, centrifuged (1,200 x g, 10 minutes) and then pellet resuspended in Phosphate-buffered saline (PBS). The recovered yeasts were cultivated in BHI for 5 days before cultivation in Fava Netto medium for 7 days (recovered yeast). The cultivation of the yeasts in BHI before cultivation in Fava Netto medium is necessary because there is growth factor in the BHI composition, which is essential for fungal growth after recovery from the lesions.

### Mortality rate

To evaluate the virulence of cultivated yeasts recovered from granulomas (experimental group) in comparison to the yeasts used in the original inoculation (control group), a group of six C57BL/6 WT mice IT. infected with the recovered fungus and another group of 6 mice was infected with the control fungus. Deaths were recorded daily. The Log-rank test (Mantel-Cox) was used for the survival curve.

### Total RNA extraction

Frozen granulomatous lesions were first macerated with the help of a pestle and pot. To proceed with total RNA extration, TRIzol LS (Thermo Scientific) was used according to the protocol described by Pisu et al., 2020. For the control lung was used uninfected healthy lungs, while for the control yeasts was used the original yeast suspension used in the infection. The control lung and control fungus were mechanical lysed with pestle and pot and zirconium beads, respectively, followed by RNA extraction with TRIzol LS (Thermo Scientific) according to the manufacturer’s protocol. After RNA extraction, the DNA was digested with Rnase-free Dnase (RQ1 Rnase-Free Dnase, Promega Corporation) in the presence of the ribonuclease inhibitor Rnasin® (Promega Corporation). The purification was done with the addition of 1 volume of phenol:chloroform (1:1), followed by 100% ethanol:3M sodium acetate (10:1) RNA precipitation. RNA integrity was verified with a 2100 Bioanalyzer (Agilent) and RNA 6000 Nano Kit (Agilent).

### cDNA preparation, sequencing, and alignment

First, rRNA was depleted using the RiboMinus™ Eukaryote Kit for RNA-Seq (Thermo Scientific). Then, cDNA library was synthesized using the NEBNext® Ultra II Directional RNA Library Prep Kit for Illumina® (New England BioLabs) and the NEBNext Multiplex oligos E7335 and E7500 (New England BioLabs). The cDNA library was sequenced in the NGS equipment Brazilian Biorenewables National Laboratory (LNBR), part of the Brazilian Centre for Research in Energy and Materials (CNPEM), a private non-profit organization under the supervision of the Brazilian Ministry for Science, Technology, and Innovations (MCTI). Because there is genetic material from two different organisms in the sample, these reads refer to two different genomes. Therefore, we chose to align against a chimeric genome of *P. brasiliensis* (Refseq accession number GCF_000150735.1) and *Mus musculus* (Refseq accession number GCF_000001635. 27). Bowtie2 was used in the default configuration for paired reads, but as it only returns the best pairing by default, the output mode was changed to report the best 5 alignments.

### Yeast protein extraction and trypsin digestion

Proteins from the yeasts were extracted using the Yeast Buster Protein Extraction Reagent (MerckMillipore) according to the manufacturer’s specifications. Benzonase Nuclease (MerckMillipore), protease inhibitors (Protease Inhibitor Cocktail, Sigma Aldrich) and phosphatase inhibitors (PhoSTOP, Sigma Aldrich) were also added to the buffer. In-solution trypsin digestion was performed according to Kleifeld et al., 2011 with modifications (Kleifeld et al., 2011). Briefly, a solution of 6 M guanidine hydrochloride (GuHCl) was added to a sample of 100 μg of protein from each cell lysate to a final concentration of 3 M GuHCl, followed by the addition of 5 mM dithiothreitol (DTT) (final concentration). The mixture was incubated at 37°C for 1 h. Iodoacetamide (IAA) was then added to a final concentration of 15 mM and the samples were incubated for 30 min at room temperature, in the dark. To quench the excess of IAA, DTT was added to a final concentration of 15 mM followed by the incubation of samples for 20 min at room temperature. Clean-up of samples was performed by the addition of ice-cold acetone (8 volumes) and methanol (1 volume), followed by the incubation of samples for 3 h at -80° C. After centrifugation at 14,000 x g for 10 min, protein pellets were washed twice with one volume of ice cold methanol and then resolubilized with NaOH solution (final concentration of 10 mM), followed by the addition of HEPES buffer (final concentration of 50 mM), pH 7.5, to a final volume of 100 μL. Trypsin (Proteomics grade; Sigma, USA) was added at 1:100 ratio (enzyme/substrate) and protein samples were incubated at 37°C for 18 h.

Tryptic peptides were desalted using C-18 cartridges SPE Extraction disks (3M EmporeTM), resuspended in 50 μL of 0.1% formic acid, quantified with Pierce™ Quantitative Colorimetric Peptide Assay (Thermo Scientific) and stored at -80° C.

### LC-MS/MS and protein identification

LC-MS/MS was made using the Thermo Fisher Q-Exactive mass spectrometer coupled to a Dionex RSLC nano. LC gradients ran from 4% to 35% B over 2 h, and data were collected using a Top15 method for MS/MS scans (O'Keeffe et al., 2014; Dolan et al., 2017). Mass spectrometric (RAW) data were analyzed with MaxQuant software (version 2.0.3.0). A False Discovery Rate (FDR) of 1% was required for both protein and peptide-to-spectrum match identifications. Raw data were searched against a target database restricted to the taxonomy ‘*Paracoccidioides brasiliensis’* (UniProt/Proteomes - UP000001628; 8,399 entries). This database was also combined with the sequences of 245 common contaminants and concatenated with the reversed versions of all sequences. Enzyme specificity was set to trypsin and up to two missed cleavages were allowed; cysteine carbamidomethylation was selected as fixed modification whereas methionine oxidation, glutamine/asparagine deamidation and protein N-terminal acetylation were selected as variable modifications. Peptide identification was based on a search with an initial mass deviation of the precursor ion of 4.5 ppm and the fragment mass tolerance was set to 20 ppm. Label-free quantitation was performed using the MaxLFQ algorithm (Cox and Mann, 2008; Cox et al., 2014) with the ‘re-quantify’ function of MaxQuant software enabled. As is observed from complex proteomes such as those of vertebrates, peptides can be shared between homologous proteins or splice variants, leading to “protein groups”. For each protein group in the MaxQuant’s ‘proteinGroups.txt’ file, the first protein entry was selected as representative.

### Differentially expressed genes and proteins analysis

Transcriptomic data were normalized by log2 and Z-score. For proteomics data, all the protein intensity values were log2-transformed and quantile-normalized using the ‘preprocessCore’ library in R scripting and statistical environment (Ihaka and Gentleman, 1996; Bolstad et al., 2003). Statistical analyses were performed using the ‘limma’ package in R/Bioconductor, using eBayes for granuloma data and treat for the recovered yeast (Gentleman et al., 2004; Smyth, 2004; Ritchie et al., 2015). Differentially expressed genes (DEG) and proteins analyzed by eBayes were delimited by an adjusted p-value < 0,05 and a cutoff of |fold change| > 1, where the p-values were adjusted for multiple testing with the Benjamini-Hochberg method. The data analyzed by treat don’t need a delimitation, as specified in the limma package user’s guide. Correlations between replicates were also calculated in R, using Pearson correlation coefficient. Genes and proteins that were differentially expressed in both infection groups in comparison to the control group were reanalyzed by pooling the expression values for 8 and 12 weeks, generating a new log (Fold change) and adjusted P-value for the disease for both experimental time points (disease).

### Functional analysis

For function prediction, Kyoto Encyclopedia of Genes and Genomes (KEGG) and Gene Ontology (GO) annotations were taken, as well as enrichment analyzes (Ashburner et al., 2000; Kanehisa and Goto, 2000; Kanehisa, 2019; Gene Ontology Consortium, 2021; Kanehisa et al., 2023). The *P. brasiliensis* Gene Onthology functions were taken from FungiDB. Gene Onthology enrichment was performed by Enrichr for *Mus musculus*, using default settings (Chen et al., 2013; Kuleshov et al., 2016; Xie et al., 2021). The *P. brasiliensis* metabolic pathway was obtained from KEGG.

### Staining of neutral lipids in yeast

Fungi obtained from 8 weeks granulomatous lesions, fungi recovered from granulomas and control fungi were stained with Oil Red O (Merck Millipore) using the manufacturer’s protocol with minor modifications. Briefly, a 0.5% solution of Oil Red O in isopropanol was diluted in water at a 3:2 ratio and incubated at room temperature (~ 25°C) for 1 hour. After that, the diluted solution was filtered using a 0.22 μm filter. The cells, previously fixed in 10% paraformaldehyde, were centrifuged and the supernatant discarded; then, the filtered Oil Red O solution was added in sufficient quantity to cover the fungi. Staining took place for 2 hours at room temperature. Subsequently, the cells were washed twice with ultrapure water and then resuspended in PBS for analysis on a microscope slide. The photos were taken using the Leica DM750 microscope and the Leica Application Suite V4.8 software, thus allowing the observation of stained neutral lipids inside the cells.

### Secondary metabolites extraction and analysis

Lung granuloma, control lung, control yeasts and recovered yeasts were frozen, and 100 mg of frozen material was macerated with the help of a pestle and pot. The secondary metabolites extraction was performed with 1 mL of methanol (100%; HPLC grade), followed by sonication bath for 40 minutes. For sample preparation, 500 L of each sample extract were filtered in a 0.22 μm PTFE and diluted to 1 mL volume with HPLC-grade methanol. LC-HRMS/MS analysis were performed in positive ionization mode in a Thermo Scientific QExactive Hybrid Quadrupole-Orbitrap Mass Spectrometer, with *m/z* range of 100–1500, capillary voltage at 3.5 kV, source temperature at 300°C and S-lens 50 V. The stationary phase was a Thermo Scientific Accucore C_18_ 2.6 μm (2.1 mm x 100 mm) column. Mobile phase composition was a gradient of formic acid (A) and acetonitrile (B). Eluent profile (A/B %): 95/5 for 5 minutes, up to 60/40 within 5 minutes, up to 55/45 within 2 minutes, up to 2/98 for 6 minutes, maintaning 2/98 for 2 minutes, down to 95/5 within 2 minutes, maintaning 95/5 for 2 minutes. Total run time was 24 minutes for each sample run and flow rate was 400 µL/min. Injection volume was 5 μL. MS spectra were acquired with *m/z* ranges from 100 to 1500, with 70000 mass resolution at 200 Da. MS^2^ spectra were acquired in data-dependent acquisition (DDA) mode. Normalized collision energy was applied stepwise (20, 30 and 40), and the 5 most intense precursors per cycle were fragmented with 17500 resolutions at 200 Da. Spectra data were processed with Xcalibur software (version 3.0.63) developed by Thermo Fisher Scientific. Compound Ferricrocin was annotated based on its exact mass and MS/MS fragmentation pattern.

### RT-qPCR

The total RNA extracted from granulomas was subjected to reverse transcriptase using ImProm-II (Promega) according to the manufacturer’s instructions. The synthesized cDNA was used for real-time PCR analysis using the SYBR green PCR master mix kit (Applied Biosystems) on the ABI 7500 Fast real-time PCR system (Applied Biosystems, Foster City, CA, USA). For the *M. musculus*, Usp9x was used as normalizer, while for the *P. brasiliensis*, pyruvate decarboxylase (PADG_00714) was employed. The genes and primers used for RNA-seq validation are listed in **Supplementary Table 1**.

### Statistical analysis

For statistical analyses, the mean ± standard deviation/standard error was compared. For comparison between two groups, an unpaired Student’s t-test was performed, while for multiple analyses, a one-way ANOVA was conducted with Bonferroni correction. The level of significance was set at p < 0.05. The Log-rank test (Mantel-Cox) was employed for survival curve analysis, for comparison between two groups.

## Results

### The granuloma is well-established after 8 weeks of infection

**Figure 2:**
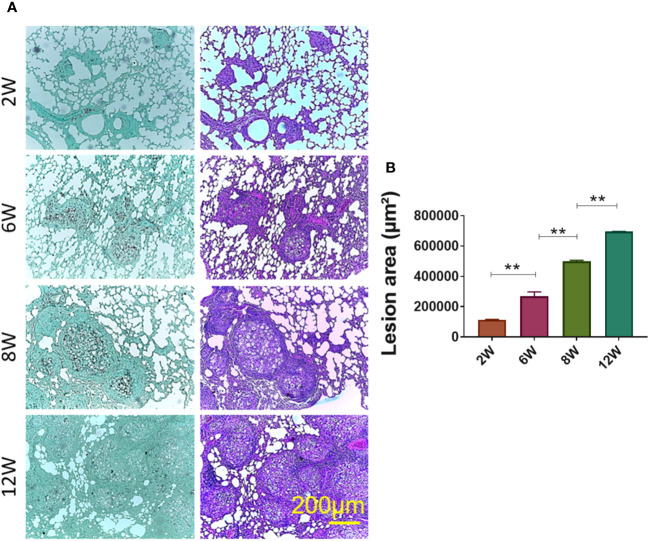
Establishment of granuloma. Groups of four C57BL/6 mice were infected by the IT. route with 1 × 10^6^ *P. brasiliensis* yeasts contained in 50 µL of PBS. After two (2W), six (6W), eight (8W) and twelve (12W) weeks of infection, the animals were euthanized and the lungs removed, which were stored in 10% formaldehyde at 4 °C. Sections of 5 µm were stained with Hematoxylin-Eosin (H&E; pink) for analysis of lesions and stained with Grocott (green) for fungal evaluation **(A)**. The anatomy of the lesion was analyzed according to size, morphology and presence of fungi. The image and the lesion area calculation were taken using the Leica DM750 microscope and the Leica Application Suite V4.8 software. Statistical analysis was performed using one-way ANOVA. Bars represent means ± standard error of lesion area (µm²) of groups of three mice **(B)** **p<0.01.

C57BL/6 mice were infected with 1x10^6^ *P. brasiliensis* yeasts via IT. route. Histological sections of two, six, eight and, twelve weeks of infection indicated that eight and twelve weeks of infection are the most adequate time points to obtain chronic granulomatous lesions considering the lesions organization and lung area occupied (**Figures 2A, B**).

To evaluate the fungal load a CFU assay was performed with the granulomatous lesions and the apparently healthy lung tissue. As it can be seen, approximately 90% of the yeasts present in the lung are within the granulomatous lesions, whereas 10% are present in the apparently healthy lung tissues (**Supplementary Figure 1**). Thus, all results from the granulomatous lesions comprise 90% of yeasts present in the lungs.

### Murine transcriptional profiling of pulmonary granulomatous lesions reveals new players in PCM host immune response

**Figure 3:**
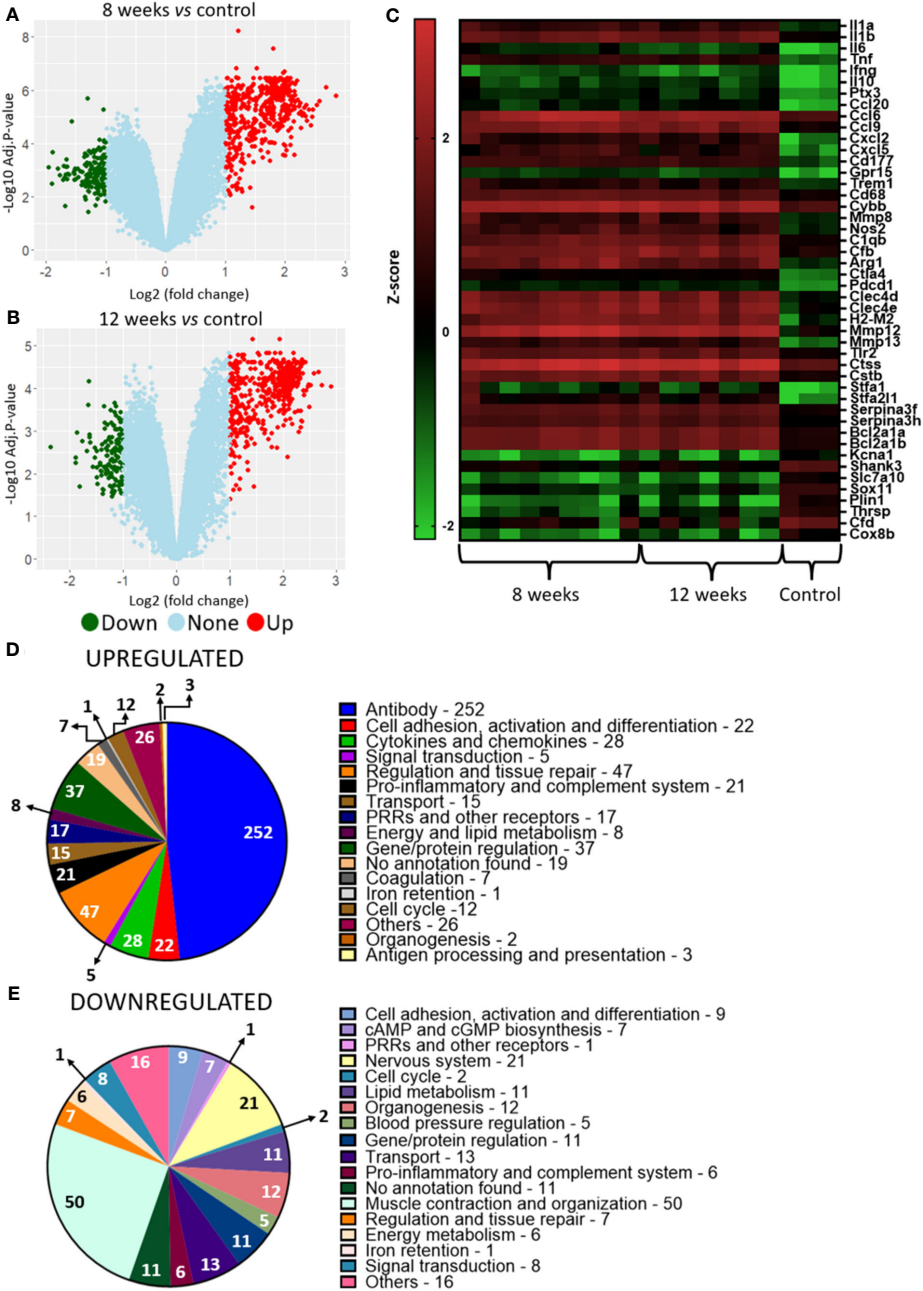
Murine transcriptional profiling of pulmonary granulomatous lesions reveals new players in PCM host immune response. Granulomatous lesions were extracted from two to four mice from three independent infections at eight weeks (8W) and twelve weeks (12W) after being infected with 1 x 10^6^ *P. brasiliensis* yeasts. Then, extraction of total RNA, removal of rRNA, and assembly of cDNA libraries were performed. The samples were sequenced and the fragments aligned with the reference genome for *M. musculus* using bowtie2. The FeatureCounts software was used to count the amount of each identified gene, which were normalized. Then, a differentially expressed genes analysis was made using *limma* and a functional analysis was carried out. To illustrate the differentially expressed genes, a volcano plot of the 8-weeks infection (8W) group in relation to the uninfected control **(A)** and a volcano plot of the 12-weeks infection group (12W) in relation to the uninfected control **(B)** were made. Additionally, a heatmap was created with selected genes **(C)**. The genes in red represent a higher expression and the green ones a lower expression for a given sample. The functions of the upregulated genes **(D)** and downregulated genes **(E)** were taken using GO, KEGG and Panther.

Analysis of hosts’ lung transcriptional profile comparing 8-weeks infected lungs to non-infected lungs show 563 transcripts upregulated and 131 with downregulated, while lungs from 12-weeks infection display 509 up- and 136 downregulated genes (**Figures 3A–C**). Functional analysis of the host’s transcripts whose abundance increased during *P. brasiliensis* infection using KEGG, GO and Panther databases identified transcripts encondig Pattern Recognition Receptors (PRRs) and other receptors that are related to antigen presentation, such as CD68, Ptx3 (Pentraxin-related protein PTX3), Clec4a2 (C-type lectin domain family 4 member A2), Clec4d (C-type lectin domain family 4 member D), Clec4n (C-type lectin domain family 4 member N), Clec7a (C-type lectin domain family 7 member A), TLR13 (toll-like receptor 13), TLR2 (toll-like receptor 2), and H2-M2 gene (Histocompatibility 2, M region locus 2) (**Figure 3D**; **Table 1**). Likewise, transcripts of some cytokines such as IL-1α, IL-1β, TNF and IFN-γ and chemokines (CXCL2, CXCL9, CXCL5, CCL8, CCL7, CCL9, CCL20, CXCL1, CC20, CXCL10, CCL6, CCL4 and CXCL13) were also increased in granulomatous host cells. The abundance of transcripts related to cell activation and chemotaxis functions (which are not chemokines) were also found in the disease, such as Cd177 and Trem1 (triggering receptor expressed on myeloid cells 1), Trem3 (triggering receptor expressed on myeloid cells 3) and Gpr15 (G protein-coupled receptor 15). Transcripts for pro-inflammatory proteins were also identified as more abundant in infected lungs, such as Nos2 (nitric-oxide synthase), Gbp2b (Guanylate-binding protein 2b), Cd300ld (CMRF35-like molecule 5), Cfb, C1qb, C3ar1, and C1qc. Transcripts for immune regulatory proteins, such as Arg1 (arginase-1), Il1rn (IL-1 receptor antagonist), CTLA4 (cytotoxic T-lymphocyte-associated protein 4) and, Pdcd1 (programmed cell death protein 1), as well as genes related to tissue repair (matrix metalloproteinase-12 (Mmp12) and collagenase 3 (Mmp13) were also found in higher levels when compared to transcripts in *naïve* lung control. Additionally, a high number of transcripts related to antibody chains were also identified as more expressed in the fungal granuloma. Taken together, these data point to a prominent and highly regulated Th1 response in addition to the production of antibodies by the host immune system.

**Table 1:** Differential expression of mice genes.

Acession number	Protein	Expression status	Log (Fold Change)	Adjusted p-value
UPs				
PRRs and other receptors				
Clec4n	C-type lectin domain family 6 member A	UP(D)	2,078333	3,78E-05
Clec7a	C-type lectin domain family 7 member A	UP(D)	2,020814	3,01E-05
Tlr2	toll-like receptor 2	UP(D)	1,960263	2,92E-05
Tlr13	toll-like receptor 13	UP(D)	1,903147	3,03E-05
Clec4d	C-type lectin domain family 4 member D	UP(D)	1,868473	0,000104
Ptx3	Pentraxin-related protein PTX3	UP(D)	1,851374	2,55E-05
Clec4e	C-type lectin domain family 4 member E	UP(D)	1,715215	5,88E-05
Cd68	CD68 antigen	UP(D)	1,338493	0,001332
Cell adhesion, activation and differentiation				
Gpr15	G protein-coupled receptor 15	UP(D)	2,086638	3,19E-05
Trem3	Triggering receptor expressed on myeloid cells 3	UP(D)	1,611529	8,33E-06
Trem1	triggering receptor expressed on myeloid cells 1	UP(D)	1,408704	0,002771
Cd177	CD177 antigen	UP(D)	1,202699	0,000194
Pro-inflammatory and complement system				
Nos2	nitric-oxide synthase, inducible [EC:1.14.13.39]	UP(D)	2,033614	3,01E-05
C1qa	Complement C1q subcomponent subunit A	UP(D)	2,025756	1,64E-05
Cfb	complement factor B [EC:3.4.21.47]	UP(D)	1,935486	2,77E-05
Cybb	NADPH oxidase 2 [EC:1.-.-.-]	UP(D)	1,775452	6,73E-05
C1qc	complement C1q subcomponent subunit C	UP(D)	1,729555	0,000174
C3ar1	C3a anaphylatoxin chemotactic receptor	UP(D)	1,615371	8,47E-05
C1qb	complement C1q subcomponent subunit B	UP(D)	1,346731	0,000138
Cd300ld	CMRF35-like molecule 5	UP(8)	1,049027	7,73E-07
Cytokines and chemokines				
Cxcl9	C-X-C motif chemokine 9	UP(D)	2,2741	1,45E-05
Ccl20	C-C motif chemokine 20	UP(D)	2,103374	4,42E-05
Cxcl1	C-X-C motif chemokine 1/2/3	UP(D)	2,073216	0,000421
Ccl7	C-C motif chemokine 7	UP(D)	2,057447	2,19E-05
Cxcl10	C-X-C motif chemokine 10	UP(D)	2,011544	8,14E-05
Cxcl13	C-X-C motif chemokine 13	UP(D)	1,95105	1,27E-05
Tnf	tumor necrosis factor superfamily, member 2	UP(D)	1,930536	1,23E-05
Il1b	interleukin 1 beta	UP(D)	1,895732	7,86E-05
Ccl8	C-C motif chemokine 8	UP(D)	1,886362	2,85E-05
Il1a	interleukin 1 alpha	UP(D)	1,850909	7,78E-06
Cxcl2	C-X-C motif chemokine 1/2/3	UP(D)	1,208573	0,000461
Ccl6	C-C motif chemokine 6	UP(D)	1,161484	1,39E-05
Cxcl5	C-X-C motif chemokine 5/6	UP(D)	1,114359	2,2E-05
Ifng	interferon gamma	UP(8)	1,105272	0,001062
Ccl4	C-C motif chemokine 4	UP(D)	1,103517	3,92E-05
Ccl9	C-C motif chemokine 9	UP(D)	1,070775	0,002911
Antigen processing and presentation				
H2-M2	Histocompatibility 2, M region locus 2	UP(D)	1,964557	3,04E-05
Antibody				
Ighv1-56	Immunoglobulin heavy variable 1-56	UP(D)	2,417073	2,54E-05
Igha	Immunoglobulin heavy constant alpha	UP(D)	2,233271	2,44E-05
Ighv1-22	Immunoglobulin heavy variable 1-22	UP(D)	2,007573	1,24E-05
Ighv1-21-1	Immunoglobulin heavy variable 1-21-1	UP(D)	2,002292	5,28E-05
Regulation and tissue repair				
Pdcd1	programmed cell death protein 1	UP(D)	2,073949	4,75E-05
Arg1	Arginase-1	UP(D)	2,059942	4,73E-05
Mmp12	matrix metalloproteinase-12 (macrophage elastase) [EC:3.4.24.65]	UP(D)	1,548912	8,8E-05
Mmp13	Collagenase 3	UP(D)	1,522556	2,12E-05
Ctla4	cytotoxic T-lymphocyte-associated protein 4	UP(D)	1,368578	0,000165
Il1rn	interleukin 1 receptor antagonist	UP(D)	1,244346	0,000409
Gene/protein regulation				
Serpina3f	Serine protease inhibitor A3F	UP(D)	2,06118	7,26E-05
Cstb	cystatin-A/B	UP(D)	1,830246	1,44E-05
Cstdc4	Cystatin domain-containing 4	UP(D)	1,791522	2,81E-05
Serpina3g	Serine protease inhibitor A3G	UP(D)	1,695611	0,001231
Ctss	cathepsin S [EC:3.4.22.27]	UP(D)	1,520351	3,51E-05
Stfa2l1	stefin A2 like 1	UP(D)	1,390768	0,000282
Stfa2	cystatin-A/B	UP(D)	1,205505	4,08E-05
Csta2	cystatin-A/B	UP(D)	1,088477	0,00027
Serpina3h	Serine protease inhibitor A3H	UP(D)	1,082125	0,000453
Ctsk	cathepsin K [EC:3.4.22.38]	UP(D)	1,064556	3,06E-05
Energy and lipid metabolism				
Gla	Growth factor independent protein 1	UP(D)	1,303367	0,001139
Pla1a	phosphatidylserine sn-1 acylhydrolase [EC:3.1.1.111]	UP(D)	1,285157	1,13E-05
Pla2g7	platelet-activating factor acetylhydrolase [EC:3.1.1.47]	UP(D)	2,167659	0,000343
Cell cycle				
Bcl2a1d	hematopoietic Bcl-2-related protein A1	UP(D)	1,946559	1,99E-05
Bcl2a1c	hematopoietic Bcl-2-related protein A1	UP(D)	1,878795	2,29E-05
Bcl2a1a	hematopoietic Bcl-2-related protein A1	UP(D)	1,806497	1,6E-05
Bcl2a1b	hematopoietic Bcl-2-related protein A1	UP(D)	1,722721	0,0003
Iron retention				
Lcn2	lipocalin 2	UP(D)	1,294766	0,000263
DOWNs				
Muscle contraction and organization				
Tnnc1	troponin C, slow skeletal and cardiac muscles	DOWN(D)	-1,77016	0,000138
Myh4	Myosin-4	DOWN(8)	-1,7189	0,002297
Myh13	myosin heavy chain 1/2/3/4/8/13/7B/15	DOWN(8)	-1,61232	0,00143
Tnnt2	troponin T, cardiac muscle	DOWN(D)	-1,45826	0,011979
Tnni3	troponin I, cardiac muscle	DOWN(D)	-1,22069	0,003786
Myh3	Myosin-3	DOWN(8)	-1,18001	0,007459
Myh6	myosin heavy chain 6/7	DOWN(D)	-1,17542	0,000529
Myl1	Myosin light chain 1/3, skeletal muscle isoform	DOWN(8)	-1,12704	0,006081
Nervous system				
Kcna1	potassium voltage-gated channel Shaker-related subfamily A member 1	DOWN(D)	-1,43254	0,005246
Slc17a7	MFS transporter, ACS family, solute carrier family 17 (sodium-dependent inorganic phosphate cotransporter), member 6/7/8	DOWN(8)	-1,32168	0,000511
Kcnh2	potassium voltage-gated channel Eag-related subfamily H member 2	DOWN(D)	-1,3173	0,013463
Shank3	SH3 and multiple ankyrin repeat domains protein	DOWN(D)	-1,25195	0,005262
Slc7a10	solute carrier family 7 (D/L-type amino acid transporter), member 10	DOWN(D)	-1,13547	0,00345
Organogenesis				
Bmp6	bone morphogenetic protein 6	DOWN(D)	-1,4241	0,008604
Sox11	transcription factor SOX11/12 (SOX group C)	DOWN(D)	-1,13558	0,002878
Lipid metabolism				
Cyp1a1	cytochrome P450 family 1 subfamily A1 [EC:1.14.14.1]	DOWN(D)	-1,23017	0,001407
Thrsp	thyroid hormone responsive	DOWN(D)	-1,14802	0,001858
Plin1	perilipin-1	DOWN(D)	-1,13331	0,00144
Energy metabolism				
Cox8b	cytochrome c oxidase subunit 8	DOWN(D)	-1,3867	0,004553
Cox7a1	cytochrome c oxidase subunit 7a	DOWN(12)	-1,12561	0,008107
Pro-inflammatory				
Cfd	complement factor D [EC:3.4.21.46]	DOWN(D)	-1,04996	0,001562
Iron retention				
Hamp	hepcidin	DOWN(D)	-1,50399	0,002563

Granulomatous lesions were extracted from two to four mice from three independent infections at eight weeks and twelve weeks after being infected with 1x10^6^ *P. brasiliensis* yeasts. Then, extraction of total RNA, removal of rRNA, and assembly of cDNA libraries were performed. The samples were sequenced and the fragments aligned with the reference genome for M. musculus using bowtie2. The FeatureCounts software was used to count the amount of each identified gene, which were normalized. Then, a differentially expressed gene was made using the *limma* and a functional analysis was carried out using GO, KEGG and Panther. The Table shows the differentially expressed genes with the values of Log (Fold change) and adjusted p-value. The expression status column represents the upregulated or downregulated genes for the eight-week infection group (8), the twelve-week infection group (12), and the genes that were differentially expressed in both infection groups were combined and reanalyzed, resulting in an expression status designation for the disease (D).

Transcripts to IL-17 and IL-21 were found exclusively in granulomas from infected animals, as well as some transcripts related to PRRs (the C-type lectin domain family 6 member A and theCD209 antigen-like protein E), several variable portions of T cell receptors, myeloperoxidase and genes related to the regulation of the immune response and tissue repair (Matrix metalloproteinase-1, Matrix metalloproteinase-7) (**Supplementary Figure 2**), indicating a Th17 response in the granuloma. Together with the enrichment analysis where a Th17-related response was present in higher abundance in the lung granuloma when compared to uninfected lungs, the transcripts profile exclusively found in our analysis (**Supplementary Table 5**) support that the susceptibility of the mice linage used in this work might be a Th17 response. Not only transcripts for immune response proteins were upregulated within the granuloma, but also transcripts for enzymes related to lipid metabolism such as Pla2g7 (platelet-activating factor acetylhydrolase), Gla (alpha-galactosidase) and Pla1a (phosphatidylserine sn-1 acylhydrolase), as well as genes related to protein regulation (Ctss, Ctsk, Stlfa2l1, stfa2, stfa1, Cstdc6, Csta2, Cstb, Serpina3g, Serpina3h and Serpina3f) and cell cycle/cell death regulation (Bcl2a1b, Bcl2a1c, Bcl2a1d and Bcl2a1a).

In contrast, genes involved in muscle organization and contraction, such as myosin and troponin, nervous system, action potential and organogenesis (Shank3, Slc7a10, Slc17a7, Kcna1, Kcnh2 and Sox11) were down-regulated (**Figure 3E**; **Table 1**). The repression of these genes may be related to impaired lung function within the granuloma since immune cells are expected to be located there, and as seen from **Figure 2A** there are less normal lung tissue present. Interestingly, lipid metabolism was also identified among repressed genes, as for example, [Cyp1a1 (cytochrome P450 family 1 subfamily A1), Plin1 (perilipin-1), Thrsp (thyroid hormone responsive)]. Curiously, Hepcidin (Hamp), a gene related to the negative regulation of iron availability was also downregulated in the granulomatous lesions.

### Transcriptional profiling of granulomatous *Paracoccidioides brasiliensis* yeasts shows transcripts associated with siderophores biosynthesis and downregulation of metabolism and cell cycle associated genes

**Figure 4:**
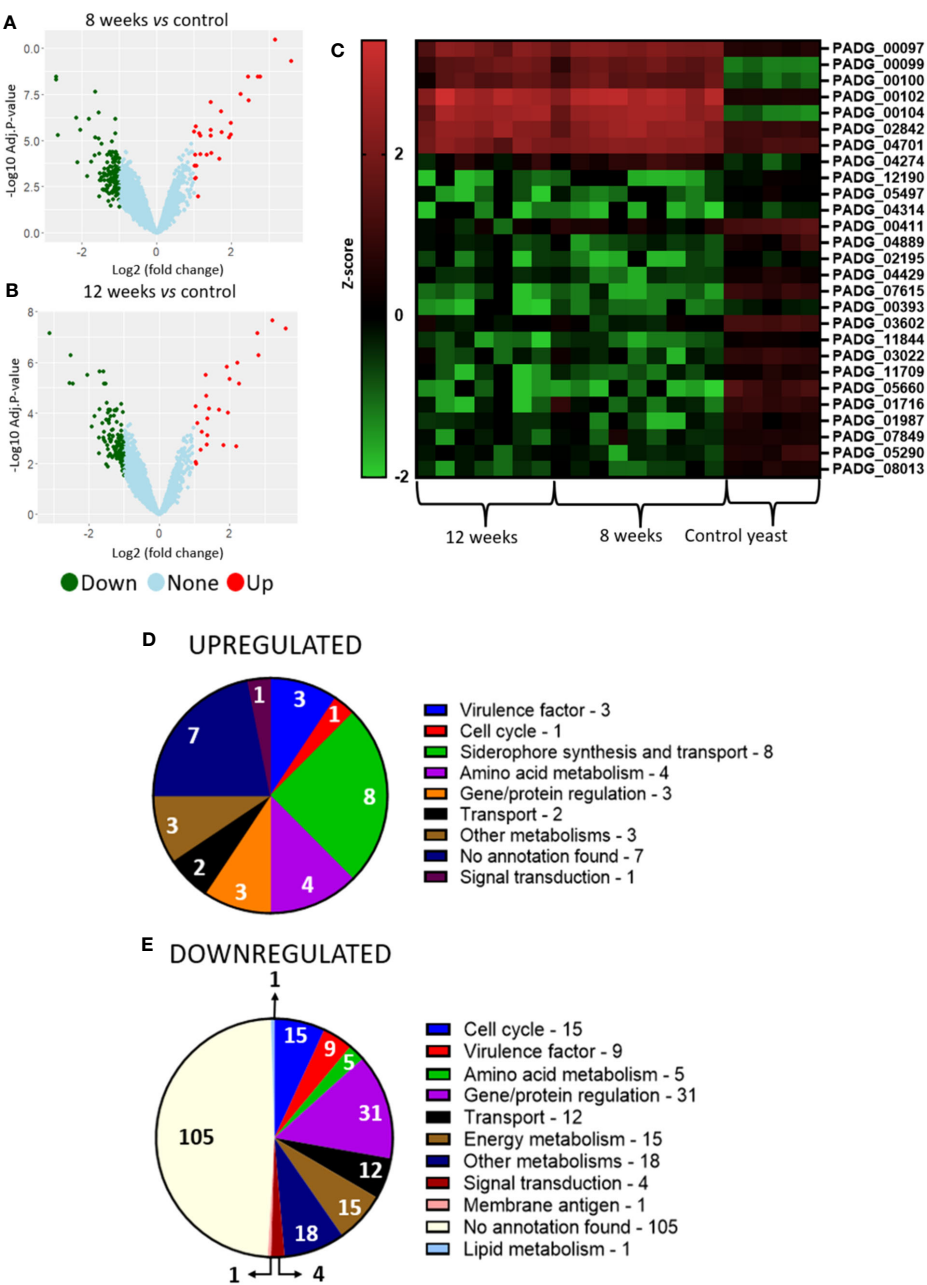
Transcriptome of granulomatous yeasts indicates upregulation of siderophores biosynthesis and downregulation of metabolism and cell cycle. Granulomatous lesions were extracted from two to four mice from three independent infections at eight weeks (8W) and twelve weeks (12W) after being infected with 1 x 10^6^ P*. brasiliensis* yeasts. Then, extraction of total RNA, removal of rRNA, and assembly of cDNA libraries were performed. The samples were sequenced and the fragments aligned with the reference genome for *P. brasiliensis* using bowtie2. The FeatureCounts software was used to count the amount of each identified gene, which were normalized. Then, a differentially expressed genes analysis was made using *limma* and a functional analysis was carried out. To illustrate the differentially expressed genes, a volcano plot of the 8-weeks infection (8W) group in relation to the control yeasts **(A)** and a volcano plot of the 12-weeks infection group (12W) in relation to the control yeasts **(B)** were made. Additionally, a heatmap was created with selected genes **(C)**. The genes in red represent a higher expression and the green ones a lower expression for a given sample. The functions of the upregulated genes **(D)** and downregulated genes **(E)** were taken using GO, KEGG and FungiDB.

Analysis of granuloma yeasts DEGs comparing 8- and 12-weeks infected lungs to control yeasts show 37 up- and 139 downregulated genes and 28 up- and 157 downregulated genes, respectively (**Figures 4A–C**). This large number of repressed genes when compared to induced genes may indicate a possible fungal metabolism repression within the granuloma. Functional analysis of the corresponding gene products was also carried out by using the KEGG, GO and FungiDB databases. A large number of the transcripts augmented in the fungus within the granuloma are related to the synthesis and transport of siderophores, such as: sidA (L-ornithine-N5-oxygenase; PADG_00097), sidI (Acyl-CoA ligase; PADG_00099), sidF (Hydroxyornithine transacylase; PADG_00100), fusarinin C synthase (sidD; PADG_00102), and OXR1 oxidoreductase (PADG_00104) (**Figure 4D**; **Table 2**). Siderophores are molecules responsible for the capture and storage of iron, being considered an important virulence factor in several pathogens (Schrettl et al., 2007; Blatzer et al., 2011; Silva et al., 2020).

**Table 2:** Differential expression of yeasts genes.

Acession number	Protein	Expression status	Log (Fold Change)	Adjusted p-value
UPs				
Siderophore synthesis and transport				
PADG_00097	L-ornithine N5-oxygenase SidA	UP(D)	3,594664	2,32E-08
PADG_00095	MFS siderochrome iron transporter 1	UP(D)	3,188131	1,12E-08
PADG_00100	Hydroxyornithine transacylase SIDF	UP(D)	2,354155	3,4E-06
PADG_00102	fusarinine C synthase	UP(D)	2,227774	5,26E-07
PADG_00104	Oxidoreductase OXR1	UP(D)	1,957033	4,87E-05
PADG_00099	Acyl-CoA ligase SID4	UP(D)	1,406269	8,21E-05
Virulence factors				
PADG_02842	Superoxide dismutase, Cu-Zn	UP(D)	1,424825	3,48E-05
PADG_04701	alcohol dehydrogenase [EC:1.1.1.-]	UP(8)	1,042112	1,75E-06
PADG_04274	polysaccharide synthase Cps1	UP(8)	1,027865	0,001103
Amino acid metabolis				
PADG_07440	Amino acid permease	UP(D)	1,812242	8,64E-07
PADG_08406	O-acetylhomoserine/O-acetylserine sulfhydrylase [EC:2.5.1.49 2.5.1.47]	UP(D)	1,637297	0,000873
PADG_00215	aromatic-L-amino-acid/L-tryptophan decarboxylase [EC:4.1.1.28 4.1.1.105]	UP(D)	1,514618	0,000419
PADG_02214	4-aminobutyrate aminotransferase/(S)-3-amino-2-methylpropionate transaminase [EC:2.6.1.19 2.6.1.22]	UP(8)	1,140801	4,12E-06
Other metabolisms				
PADG_06196	Flavin oxidoreductase hxnT	UP(D)	1,98727	4,73E-06
PADG_03085	Mannose-P-dolichol utilization defect 1 protein homolog	UP(12)	1,076101	0,000244
PADG_05433	pyridoxamine 5’-phosphate oxidase [EC:1.4.3.5]	UP(8)	1,032033	5,7E-05
DOWNs				
Gene/protein regulation				
PADG_04314	Zn(2)-C6 fungal-type domain-containing protein	DOWN(8)	-1,24281	4,13E-05
PADG_04889	Zn(2)-C6 fungal-type domain-containing protein	DOWN(D)	-1,23166	0,002813
PADG_05497	GATA-binding protein, other eukaryote	DOWN(D)	-1,22538	0,002127
PADG_02195	DNA repair protein Swi5/Sae3	DOWN(8)	-1,21794	0,00134
PADG_04429	Structure-specific endonuclease subunit SLX4	DOWN(8)	-1,14817	0,00099
PADG_00411	C6 transcription factor (Ctf1B)	DOWN(D)	-1,05195	0,00555
PADG_06413	C6 finger domain transcription factor nscR	DOWN(8)	-1,00975	0,008026
Energy metabolism				
PADG_11844	aconitate hydratase	DOWN(D)	-1,52072	0,01042
PADG_00393	hexokinase [EC:2.7.1.1]	DOWN(12)	-1,38879	0,000134
PADG_03602	acyl-CoA dehydrogenase	DOWN(D)	-1,32085	0,000697
PADG_05290	Cytochrome b5 heme-binding domain-containing protein	DOWN(D)	-1,07453	0,005483
Cell cylce				
PADG_03022	PLK/PLK1 protein kinase	DOWN(D)	-1,25446	0,002207
PADG_11709	serine/threonine-protein kinase TTK/MPS1 [EC:2.7.12.1]	DOWN(D)	-1,23479	5,54E-05
Virulence factors				
PADG_01954	superoxide dismutase, Fe-Mn family [EC:1.15.1.1]	DOWN(D)	-2,55152	5,85E-06
PADG_05660	Fungal nitric oxide reductase	DOWN(D)	-1,38287	0,007157
PADG_01716	alcohol dehydrogenase zinc-binding	DOWN(D)	-1,35725	0,000574
Other metabolisms				
PADG_02384	porphobilinogen synthase [EC:4.2.1.24]	DOWN(D)	-1,38467	0,000981
PADG_07849	N5-hydroxyornithine acetyltransferase [EC:2.3.1.-]	DOWN(12)	-1,12162	0,005081
PADG_04432	alpha-amylase [EC:3.2.1.1]	DOWN(D)	-1,06127	0,002921

Granulomatous lesions were extracted from two to four mice from three independent infections at eight weeks and twelve weeks after being infected with 1x10^6^ *P. brasiliensis* yeasts. Then, extraction of total RNA, removal of rRNA, and assembly of cDNA libraries were performed. The samples were sequenced and the fragments aligned with the reference genome for *P. brasiliensis* using bowtie2. The FeatureCounts software was used to count the amount of each identified gene, which were normalized. Then, a differentially expressed gene was made using the *limma* and a functional analysis was carried out using GO, KEGG and FungiDB. The Table shows the differentially expressed genes with the values of Log (Fold change) and adjusted p-value. The expression status column represents the upregulated or downregulated genes for the eight-week infection group (8), the twelve-week infection group (12), and the genes that were differentially expressed in both infection groups were combined and reanalyzed, resulting in an expression status designation for the disease (D).

In addition, genes that have already been described as virulence factors such as superoxide dismutase (PADG_02842), alcohol dehydrogenase (PADG_04701) (two non-iron enzymes that also manage antioxidant defenses) and polysaccharide synthase Cps1 (PADG_04274) were also found as upregulated. Superoxide dismutase was identified as upregulated in both 8- and 12-weeks infection groups. Transcripts which products are related to amino acid metabolism such as amino acid permease (PADG_07440), O-acetylhomoserine/O-acetylserine sulfhydrylase (PADG_08406) and aromatic-L-amino-acid/L-tryptophan decarboxylase (PADG_00215) were induced in both infection groups, while 4-aminobutyrate aminotransferase/(S)-3-amino-2-methylpropionate transaminase (PADG_02214) was induced only in the 8-weeks infection group.

Transcripts for gene and protein expression/degradation regulators are the most downregulated in the transcriptome of the fungus within the granuloma, in addition to transcripts related to the control of cell cycle, energy metabolism and virulence factors (**Figure 4E**; **Table 2**). Among the repressed genes, we identified the transcription factors (TFs) C6 finger domain TF nscR (PADG_06413), GATA-binding protein (PADG_05497), Zn (2)-C6 fungal-type domain-containing protein (PADG_04314), C6 TF (Ctf1B) (PADG_00411) and Zn (2)-C6 fungal-type domain-containing protein (PADG_04889). In addition to the repressed TFs, there are also two genes related to DNA repair that are downregulated, the DNA repair protein Swi5/Sae3 (PADG_02195) and the structure-specific endonuclease subunit SLX4 (PADG_04429). Furthermore, transcripts which products are related to energy metabolism were also repressed in the granulomatous yeasts, such as glucan 1,3-beta-glucosidase (PADG_07615), hexokinase (PADG_00393), acyl-CoA dehydrogenase (PADG_03602) and aconitate hydratase (PADG_11844). We also identified the downregulation of genes related to oxidative phosphorylation, such as cytochrome b5 (PADG_05290) and NADH dehydrogenase (ubiquinone) 1 alpha subcomplex subunit 2 (PADG_04699). Furthermore, some genes related to fungal virulence were also found at lower expression levels when compared to the control fungus, such as fungal nitric oxide reductase (PADG_05660), alcohol dehydrogenase (PADG_01716 and PADG_01987), superoxide dismutase (PADG_01954) and sidL (N5- hydroxyornithine acetyltransferase (PADG_07849).

### Proteome of the *Paracoccidioides brasiliensis* present in the granuloma indicate low protein production, low protein turnover and reprograming of the primary metabolism

**Figure 5:**
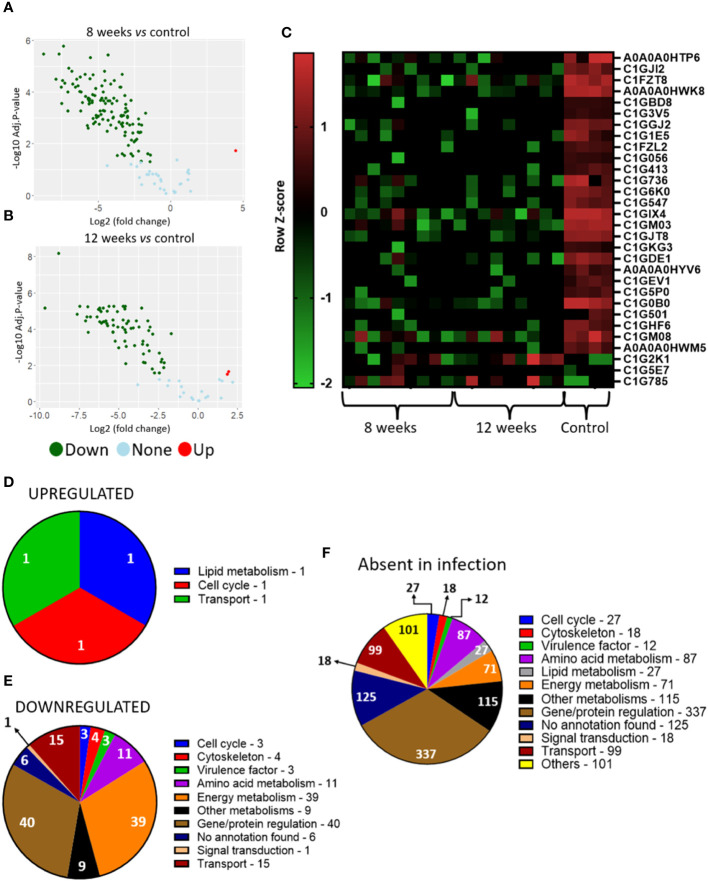
*P. brasiliensis* proteins present in the granuloma indicate low protein production and a metabolic repression. Granulomatous lesions were extracted from two to four mice from three independent infections at eight weeks (8W) and twelve weeks (12W) after being infected with 1 x 10^6^ *P. brasiliensis* yeasts. After lysing the animal cells, the fungal proteins were extracted and digested with trypsin and resolubilized in 0.1% formic acid. Peptides were analyzed by LC-MS/MS and proteins identified by MaxQuant software. Proteins containing more than two unique peptides had their intensity normalized by log2 followed by quantile normalization within each experimental repeat. Then, a differentially abundant proteins analysis was made using *limma* and a functional analysis was carried out. To illustrate the differentially abundant proteins, a volcano plot of the 8-weeks infection (8W) group in relation to the control yeasts **(A)** and a volcano plot of the 12-weeks infection group (12W) in relation to the control yeasts **(B)** were made. Additionally, a heatmap was created with selected proteins **(C)**. The proteins in red represent a higher abundance and the green ones a lower abundance for a given sample. The functions of the upregulated proteins **(D)**, downregulated proteins **(E)** and the proteins absent in the infection **(F)** were taken using GO, KEGG and FungiDB.

We identified 157 and 97 proteins after 8- and 12-weeks of infection, respectively, and 1199 in the control group. When comparing the yeasts present in the granulomatous lesions at 8-weeks of infection with the control group, 1 upregulated protein and 124 downregulated proteins were identified, while the comparison of the 12-weeks infection group with the control group showed a total of 2 more abundant and 74 less abundant proteins (**Figures 5A–C**).

Increased abundance was observed in only three proteins, glycerol-3-phosphate dehydrogenase (C1G2K1), which is related to lipid metabolism; replication factor C subunit 3 (C1G5E7), involved in DNA replication, DNA repair and regulation of cell cycle progression in yeast; and GTP-binding protein ypt5 (C1G785), involved in vesicles transport (**Figure 5D**; **Table 3**). The six different proteins related to siderophore biosynthesis and transport (**Table 2**) which transcripts have accumulated in granulomatous yeasts, have not been identified among the proteins revealed by the proteome, nor any other iron-capture protein have been identified among the accumulated proteins.

**Table 3:** Differential abundance of yeasts proteins.

Acession number	Protein	Expression status	Log (Fold Change)	Adjusted p-value
UPs				
Lipid metabolism				
C1G2K1	Glycerol-3-phosphate dehydrogenase	UP(12)	1,924377	0,02212
Cell cycle				
C1G5E7	Replication factor C subunit 3	UP(8)	4,474333	0,018531
Transport				
C1G785	GTP-binding protein ypt5	UP(12)	1,864712	0,030992
DOWNs				
Gene/protein regulation				
C1G0E5	40S ribosomal protein S14	DOWN(8)	-5,76095	0,001957
C1G5P0	pre-mRNA-splicing helicase BRR2 [EC:3.6.4.13]	DOWN(D)	-4,64777	2,39E-05
C1G0P0	molecular chaperone DnaK	DOWN(12)	-2,42964	0,026103
Energy metabolism				
C1G294	succinyl-CoA synthetase alpha subunit [EC:6.2.1.4 6.2.1.5]	DOWN(D)	-3,29572	0,003605
C1G2P3	enoyl-CoA hydratase [EC:4.2.1.17]	DOWN(D)	-4,37849	2,82E-05
C1G2W2	Pyruvate kinase (EC 2.7.1.40)	DOWN(D)	-5,32367	0,00092
C1GM03	Cytochrome b-c1 complex subunit 2	DOWN(D)	-4,10832	0,000135
Transport				
C1FZ88	importin subunit alpha-6/7	DOWN(8)	-4,36192	0,003272
C1GAF5	Coatomer subunit alpha	DOWN(8)	-2,60008	0,001756
C1GJS2	Phosphatidylinositol transfer protein SFH5 (PITP SFH5)	DOWN(D)	-7,21769	1,52E-05
Amino acid metabolism				
C1G3V5	Aspartate aminotransferase (EC 2.6.1.1)	DOWN(12)	-5,05193	7,6E-05
C1GBD8	3-oxoacid CoA-transferase [EC:2.8.3.5]	DOWN(8)	-6,39235	5,76E-05
C1GBT4	1-pyrroline-5-carboxylate dehydrogenase [EC:1.2.1.88]	DOWN(8)	-6,07164	0,00025
Virulence factor				
C1GKT9	Thioredoxin domain-containing protein	DOWN(8)	-6,48413	3,21E-05
C1G7K8	cytochrome c peroxidase [EC:1.11.1.5]	DOWN(D)	-3,10035	0,000272
C1GJI2	Superoxide dismutase [Cu-Zn]	DOWN(D)	-3,5651	0,00071
Other metabolisms				
C1GG77	Carboxypeptidase Y homolog A (EC 3.4.16.5)	DOWN(D)	-2,92665	0,00822
C1GJM4	Vacuolar aminopeptidase I	DOWN(D)	-4,32296	0,000825

Granulomatous lesions were extracted from two to four mice from three independent infections at eight weeks and twelve weeks after being infected with 1x10^6^ *P. brasiliensis* yeasts. After lysing the animal cells, the fungal proteins were extracted and digested with trypsin and resolubilized in 0.1% formic acid. Peptides were analyzed by LC-MS/MS and proteins identified by MaxQuant software. Proteins containing more than two unique peptides had their intensity normalized by log2, then quantile normalized within each experimental repeat. The *limma* package was used for differentially abundant proteins analysis and GO, KEGG and FungiDB were used for functional analysis. The Table shows the differentially abundant proteins with the values of Log (Fold change) and adjusted p-value. The expression status column represents the upregulated or downregulated proteins for the eight-week infection group (8), the twelve-week infection group (12), and the proteins that were differentially expressed in both infection groups were combined and reanalyzed, resulting in an expression status designation for the disease (D).

Instead, yeast cells sharply down regulate two key iron-dependent enzymes required for energy production by mitochondria and for antioxidant defence, respectively, the Cytochrome b-c1 complex subunit 2 (C1GM03), decreased by 4,11-fold; and cytochrome c peroxidase [EC:1.11.1.5], decreased by 3,10035 fold; that will be further discussed in this work.

Along with these iron-dependent enzymes, a large number of reduced abundance proteins were found (**Figure 5E**; **Table 3**), which mainly are related to redox potential, energy production, protein/amino acid and lipid metabolism have also been significantly down regulated in the imprisoned yeasts. Those are Superoxide dismutase [Cu-Zn], that converts superoxide radicals (O2-) into molecular oxygen (O2) and hydrogen peroxide (H2O2), which is further converted to water (H2O) by catalase, an enzyme that contains four iron-containing heme groups; the allosteric enzyme Pyruvate kinase (C1G2W2), that provide pyruvate to be converted to acetyl-CoA in mitochondria, to gluconeogenesis and to amino acid anabolism; succinyl-CoA synthetase alpha subunit (C1G294), which is part of an enzyme that regulates citric acid cycle by controlling the interconversion between succinyl CoA and succinate and the GTP/ATP production at substrate level, and to provide precursors for porphyrin and heme biosynthesis; enoyl-CoA hydratase (C1G2P3) for beta-oxidation, that provide acetyl-CoA to citric acid cycle, but also intermediates for the metabolism of lipids, cholesterol and ketone body; and, Aspartate aminotransferase (C1G3V5), that catalyses the interconversion of aspartate and α-ketoglutarate to oxaloacetate and glutamate, important for both amino acid synthesis and for providing intermediates for the citric acid cycle/energy production; and enzymes in amino acid metabolism, such as aspartate aminotransferase, 3-oxoacid CoA-transferase (C1GBD8) and 1 -pyrroline-5-carboxylate dehydrogenase (C1GBT4). Protein synthesis, degradation and folding have also been sharply affected in granulomatous yeasts as seen by the down regulation of two different peptidases, the Carboxypeptidase Y homolog A (EC 3.4.16.5) and the Vacuolar aminopeptidase I; the molecular chaperone DNAK (C1G0P0); the 40S ribosomal protein S14 (C1G0E5); and the pre-mRNA-splicing helicase BRR2 (C1G5P0). Finally, proteins involved in vesicle transport, such as coatomer subunit alpha (C1GAF5), importin subunit alpha-6/7 (C1FZ88) and phosphatidylinositol transfer protein SFH5 (C1GJS2) have also been decrease in abundance.

We hypothesize that the fungi inside the granulomas avoid energy loss by lowering protein synthesis, since these are one of the most consuming energy processes in a cell. At the same time there is a decrease of protein degradation in an attempt to tune the protein content to adapt the general metabolism to the constraint of the granulomatous environment.

We should keep in mind that although granulomatous yeasts have lowered their protein content in general, with 1037 proteins that have not been detected (either because they were not present or were below the detection threshold of the proteome analysis), the protein that have been detected were those that the yeast maintained because somehow they are important to cope with the constraint during the host-parasite interaction, are required to resist to/modulate of immune response, to keep yeast viability and competence for proliferation and dissemination within host. These assumptions make the proteins identified in this work as promising target for drug design and screening for inhibitors.

Additionally, the absent proteins in the infection groups were also related to gene/protein regulation, amino acid metabolism, energy metabolism, and cell cycle and transport (**Figure 5F**), which support a reprogramming of these biochemical processes by the imprisoned yeasts, indicating a possible fungal low activity within the granuloma, but with their virulence preserved, as shown in the next session.

### Proteome of *Paracoccidioides brasiliensis* yeasts recovered from the granuloma indicates an enhanced metabolism concomitant with the expression of virulence factors after cultivation

The analysis of differentially expressed proteins shows that there is a large number of upregulated proteins. When yeasts from 8-week granulomatous lesions were cultivated and compared with control yeasts, 382 proteins were found to be induced and only 1 repressed. Similarly, when comparing the fungal proteins from granulomatous lesions at 12 weeks of infection, 319 proteins were found to be more abundant (**Supplementary Figures 3A–C**).

Opposing to what was observed for RNAseq and the proteome of the yeasts present in the lesions, a greater number of differentially abundant proteins were observed in the post-infection groups when compared to control group. Those abundant proteins have functions related to gene/protein regulation, energy metabolism, lipid and amino acid metabolism (**Supplementary Figure 3D**; **Supplementary Table 11**).

**Figure 6:**
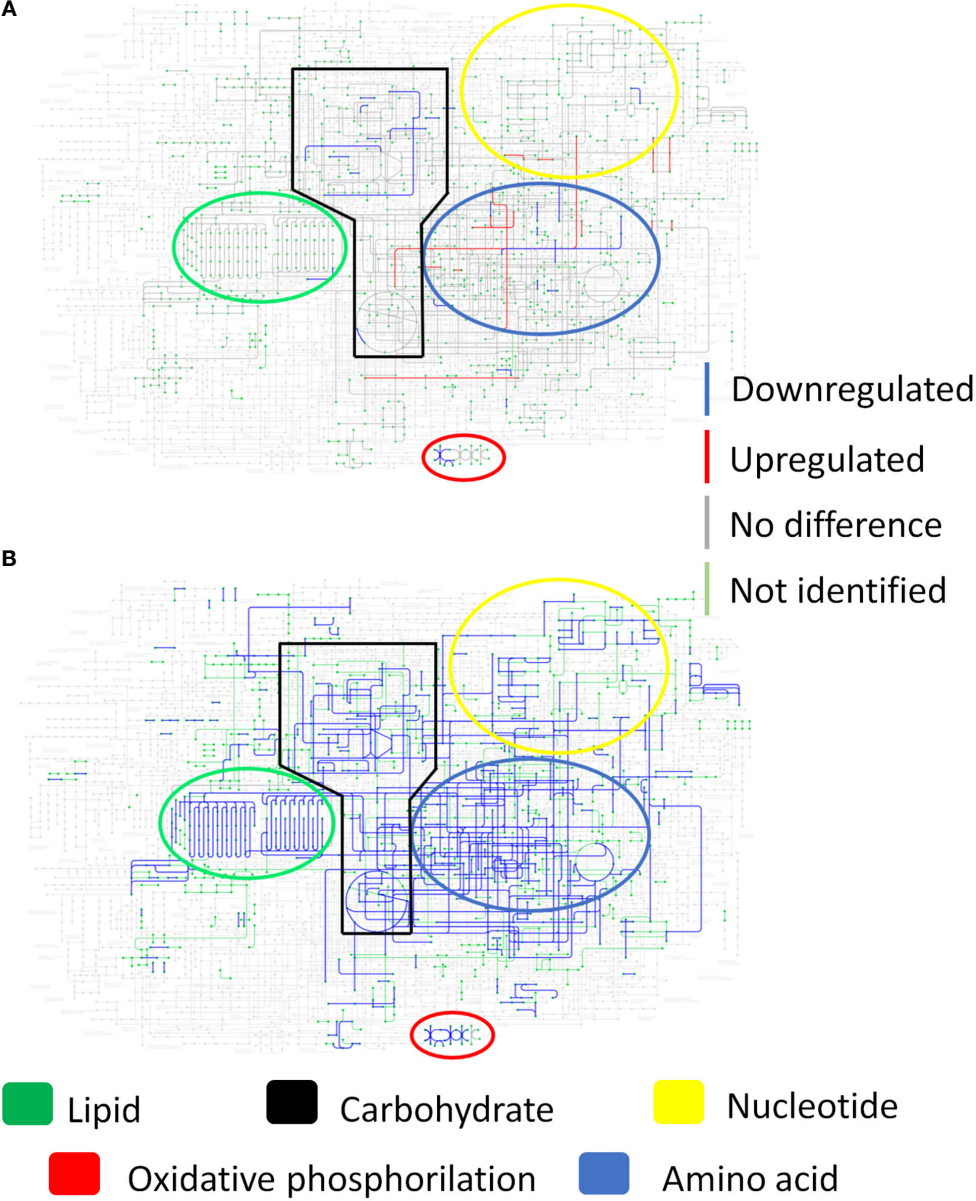
Yeast metabolism in transcriptional and proteomic levels show a repressed metabolism within the granuloma. Granulomatous lesions were extracted from two to four mice from three independent infections at eight weeks and twelve weeks after being infected with 1 x 10^6^ *P. brasiliensis* yeasts. After extracting total RNA and proteins, alignment and protein identification were carried out, respectively. The *limma* software was used to evaluate differentially abundant genes and proteins, and then the metabolic map of *P. brasiliensis* obtained from KEGG was used to evaluate the induction and repression of metabolism. **(A)** Metabolic map at the transcriptional level. **(B)** Metabolic map at protein level. Genes and proteins marked in blue are downregulated, those marked in red are upregulated, those marked in gray are not at statistically different levels, while those marked in green were not identified in the samples.

**Figure 7:**
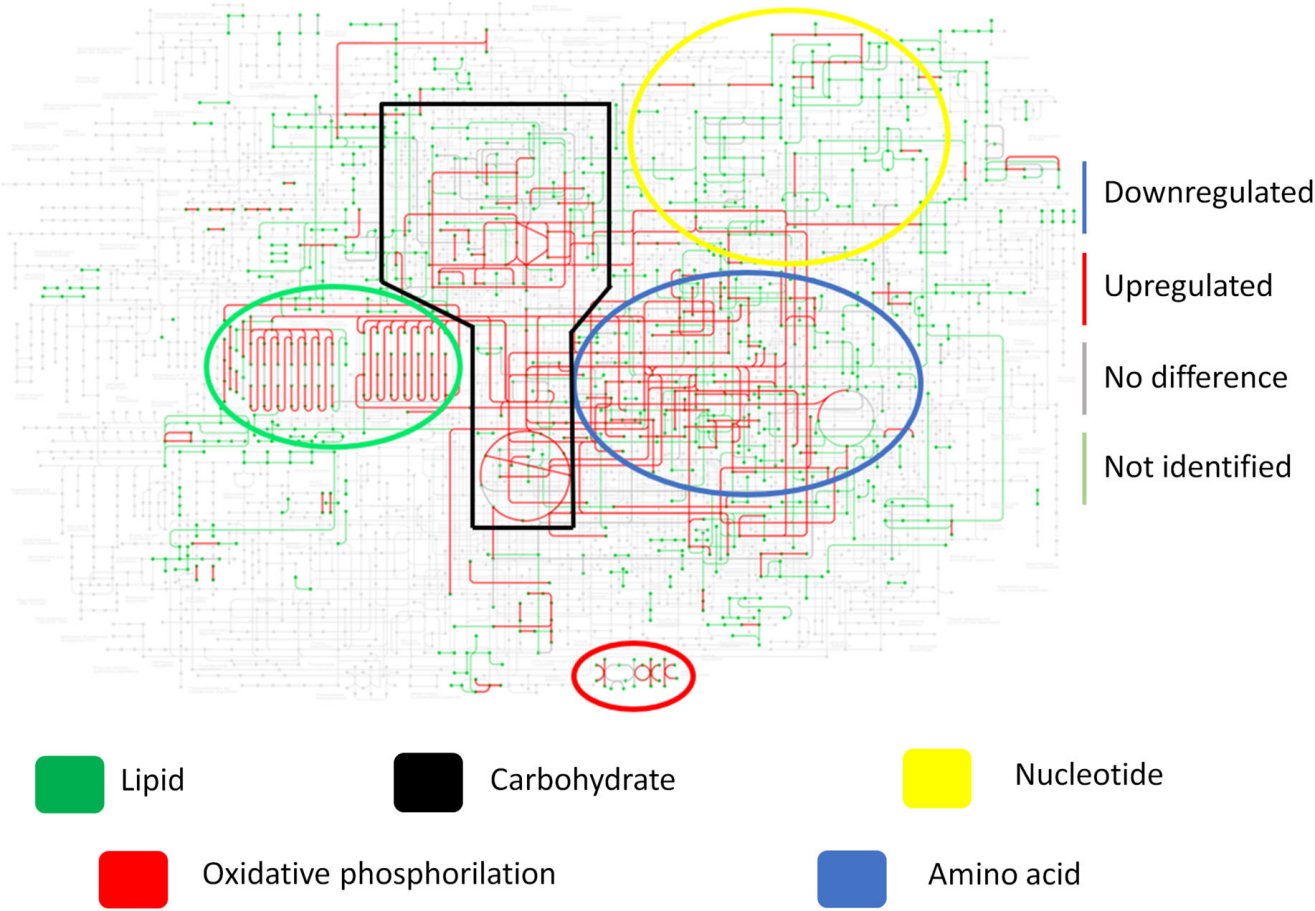
Fungal metabolism of recovered yeasts shows a recovered and enhanced metabolism after cultivation. Granulomatous lesions were extracted from two to four mice from three independent infections at eight weeks and twelve weeks after being infected with 1x10^6^ *P. brasiliensis* yeasts. After recovering the fungi present in the granulomatous lesions, cultivation was carried out in Fava Netto culture medium for 14 days. Then, extraction of fungal proteins was performed, which were digested with trypsin and resolubilized in 0.1% formic acid. Peptides were analyzed by LC-MS/MS and proteins identified by MaxQuant software. Proteins containing more than two unique peptides had their intensity normalized by log2, then quantile normalized within each experimental repeat. The *limma* software was used to evaluate differentially abundant proteins, and then use the metabolic map of *P. brasiliensis* obtained from KEGG to evaluate the induction and repression of metabolism as a whole. Proteins marked in blue are downregulated, those marked in red are upregulated, those marked in gray are not at statistically different levels, while those marked in green were not identified in the samples.

To better illustrate the yeast metabolism present in the granuloma when compared to the recovered yeasts (cultivated in Fava Netto medium), the **Figure 6A** shows the KEGG metabolic pathway of the *P. brasiliensis in* a transcriptional level, while **Figure 6B** shows the metabolic pathway at a proteomic level. This data indicates that there is a repression of the metabolism, especially the energy metabolism, both at transcriptional and proteomic levels in granulomatous yeasts. Instead, when we analyze the metabolic pathway of the recovered yeasts (**Figure 7**) we can see that the metabolism that was once repressed it is now induced, evidenced by the upregulation of lipid, nucleotide, energy, carbohydrate, and amino acid metabolisms after yeast cultivation.

### RT-qPCR analysis validates host-pathogen transcriptomics

RT-qPCR analysis was performed to validate the dual-RNAseq results for both mouse (**Supplementary Figure 4**) *and P. brasiliensis* genes (**Supplementary Figure 5**). For the host validation, we used Cxcl9, Tnf, Gbp2b, Pdcd1, Tnnc1, Lcn2, and Hamp. For the pathogen validation, we used PADG_00104 (Oxidoreductase OXR1), PADG_00097 (L-ornithine N5-oxygenase SidA) and PADG_00099 (Acyl-CoA ligase SID4), PADG_05660 (fungal nitric oxide reductase), and PADG_01954 (superoxide dismutase, Fe-Mn family). Both datasets were validated by RT-qPCR data, supporting the findings in the RNAseq with a Pearson statistical correlation between 0.8981 and 0.9679 for the mice dataset and between 0.8906 and 0.906 for the *P. brasiliensis* dataset.

### *Paracoccidioides brasiliensis* yeasts present in the granulomatous lesions produces lower levels of intracellular siderophore ferricrocin and has low lipid content.

**Figure 8:**
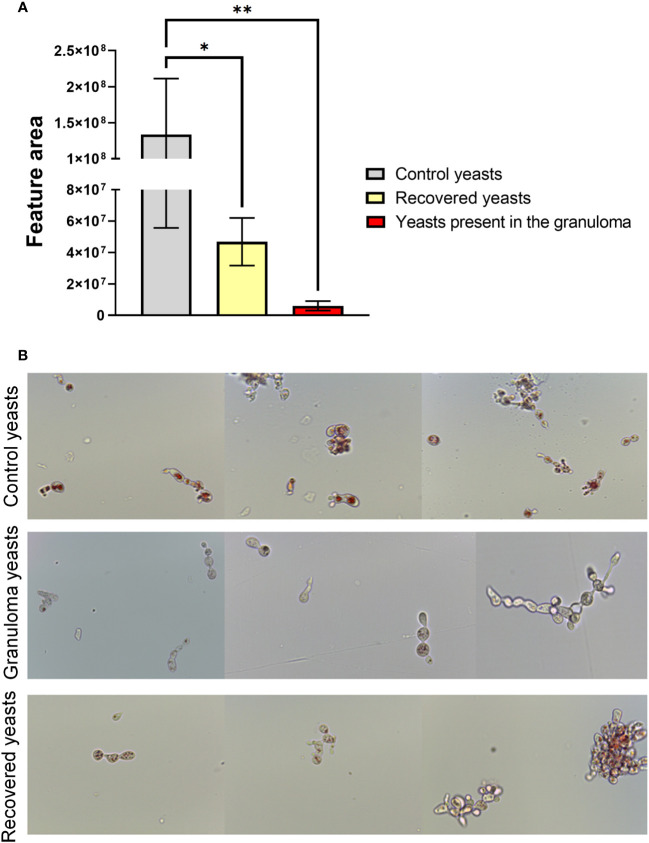
Yeasts present in the granuloma produces low levels of intracellular siderophore ferricrocin and has low lipid availability. For the ferricrocin levels analysis **(A)** a quadruplicate of lung granuloma, control lung, control yeasts and recovered yeasts were frozen, and 100 mg of frozen material was macerated with the help of a pestle and pot. The secondary metabolites extraction was performed with 1 mL of methanol (100%; HPLC grade), followed by sonication bath for 40 minutes. For sample preparation, 500 μL of each sample extract were filtered in a 0.22 μm PTFE and diluted to 1 mL volume with HPLC-grade methanol. Then LC-HRMS/MS analysis were performed and data were processed with Xcalibur software (version 3.0.63). Statistical analysis was performed using one-way ANOVA. Bars represent means ± standard error of the feature area of groups of 4 to 5 samples (*p<0.05; **p < 0.01). For lipid availability analysis **(B)** control yeasts, granuloma yeasts and recovered yeasts were stained with filtered Oil Red O for 2 hours at room temperature. Subsequently, the cells were washed twice with ultrapure water and then resuspended in PBS for analysis on a microscope slide. The photos were taken using the Leica DM750 microscope and the Leica Application Suite V4.8 software, thus allowing the observation of stained neutral lipids inside the cells.

To verify the expression of siderophores by *P. brasiliensis*, LC-HRMS/MS analyses were performed with the granuloma fungus extracts, control fungus and recovered fungus. The siderophore Ferricrocin (*m/z* 718.3370) was annotated based on its exact mass and MS/MS fragmentation pattern typical of this siderophore class. Ferricrocin levels were lower in the yeasts present in the granuloma and the recovered ones when compared to the control yeasts (**Figure 8**). This result corroborates the RNAseq data considering a lower expression of SidL observed in the granuloma yeasts (**Figure 4E**; **Table 2**).

Furthermore, a OilRed O lipid staining was performed aiming to evaluate the neutral lipid content in the three yeasts groups (control, recovered and present in the lesions). Oil Red O dye binds to neutral lipids, as those found in lipid droplets, but not to phospholipids in membranes, revealing the amount and size of lipid droplets by staining them with a red colour. As showed in the **Figure 8B**, the lipid droplets in the infection yeasts are less abundant when compared to control yeasts, supporting both the transcript and proteomic data, where genes and proteins related to beta-oxidation were significantly less abundant in the fungus inside the granuloma. Because lipid droplets are in greater number and size in the recovered and cultured yeasts compared to the yeasts present in the lesions, our results indicate 1) neutral lipid stocks decrease during infection and 2) that lipid biosynthesis is resumed by subsequent cultivation of yeast isolated from the lesions in rich medium.

### Yeasts isolated from granulomas are more virulent than those of non-granuloma yeasts

**Figure 9:**
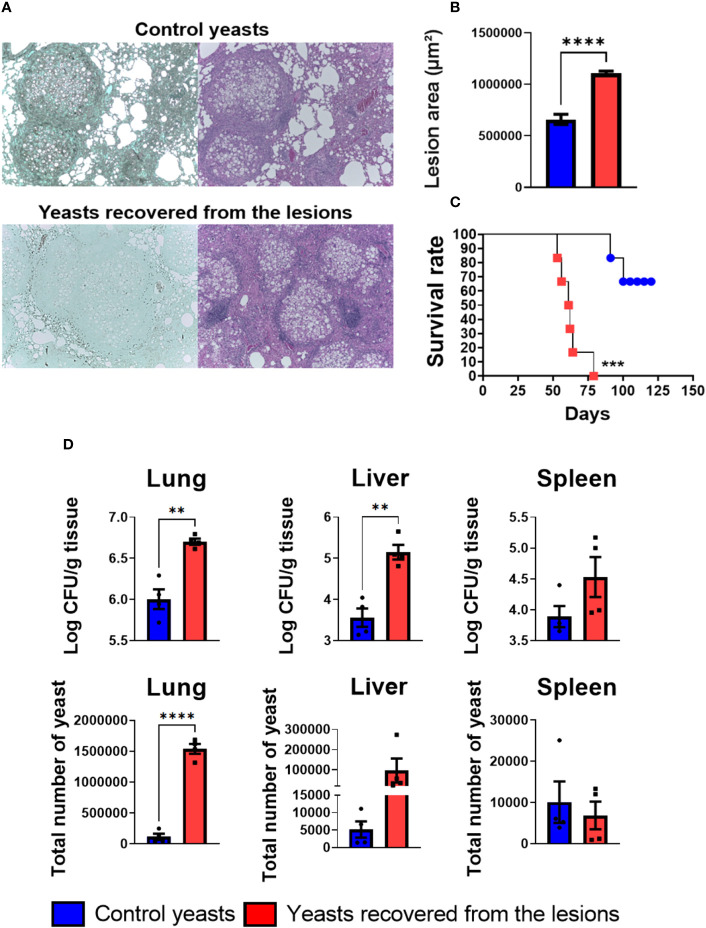
Recovered yeasts induces looser granulomas, is more virulent and disseminate more than control yeasts. Groups of four C57BL/6 mice were infected by i.t. route with 1 × 10^6^ control *P. brasiliensis* yeasts or recovered yeasts contained in 50 µL of PBS. After eight and weeks of infection, the animals were euthanized and the lungs removed, which were stored in 10% formaldehyde at 4 °C. Sections of 5 µm were stained with Hematoxylin-Eosin (H&E; pink) for analysis of lesions and stained with Grocott (green) for fungal evaluation **(A)**. The anatomy of the lesion was analyzed according to size, morphology, and presence of fungal cells. The image and the lesion area calculation were taken using the Leica DM750 microscope and the Leica Application Suite V4.8 software. Statistical analysis was performed using one-way ANOVA. Bars represent means ± standard error of lesion area (µm²) of groups of three mice **(B)**. For the survival analysis **(C)** groups of six C57Bl/6 WT mice were infected via i.t. with 1x10^6^ yeasts extracted from granulomatous lesions or control yeasts to verify the virulence of freshly extracted granuloma yeasts. Deaths were recorded daily. The survival curve used the Log-rank test (Mantel-Cox) to compare the two groups. For the CFU assay **(D)** groups of three to four C57BL/6 mice were infected by the i.t. route with 1 × 10^6^ *P. brasiliensis* yeasts contained in 50 µL of PBS. After eight weeks of infection, the lungs, liver, and spleen were removed and macerated. After that, the samples were centrifuged to obtain the fungus and inoculated in plates containing BHI medium for subsequent counting of CFU. Bars represent means ± standard deviation of log10 or total yeast number. Values were considered significant when **p < 0.01; ***p<0.001; ****p<0.0001.

Aiming to compare the virulence of the yeasts obtained from the granuloma after 8 weeks of infection with the non-granuloma yeasts (control yeasts, i.e, yeasts used for mice infection), a histopathology analysis of the lung was made. The mice infected with the yeasts isolated from granulomatous lesions had a less compact granuloma with a larger lesion area when compared to the granulomas size formed in mice infected with the control yeasts (**Figures 9A, B**). Furthermore, a survival analysis of infected C57BL/6 wild-type (WT) mice was performed. All mice infected with granulomatous lesion-yeasts died within 79 days; in contrast, mice infected with control yeasts start to die on day 91 and 100 days after infection only two mice were dead (**Figure 9C**). These results demonstrate that yeasts recovered from granulomatous lesions are more virulent than the non-granuloma control yeasts. To evaluate the fungal load a CFU assay was performed with lungs, liver, and spleen of mice. As it can be seen in the **Figure 9D**, the mice infected with the recovered yeast had a higher fungal load in the lungs and liver when compared to animals infected with control yeasts, showing that the yeasts from granulomatous lesions can disseminate more easily than control yeasts.

## Discussion

The granuloma is a host defense mechanism that aims to contain the pathogen and prevent the spread of infection. However, at the same time that the granuloma keeps the infection under control, allows the pathogen to survive inside, since it does not eliminate it completely (Rubin, 2009; Hams et al., 2013).

The functional analysis of the DEGs of the infected host tissue indicates the expression of genes associated with innate and adaptive immunity. To our knowledge, it is the first time that both PRRs Clec4d and Clec4a2 were observed in chronic PCM, although Dectin-3 (Clec4d) expression was demonstrated *in vitro* in human plasmacytoid dendritic cells (pDCs), where it has been associated to type I IFN and IL-1β secretion (Preite et al., 2018). Besides, Dectin-3 has been described to initiate a host defense against *Candida albicans* and *Cryptococcus ssp* (Zhu et al., 2013; Huang et al., 2018). Clec4a2 belongs to the Dectin-2 family, and it is also named DC immunoreceptor (DCIR) (Bates et al., 1999; Lambert et al., 2008; Kerscher et al., 2013; Bloem et al., 2014; Nagae et al., 2016) In tuberculosis, DCIR was reported to be protective by modulating the immune response through IL-12 expression and Th1 expansion (Troegeler et al., 2017). The expression of Pentraxin 3 (Ptx3), a PRR related gene stimulated by tumor necrosis factor and interleukin-1beta, which remains underexploited in PCM, has already been reported to be involved in the *P. brasiliensis* opsonization by macrophages (Diniz et al., 2004). Additionally, Dectin-1 (Clec7a) and TLR2 important PRRs in PCM and others mycosis were also observed with a transcript expression in the granuloma. These receptors play an important role in the immune response of human and murine phagocytic cells when exposed to *P. brasiliensis* infection (Brown, 2006; Bonfim et al., 2009; Loures et al., 2009; Loures et al., 2014; Feriotti et al., 2015; Calich et al., 2019; de Quaglia E Silva et al., 2019; Li et al., 2019; Rodriguez-Echeverri et al., 2021).

The transcripts to chemokines Ccl3 (MIP-1α), and Ccl7 (MCP3), that have never been described in a PCM context, were also expressed in the granuloma. Those chemokines are responsible for recruiting monocytes, neutrophils, DCs, and eosinophils, which reflects the presence of these cells within the lesions (Luster, 1998). In fact, Ccl3 and Ccl7 were associated to a milder *Cryptococcus neoformans* and *Histoplasma capstulatum* infection, since they promote a protective profile by inhibiting a Th2 response (Huffnagle et al., 1997; Olszewski et al., 2000; Szymczak and Deepe, 2009; Qiu et al., 2012). Additionally, the expression of Ccl3 and Ccl4 (MIP-1β), a chemokine responsible for monocytes, neutrophils, DCs, and eosinophils migration has already been related to neutrophil accumulation in the lungs in *Aspergillus fumigatus, C. neoformans, H. capsulatum and P. brasiliensis* infection, indicating an important neutrophil participation in the granuloma (Huffnagle et al., 1997; Mehrad et al., 2000; Olszewski et al., 2000; Souto et al., 2003; Kroetz and Deepe, 2010; Puerta-Arias et al., 2016). Since Ccl3, Ccl4, and Ccl7 attract eosinophils, these cell populations are also expected in the granulomatous lesions. Although eosinophils are common in the acute PCM form, they have already been identified in the lesions by the presence of the eosinophil granule major basic protein, but their function in the chronic form of PCM remain unclear (Wagner et al., 1998; Braga et al., 2017).

The presence of Ifng, Tnf, Tnfrsf9, and Nos2 transcripts (iNOS) corroborates with the fact that macrophages and a Th1 response play an essential role in the granuloma function. TNF-α is responsible for macrophages accumulation and differentiation in epithelioid cells, resulting in a well-organized granuloma (Kindler et al., 1989; Figueiredo et al., 1993). Therefore, the expression of TNF-α can explain why the granulomas were still tight in our histology analysis. Furthermore, IFN-γ can activate infected macrophages to produce TNF-α and iNOS, leading to a reduced *P. brasiliensis* replication (Brummer et al., 1988; Gonzalez et al., 2000), which may explain why yeasts transcripts and proteins related to cell cycle are in a lower level inside the granuloma when compared to control fungi.

Concomitant to the presence of macrophages and Th1 lymphocytes, there is an indication that neutrophils and Th17 lymphocytes also play an important role in the granuloma activity. The Th17 response, although described as a crucial T cell subset in granuloma formation, it has never been described inside a chronic PCM granulomatous lesion (Tristão et al., 2017). However, IL-17 expressing cells were found in the skin and mucosal lesions of patients (Pagliari et al., 2011) besides a protective role of Th17 cells that has been described by our group (Galdino et al., 2018; Preite et al., 2023). The depletion of myeloid-derived suppressor cells (MDSCs) as well as Treg cells leads to robust Th1/Th17 lymphocyte responses associated to a regressive disease with reduced fungal burden, lung pathology and mortality ratios in mice (Galdino et al., 2018; Preite et al., 2023).

As reported to several disease, controlling the immune response is crucial, aiming for a homeostasis of the inflammatory response, as an exaggerated immune reaction can result in host damage and an inhibited immune response as well (Marques et al., 2016). The expression of Arginase-1 and collagenase 3 (Mmp13) indicates a well-regulated immune response with tissue repair activation and the participation of M2 polarized macrophages which are related to an anti-inflammatory profile (Elkington et al., 2005; Yunna et al., 2020). M2 macrophages have already been linked to a less severe PCM, since a M2 profile was found in the immunoregulatory mechanism developed by resistant mice (Feriotti et al., 2013). Mmp13 was described to cleave some chemokines, such as Ccl2 and Ccl7, leading to a negative control of chemotaxis and inhibiting the fibrosis formation in the PCM granuloma (Arango et al., 2017; Giannandrea and Parks, 2014). Pdcd1 (PD-1) and CTLA4 induction in the granuloma may suggest that these immune checkpoint inhibition molecules play a role in the lymphocyte’s homeostasis, controlling the Th1/Th17 responses (Sansom, 2000; Keir et al., 2008). In fact, those two receptors have been explored as a drug target in some respiratory infections (Hamashima et al., 2020). In a murine model of *C. neoformans* infection, the administration of anti PD-1 and anti CTLA-4 antibodies resulted in a better survival of the mice (McGaha and Murphy, 2000; Roussey et al., 2017).

Although the host develops a highly active and regulated immune response inside the granuloma, an impaired function of the lung was here detected, since transcripts related to the normal function of lung were found in lower levels within the lesions when compared to *naïve* control lung, as revealed by the enrichment analysis of the downregulated genes. The low abundance of these genes can also be explained because the granuloma is mostly composed of immune and yeast cells, so there are less healthy lung and consequently, less transcripts. However, since the immunological structure is occupying almost the whole lung, it is safe to assume an impaired lung function. In fact, in chronic obstructive pulmonary diseases, the expression of myosin is impaired, due to the shortening of the telomere (Thériault et al., 2012; Thériault et al., 2014; Pomiès et al., 2015).

To our knowledge, this is the first time a transcriptional study has been conducted within the murine granuloma in PCM. For this reason, we presented several genes and mechanisms that can be explored in the future. The presence of transcripts within the lesions does not guarantee that these responses are active, as post-translational modifications and regulatory mechanisms can hinder the translation of these transcripts into proteins. Therefore, the results presented so far serve to guide future studies on the immunology of PCM.

The functional analysis of the differentially expressed genes of the fungus shows an upregulation of genes related to the synthesis and transport of the siderophores fusarinine C and triacetylfusarinine C. This can be considered a very interesting data, considering that it has already been described that a greater supply of iron to the fungus is related to its higher virulence (Parente et al., 2011; Silva et al., 2020). The upregulation of these genes related to extracellular siderophores may indicate a lack of iron within the granuloma (Timm et al., 2003; Hilty et al., 2011; Haas, 2014). In fact, previous studies have shown that *P. brasiliensis* cultivated in iron deficient medium expressed transcripts related to extracellular siderophores (Parente et al., 2011). The sidL analogue (N5-hydroxyornithine acetyltransferase (PADG_07849) was found downregulated in the infection. This gene is part of the synthesis pathway of the intracellular siderophores ferricrocin and hydroxyferricrocin, responsible for intracellular storage and distribution of iron (Schrettl et al., 2007; Blatzer et al., 2011). This repression of SidL may indicate that there is no iron available for storage by the fungus within the granuloma (Miethke and Marahiel, 2007; Schrettl et al., 2007). Interestingly, the phenomenon of increased synthesis of siderophores for iron uptake and repression of synthesis of siderophores for maintenance within the granuloma was previously described for *M. tuberculosis* Timm et al., 2003). However, the expression of siderophores by *P. brasiliensis* present in the granuloma have never been described in PCM. The identification of ferricrocin by LC-HRMS/MS in lower levels within the granuloma strengthen the fact there is an iron deficiency by the pathogen inside the immunological structure (Haas, 2014).

Another fact that indicates there is an iron deficiency within the granuloma, is that the host induces the expression of Lipocalin 2 (Lcn2), a protein that binds to siderophores chelated to iron, helping the defense against siderophores expressing pathogens, acting as an iron sequestrator and as a bacteriostatic agent (Goetz et al., 2002; Holmes et al., 2005). Lipocalin 2 has also been described as an important player in the tuberculosis granuloma formation. LcnKO mice infected with *M. tuberculosis* had increased granulomatous inflammation when compared to WT infected mice, because Lcn2 has the ability to induce neutrophil migration and lymphocytes regulation (Guglani et al., 2012). High levels of Lcn2 transcripts were also reported in *C. neoformans* and *A. fumigatus* infection (Gessner et al., 2012; Wozniak et al., 2014). Inhibiting the fungal intracellular iron is crucial for host survival. Previous reports in which the authors used chloroquine to restrict intracellular iron in *P. brasiliensis* yeasts, was reported a lower fungal load inside human and mouse monocytes when compared to cells that did not receive chloroquine treatment (Dias-Melicio et al., 2006). These results, along with our data, indicate that iron is crucial for fungal survival and replication in the host.

The repressed expression of Hamp can indicate that there is a lack of iron even for host, because this hormone binds to ferroportin, leading to its internalization and degradation, causing an inhibition of iron efflux (Nemeth et al., 2004; Nemeth and Ganz, 2021). Because this hormone cause host cell to trap the iron inside and iron is a major player in the course of several diseases, an upregulation was observed in many infections, such as those caused by *M. tuberculosis*, *Vibrio vulnificus*, *A. fumigatus* and *Fusarium oxysporum* (Leal et al., 2013; Arezes et al., 2015; Harrington-Kandt et al., 2018). Therefore, the repressed expression of hepcidin in a chronic PCM context, may indicate that there is an iron deficiency for both the host and the pathogen, revealing iron to be a crucial element for the granuloma function. In fact, A recent work showed that patients with chronic PCM developed anemia due to iron deficiency and due to inflammation process (de Brito et al., 2023).

An induction of some genes that have been described as *P. brasiliensis* virulence factors in other contexts were also found. Superoxide dismutase Cu-Zn (PADG_02842) has been described to help against the oxidative stress caused by the synthesis of reactive oxygen species by the host. Likewise, alcohol dehydrogenase (PADG_04701) synthesis has been reported to help against the increase in pyruvate released by amino acid degradation (Castilho et al., 2014; Camacho and Niño-Vega, 2017). Another transcript that is a possible virulence factor is CPS1 (polysaccharide synthase; PADG_04274), which has been described in *C. neoformans* and contributes to the pathology, since it is associated with the rigidity of the cell wall (Chang et al., 2006). These transcripts related to virulence factors that were found upregulated do not use iron as a cofactor, while some described in lower transcriptional and proteomic levels use iron as a cofactor, strengthening the hypothesis of iron deficiency in the granuloma.

The analyzes of repressed genes and proteins by the yeasts inside the granulomatous lesions revealed a large number related to transcriptional factors. The reduced expression of these genes and proteins favors the idea that the fungus is repressing a good part of its gene expression within the granuloma, which can explain the low protein identification in the lesions yeasts when compared to the control group. It is important to emphasize that these transcripts that have been modulated to increase or decrease in abundance may not be directly involved in the maintenance of the low metabolic activity state of the fungus or in coping with the host-imposed constrains because their protein products were not identified in the proteome. Instead, keeping these transcripts may be related to the fungus ability to recover from the constrain and to prepare them to resume full metabolism once free.

The energy production of the fungus within the granuloma is also repressed since there are genes and proteins related to energy metabolism downregulated, such as those related to glycolysis, beta oxidation, Krebs cycle, and oxidative phosphorylation, including Cytochrome b5 heme-binding domain-containing protein. Iron is essential for the survival of the fungus, so siderophores synthesis is a way to circumvent iron deficiency. Another way to control iron availability is by altering their metabolism, limiting the synthesis of proteins that use iron as a cofactor, as is the case of some proteins related to oxidative phosphorylation and Krebs cycle (do Amaral et al., 2019). In previous studies, *P. brasiliensis* cultivated in iron deficient medium had also repressed proteins related to Krebs Cycle, glyoxylate cycle and oxidative phosphorylation (Parente et al., 2011). This report supports the hypothesis of iron deficiency inside the granuloma.

In addition, multiple proteins, including the PLK1 (PADG_03022) and serine/threonine-protein gene TTK/MPS1 kinase (PADG_11709), which are essential for the continuation of mitosis were also detected in low levels. Another fact that corroborates with the repression of fungus reproduction is that energy production and amino acid metabolism are repressed, which are essential for reproduction (Zhu and Thompson, 2019). Additionally, a limitation in pathogen growth within the murine lung and the granuloma was previously described for *M. tuberculosis*, which enters a latent state after a long period of infection, mostly in more resistant mice (Wayne and Sohaskey, 2001; Shi et al., 2003). The latent state in *M. tuberculosis* was associated to hypoxia conditions and nutrient starvation, which lead to a low energy and lipid production, corroborating with our findings and indicating a possible nutrient starvation by the fungus (Betts et al., 2002). Although there is a repression of the fungus reproduction, the pathogen is still able to grow inside the granuloma, as seen from the histological analysis, revealing a fight between the pathogen and host, in which the pathogen succeeds.

Interestingly, a low expression of 15 cell cycle genes associated with fungal reproduction were here detected. Therefore, our findings lead us to speculate that fungi are with low metabolic activity, when metabolism must be reprogrammed in order to fit yeast cells to such a macro and micro-nutrient restrictive environment. We even hypothesized that the fungus is suppressing the protein production to save energy loss, only expressing proteins that are essential for survival. Nevertheless, the fungus succeeds in infection: it disseminates within the lung and to other tissues/organs after 12 weeks of infection and eventually kill the host. Importantly, the low activity state should not be interpreted as dormancy, but as a delay of this fungus to reach widespread dissemination when compared to its behaviour in a fully susceptible host, in which PCM causes death much faster. We also speculate that the metabolic fitness may further protect yeasts against the host immune response, albeit we cannot rule out the possibility that host immune response also contribute to shape yeast adaptation within granulomas of susceptible mice. The fact is that the host-pathogen relationship somehow enables yeast cells to successfully recover from the restrictive condition, and even burst, their virulence once released from the constrains inflicted by the host, as seen from the proteome of the recovered yeasts. This data agrees with previous reports where it was shown that after several animal passages the antioxidant repertoire of the recovered *P. brasileinsis*-yeasts had changed in comparison with control avirulent yeasts (Castilho et al., 2014; Castilho et al., 2018). Looking from the parasite perspective, survival within granulomas by *Paracoccidioides* sp. and *M. tuberculosis* require adaptation to iron deficiency, nutrient starvation, and hypoxia. Instead, adaptation to low pH and nitrosative stress reported in the bacterial granuloma may not be relevant in PCM granuloma (Timm et al., 2003; Talaat et al., 2007; Garton et al., 2008).

The results obtained from the basic lipids staining support the analyzes of the fungal transcriptomics and proteomics data that show a wide reprogramming of the lipid metabolism. Because a reduction in abundance of genes and proteins related to beta-oxidation within the granuloma was seen and because some of the enzymes can act either way to catabolism or anabolism depending on carbon source availability, the decrease of lipid droplets abundance in yeast inside the granuloma clearly show that the basic lipid catabolism rates were actually higher than lipids’ synthesis rate at this site. Indeed, after 6 hours of infection there is an increase in beta-oxidation by the yeasts, as well as an increase in the glyoxylate cycle, causing consumption of the yeast’s lipid stock (Lacerda Pigosso et al., 2017).

Taken together our data demonstrate an important iron deficiency for both the host and the pathogen inside the fungal granuloma, concomitant with the activity of genes related to the innate and adaptive immunity of hosts. Furthermore, our data indicate that the fungus represses its metabolism and proliferation inside the granuloma, but still grow, and once it is recovered from the lesions and *in vitro* cultivated a more active metabolism is established concomitant with an elevated expression of virulence molecules. In fact, the recovered yeasts induce a looser granuloma with a larger lesion area when compared to control yeasts, concomitant with an uncontrolled dissemination as observed in the CFU analysis. Additionally, the premature death of animals infected with yeasts recovered from granulomatous lesions demonstrates the enhanced virulent profile of the recovered yeasts.

## Data availability statement

The datasets presented in this study can be found in online repositories. The names of the repository/repositories and accession number(s) can be found below: https://www.ncbi.nlm.nih.gov/, PRJNA945862 http://www.proteomexchange.org/ PXD040826 http://www.proteomexchange.org/, PXD040829.

## Ethics statement

The animal study was approved by Ethics Committee on Animal Experiments (CEUA) of the Federal University of São Paulo (UNIFESP), No. 9596270919. The study was conducted in accordance with the local legislation and institutional requirements.

## Author contributions

BB: Conceptualization, Data curation, Formal Analysis, Investigation, Methodology, Project administration, Validation, Visualization, Writing – original draft, Writing – review & editing. RR: Investigation, Methodology, Writing – review & editing, Data curation, Formal Analysis, Visualization. NP: Formal Analysis, Investigation, Methodology, Visualization, Writing – review & editing. VK: Formal Analysis, Investigation, Methodology, Visualization, Writing – review & editing. PA: Data curation, Formal Analysis, Investigation, Methodology, Visualization, Writing – review & editing. MC: Data curation, Methodology, Writing – review & editing, Formal Analysis. MM: Formal Analysis, Investigation, Writing – review & editing. TF: Investigation, Methodology, Validation, Writing – original draft, Writing – review & editing. VC: Conceptualization, Visualization, Writing – original draft, Writing – review & editing. AT: Data curation, Methodology, Writing – review & editing. OS-B: Funding acquisition, Writing – review & editing, Formal Analysis. SD: Funding acquisition, Writing – review & editing. ÖB: Funding acquisition, Methodology, Resources, Writing – review & editing. CC: Conceptualization, Formal Analysis, Investigation, Writing – original draft, Writing – review & editing. AZ: Data curation, Investigation, Methodology, Writing – review & editing. GG: Conceptualization, Formal Analysis, Investigation, Methodology, Writing – original draft, Writing – review & editing. FL: Conceptualization, Funding acquisition, Investigation, Methodology, Project administration, Resources, Supervision, Writing – original draft, Writing – review & editing.
