# Supplementary material for: Transcriptional profiling of a fungal granuloma reveals a low metabolic activity of Paracoccidioides brasiliensis yeasts and an actively regulated host immune response
Source: Front Cell Infect Microbiol. 2023 Oct 5;13:1268959. doi: 10.3389/fcimb.2023.1268959 (PMC10585178; doi:10.3389/fcimb.2023.1268959)

Supplementary Figure 1. Evaluation of pulmonary fungal burden of granulomatous lesions and apparently healthy tissue. C57BL/6 mice were infected by the IT route with 1×10^6 *P. brasiliensis* yeasts contained in 50 µL of PBS. After eight weeks of infection, the lungs were removed and the granulomatous lesions were separated from the apparently healthy lung regions, then these samples were macerated. After that, the samples were centrifuged to obtain the fungus and inoculated in plates containing BHI medium for subsequent counting of colony forming units. (A) Bars represent means ± log10 standard deviation of counts from groups of 2 mice (*$p < 0.05$). (B) Bars represent means ± standard deviation of total fungus number of counts from groups of 2 mice.

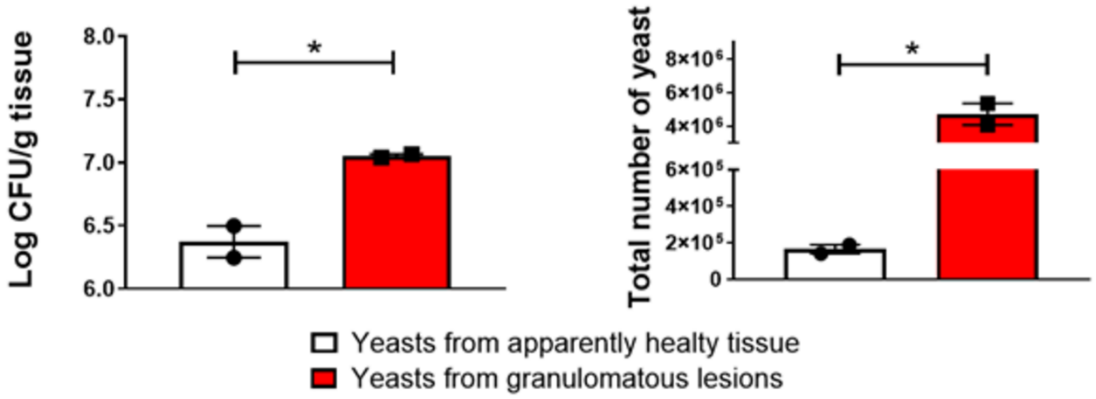

Supplementary Figure 2. ***M. musculus*** **genes found exclusively in the infection.** Functional analysis of the genes found exclusively in the infection was carried out using GO, KEGG and Panther.

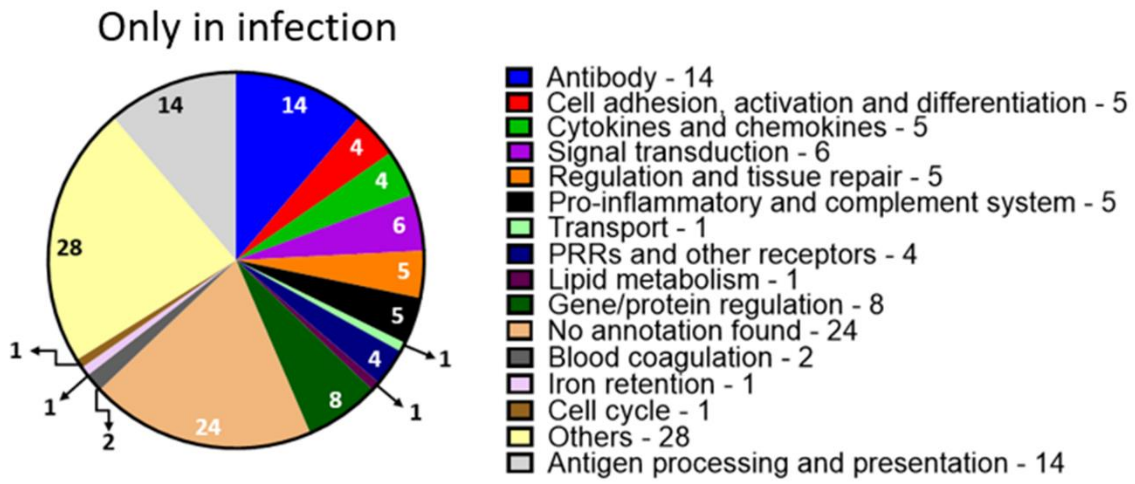

Supplementary Figure 3. Proteome of *P. brasiliensis* yeasts isolated from the granuloma indicates an enhanced metabolism. To illustrate the differentially abundant proteins, a volcano plot of the 8-weeks infection (8W) group in relation to the control yeasts (A) and a volcano plot of the 12-weeks infection group (12W) in relation to the control yeasts (B) were made. Additionally, a heatmap was created with selected proteins (C). The proteins in red represent a higher abundance and the green ones a lower abundance for a given sample. The functions of the upregulated proteins (D) were taken using GO, KEGG and FungiDB.

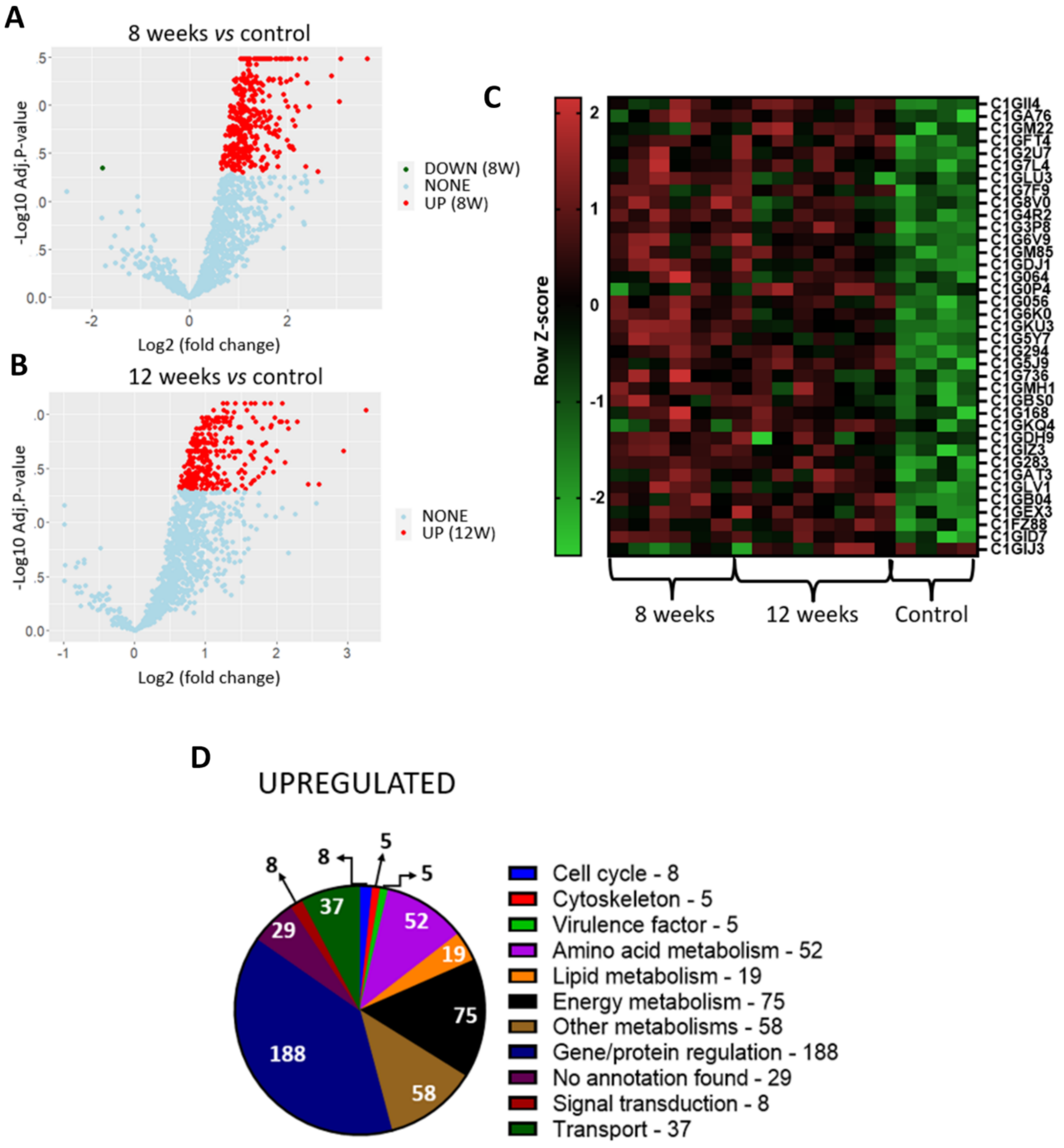

Supplementary Figure 4. Independent validation of the mice RNA-seq. Granulomatous lesions were removed from mice with eight and twelve weeks of infection, which were infected with $1x10^6$ *P. brasiliensis* yeasts. Then, total RNA extraction and DNA digestion were performed. Samples were subjected to reverse transcriptase. The synthesized cDNA was used for real-time PCR analysis using the SYBR green PCR master mix kit (Applied Biosystems) on the ABI 7500 Fast real-time PCR system (Applied Biosystems, Foster City, CA, USA). The expressions of Lcn2, Gbp2b, Pdcd1, Tnf, Cxcl9, Tnnc1 and, Hamp were evaluated using Usp9x as a normalizer. The heatmap show the log (fold change) values for the RNA-seq and RT-qPCR.

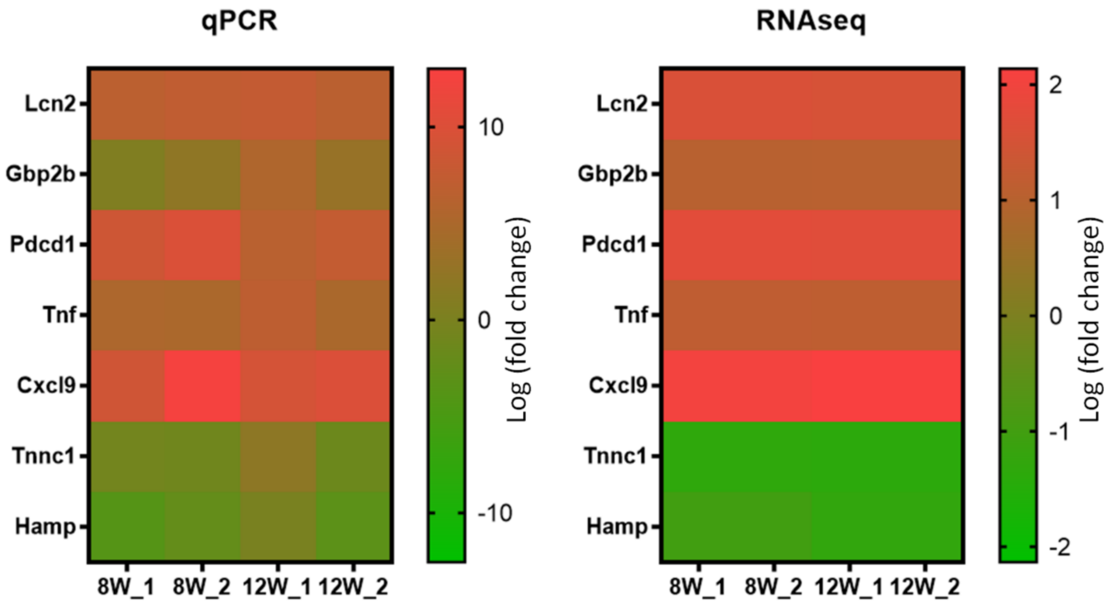

	8W_1	8W_2	12W_1	12W_2
Pearson r				
r	0,8981	0,9129	0,9591	0,9679
95% confidence interval	0,4479 to 0,9850	0,5115 to 0,9873	0,7420 to 0,9941	0,7926 to 0,9954
R squared	0,8065	0,8334	0,9199	0,9369
P value				
P (two-tailed)	0,006	0,0041	0,0006	0,0003
P value summary	**	**	***	***
Significant? (alpha = 0.05)	Yes	Yes	Yes	Yes
Number of XY Pairs	7	7	7	7

Supplementary Figure 5. Independent validation of the fungal RNA-seq. Granulomatous lesions were removed from mice with eight and twelve weeks of infection, which were infected with $1x10^6$ *P. brasiliensis* yeasts. Then, total RNA extraction and DNA digestion were performed. Samples were subjected to reverse transcriptase. The synthesized cDNA was used for real-time PCR analysis using the SYBR green PCR master mix kit (Applied Biosystems) on the ABI 7500 Fast real-time PCR system (Applied Biosystems, Foster City, CA, USA). The expressions of PADG_00104, PADG_00097, PADG_00096, PADG_05660 and, PADG_01954 were evaluated using PADG_00714 as a normalizer. The heatmap show the log (fold change) values for the RNA-seq and RT-qPCR.

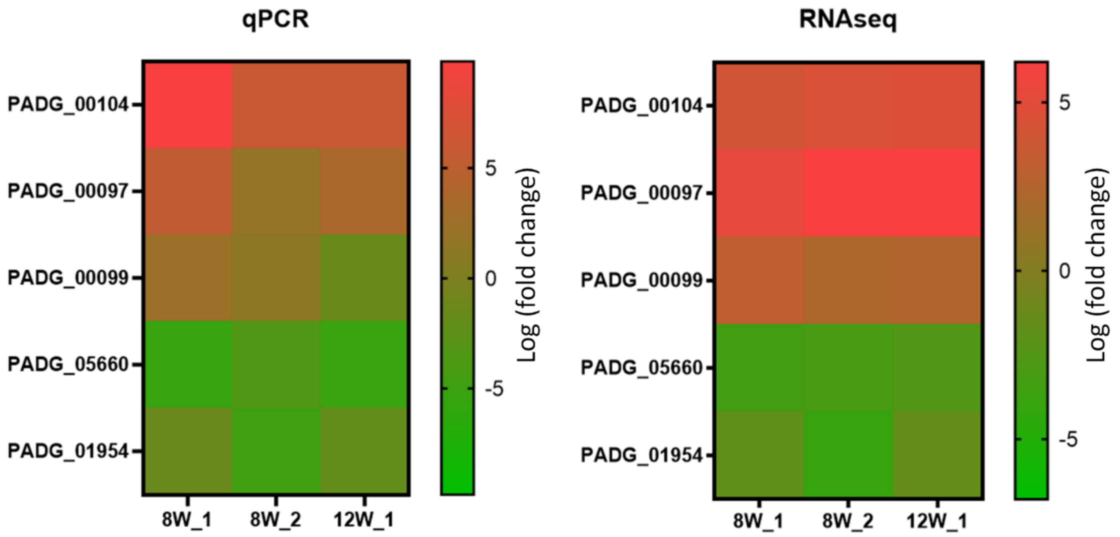

	8W_1	8W_2	12W_1
Pearson r			
r	0,906	0,8906	0,8922
95% confidence interval	0,1182 to 0,9938	0,03873 to 0,9928	0,04664 to 0,9929
R squared	0,8208	0,7931	0,796
P value			
P (two-tailed)	0,0341	0,0427	0,0418
P value summary	*	*	*
Significant? (alpha = 0.05)	Yes	Yes	Yes
Number of XY Pairs	5	5	5

Supplementary Figure 6. Intracellular siderophore identification. LC-HRMS/MS analysis were performed and data were processed with Xcalibur software (version 3.0.63). The siderophore Ferricrocin (m/z 718.3370) was annotated based on its exact mass and MS/MS fragmentation pattern typical of this siderophore class.

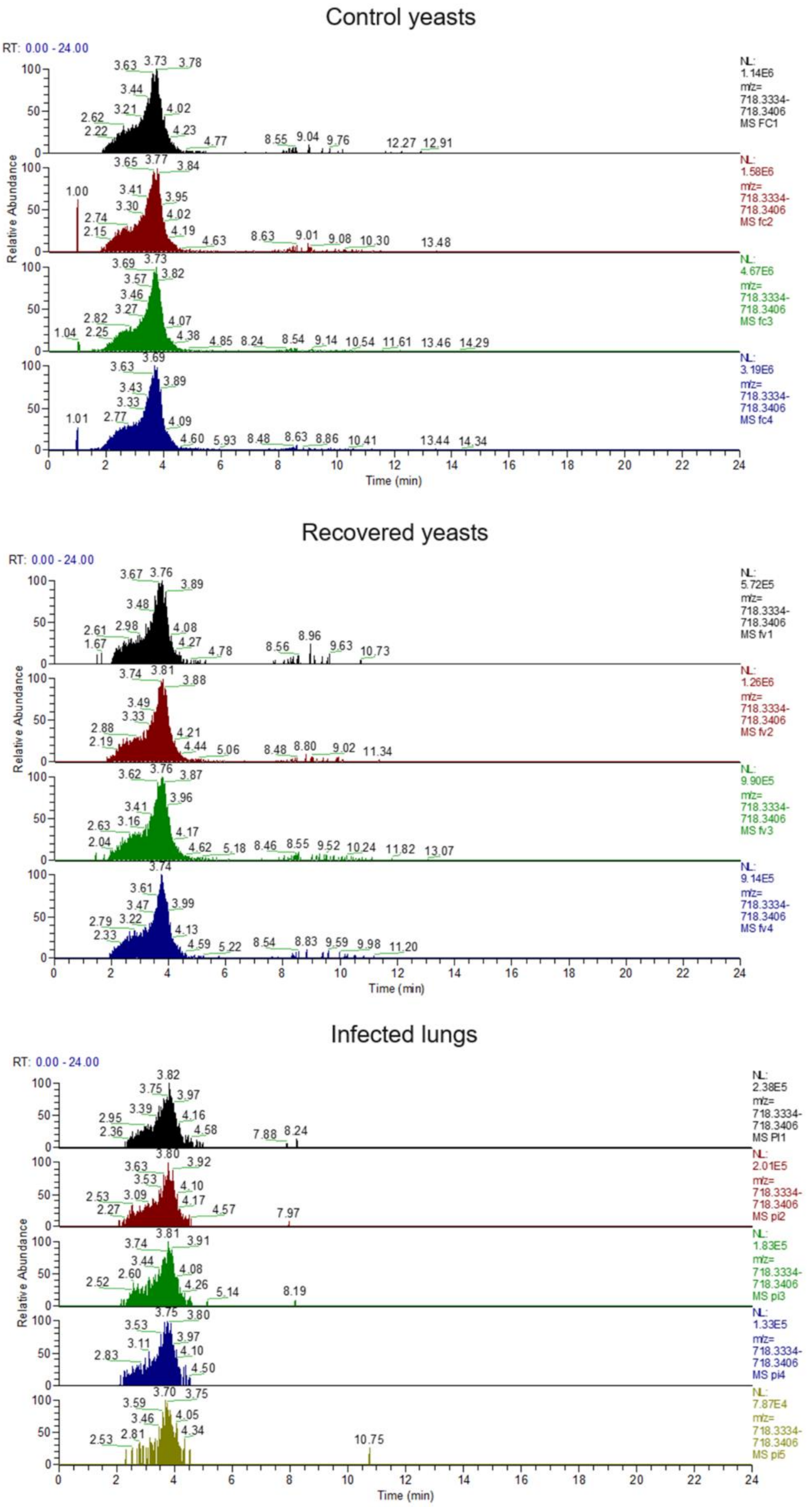

Supplement: Supplementary file 1 [file Presentation_1.pdf]
