# Supplementary material for: Transcriptional profiling of a fungal granuloma reveals a low metabolic activity of Paracoccidioides brasiliensis yeasts and an actively regulated host immune response
Source: Front Cell Infect Microbiol. 2023 Oct 5;13:1268959. doi: 10.3389/fcimb.2023.1268959 (PMC10585178; doi:10.3389/fcimb.2023.1268959)
Supplement: Supplementary file 2 [file Table_1.pdf]

Supplementary Table 1. Genes and primers used for RT-qPCR.

Genes	Forward	Reverse
Mus musculus		
Tnnc1 - troponin C	GGGTGTGGAGTAGATGCTGG	ATTTGCGTTCCCCAGGACAG
Hamp - hepcidin	GAGAGACACCAACTTCCCCA	GTGGGGAGGGCAGGAATAAA
Pdcd1 - programmed cell death 1	CTAGAGCCACCCACACATCC	GAGAGCCAGAACCCAACTCC
Tnf - tumor necrosis factor	CGGGCAGGTCTACTTTGGAG	ACCCTGAGCCATAATCCCCT
Cxcl9 - chemokine (C-X-C motif) ligand 9	AACCTTTGGCCTCCTTGCTT	AGGAAGGAAGGCAGGCAATC
Gbp2b - guanylate binding protein 2b	GTCCTGACTTTGGCCCAGAT	CTCAATGGCCTCCCTCTCAC
Usp9x - Probable ubiquitin carboxyl-terminal hydrolase FAF-X	TGGTTTCATTGTAGGGCCCA	ACCTGAAAGCTTGGAGGACA
Lcn2 - Lipocalin 2	ACTGAATGGGTGGTGAGTGT	GGGAGTGCTGGCCAAATAAG
Paracoccidioides brasiliensis		
PADG_00104 (Oxidoreductase OXR1)	TCGCAGCTGAGTTGAAAATG	CACCCTCCTTCGACTGTGTT
PADG_00097 (L-ornithine N5-oxygenase SidA)	GAGGAGAGCGAGCTATTCCA	ATTTCAACCGCACTCTGACC
PADG_00099 (Acyl-CoA ligase SIDI)	TGGTGATCGAATGGACTTCA	CGTGTAGTGCTGTGCACCTT
PADG_05660 (fungal nitric oxide reductase)	AAAGCAGGGGAAGGCATAAT	CAGTACGAGCCAACCACTCA
PADG_01954 (superoxide dismutase, Fe-Mn family)	AACAAGGCCCTTCAAGATCA	GCCTCAATCTTCGTCTCTGG
PADG_00714 (Pyruvate descarboxylase)	CCGACTTCAACACAACAGGG	TTGCAATTTTCGGGATGGGG
