# Supplementary material for: Transcriptional profiling of a fungal granuloma reveals a low metabolic activity of Paracoccidioides brasiliensis yeasts and an actively regulated host immune response
Source: Front Cell Infect Microbiol. 2023 Oct 5;13:1268959. doi: 10.3389/fcimb.2023.1268959 (PMC10585178; doi:10.3389/fcimb.2023.1268959)
Supplement: Supplementary file 3 [file Table_2.pdf]

Supplementary Table 2. Upregulated mice genes.

Acession number	Protein	Expression status	Log (Fold Change)	Adjusted p-value
PRRs and other receptors				
Clec4a2	C-type lectin domain family 4, member a2	UP(D)	2,118637	1,62673E-05
Clec4n	C-type lectin domain family 6 member A	UP(D)	2,078333	3,77685E-05
Ms4a7	membrane-spanning 4-domains subfamily A member 5/6/7	UP(D)	2,057576	3,89263E-05
Clec7a	C-type lectin domain family 7 member A	UP(D)	2,020814	3,0056E-05
Tlr2	toll-like receptor 2	UP(D)	1,960263	2,91576E-05
Ms4a6d	membrane-spanning 4-domains subfamily A member 5/6/7	UP(D)	1,952377	1,23965E-05
Tlr13	toll-like receptor 13	UP(D)	1,903147	3,02766E-05
Clec4d	C-type lectin domain family 4 member D	UP(D)	1,868473	0,000104122
Ptx3	Pentraxin-related protein PTX3	UP(D)	1,851374	2,5485E-05
Cd14	monocyte differentiation antigen CD14	UP(D)	1,846054	4,36701E-05
Raet1d	retinoic acid early transcript 1	UP(D)	1,757167	1,85594E-05
Clec4e	C-type lectin domain family 4 member E	UP(D)	1,715215	5,88412E-05
Cd68	CD68 antigen	UP(D)	1,338493	0,001331954
Clec5a	C-type lectin domain family 5 member A	UP(D)	1,285129	0,000550618
Marco	macrophage receptor with collagenous structure	UP(12)	1,176102	0,023211938
Clec12a	C-type lectin domain family 12, member a	UP(12)	1,068438	0,000333106
Clec4b1	C-type lectin domain family 4, member b1	UP(D)	1,068106	2,24706E-05
Cell adhesion, activation and differentiation				
Cd52	CDW52 antigen	UP(D)	2,227853	1,29423E-05
Fpr2	formyl peptide receptor-like	UP(D)	2,224199	3,4629E-05
Lgals3	galectin-3	UP(D)	2,113594	2,269E-05
Gpr15	G protein-coupled receptor 15	UP(D)	2,086638	3,18579E-05
Rgs1	regulator of G-protein signaling	UP(D)	2,060517	2,25836E-05
Mzb1	marginal zone B and B1 cell-specific protein 1	UP(D)	2,007265	2,24691E-05
Bst1	Bone marrow stromal cell antigen 1	UP(D)	1,996828	3,23405E-05
Cd300c2	CD300 antigen	UP(D)	1,725508	5,96672E-05
Clmp	adipocyte-specific adhesion molecule	UP(D)	1,678447	5,53956E-05
Retnlg	resistin	UP(D)	1,619956	4,07094E-05
Trem3	Triggering receptor expressed on myeloid cells 3	UP(D)	1,611529	8,33298E-06
Trem1	triggering receptor expressed on myeloid cells 1	UP(D)	1,408704	0,002771287
Chil3	chitinase 3-like 3/4	UP(D)	1,221064	0,000310931
Cd177	CD177 antigen	UP(D)	1,202699	0,000194434
Agr2	anterior gradient protein 2	UP(8)	1,164057	0,00134062
Cdhr1	cadherin-related family member 1	UP(D)	1,14626	1,82092E-05
Chil4	chitinase 3-like 3/4	UP(D)	1,136736	7,62846E-05
S100a9	protein S100-A9	UP(D)	1,127137	2,63106E-05
Tff2	trefoil factor 2	UP(8)	1,113102	0,004094218
S100a8	protein S100-A8	UP(D)	1,10375	0,001126456
Frzb	Secreted frizzled-related protein 3	UP(8)	1,034516	0,002420513
Nuggc	Nuclear GTPase SLIP-GC	UP(D)	1,005003	3,32055E-05
Pro-inflammatory and complement system				
Gbp2b	Guanylate-binding protein 2b	UP(D)	2,872045	4,60703E-05

Camp	cathelicidin antimicrobial peptide	UP(D)	2,319225	0,000175036
Saa3	serum amyloid A protein	UP(D)	2,044481	2,29959E-05
Nos2	nitric-oxide synthase, inducible [EC:1.14.13.39]	UP(D)	2,033614	3,01129E-05
Gbp5	guanylate-binding protein 5	UP(D)	2,027516	1,7783E-05
C1qa	Complement C1q subcomponent subunit A	UP(D)	2,025756	1,63519E-05
Aif1	allograft inflammatory factor 1	UP(D)	2,025499	0,000362794
Cfb	complement factor B [EC:3.4.21.47]	UP(D)	1,935486	2,76615E-05
Tarm1	T-cell-interacting, activating receptor on myeloid cells protein 1	UP(D)	1,931302	3,84086E-05
Cybb	NADPH oxidase 2 [EC:1.-.-.-]	UP(D)	1,775452	6,72975E-05
Pfpl	Macrophage-expressed gene 1 protein	UP(D)	1,758943	4,6842E-05
C1qc	complement C1q subcomponent subunit C	UP(D)	1,729555	0,000173539
Saa1	serum amyloid A protein	UP(D)	1,684414	3,40102E-05
C3ar1	C3a anaphylatoxin chemotactic receptor	UP(D)	1,615371	8,46996E-05
Clca1	Calcium-activated chloride channel regulator 1	UP(8)	1,438976	0,025102437
Pilra	Paired immunoglobulin-like type 2 receptor alpha	UP(D)	1,401804	0,00025608
C1qb	complement C1q subcomponent subunit B	UP(D)	1,346731	0,000137816
Saa2	serum amyloid A protein	UP(D)	1,284467	0,000676768
Cd300ld	CMRF35-like molecule 5	UP(8)	1,049027	7,72548E-07
Pilrb1	Paired immunoglobulin-like type 2 receptor beta-1	UP(12)	1,020848	4,51687E-05
Pilrb2	Paired immunoglobulin-like type 2 receptor beta-2	UP(12)	1,008983	7,17385E-05
Cytokines and chemokines				
Cxcl9	C-X-C motif chemokine 9	UP(D)	2,2741	1,44765E-05
Il1f9	Interleukin-36 gamma	UP(D)	2,253036	1,56045E-05
Ccl20	C-C motif chemokine 20	UP(D)	2,103374	4,42457E-05
Cxcl1	C-X-C motif chemokine 1/2/3	UP(D)	2,073216	0,000421378
Ccl2	C-C motif chemokine 2	UP(D)	2,069786	1,95456E-05
Ccl7	C-C motif chemokine 7	UP(D)	2,057447	2,18951E-05
Cxcl10	C-X-C motif chemokine 10	UP(D)	2,011544	8,14464E-05
Cxcl13	C-X-C motif chemokine 13	UP(D)	1,95105	1,26832E-05
Ccr1	C-C chemokine receptor type 1	UP(D)	1,946485	6,0567E-05
Tnf	tumor necrosis factor superfamily, member 2	UP(D)	1,930536	1,23067E-05
Il1b	interleukin 1 beta	UP(D)	1,895732	7,86476E-05
Ccl8	C-C motif chemokine 8	UP(D)	1,886362	2,85292E-05
Il1a	interleukin 1 alpha	UP(D)	1,850909	7,78257E-06
Spp1	Osteopontin	UP(D)	1,739861	3,3628E-05
Tnfrsf17	tumor necrosis factor receptor superfamily member 17	UP(D)	1,714299	1,64858E-05
Ccl3	C-C motif chemokine 3	UP(D)	1,319954	0,000204104
Tnfrsf9	tumor necrosis factor receptor superfamily member 9	UP(D)	1,266412	3,22183E-05
Cxcl2	C-X-C motif chemokine 1/2/3	UP(D)	1,208573	0,00046053
Il1r2	interleukin 1 receptor type II	UP(D)	1,163824	4,2785E-05
Ccl6	C-C motif chemokine 6	UP(D)	1,161484	1,38757E-05
Cxcl5	C-X-C motif chemokine 5/6	UP(D)	1,114359	2,20334E-05
Ccl12	C-C motif chemokine 2	UP(D)	1,10909	2,27909E-05
Ifng	interferon gamma	UP(8)	1,105272	0,001061971
Ccl4	C-C motif chemokine 4	UP(D)	1,103517	3,92212E-05
Tnfrsf8	Tumor necrosis factor receptor superfamily member 8	UP(8)	1,095759	0,000679234

Cxcr1	C-X-C chemokine receptor type 1	UP(D)	1,081081	0,000307102
Ccl9	C-C motif chemokine 9	UP(D)	1,070775	0,002911195
Ccr5	C-C chemokine receptor type 5	UP(8)	1,066194	0,000570019
Antigen processing and presentation				
H2-M2	Histocompatibility 2, M region locus 2	UP(D)	1,964557	3,04312E-05
Iigp1	Interferon-inducible GTPase 1	UP(D)	1,9352	1,23135E-05
Dcstamp	Dendritic cell-specific transmembrane protein	UP(D)	1,137807	1,51978E-05
Antobody				
Ighv1-47	Immunoglobulin heavy variable 1-47	UP(D)	2,702582	4,18565E-05
Ighv7-4	Immunoglobulin heavy variable 7-4	UP(D)	2,591917	2,20979E-05
Igkv3-11	Immunoglobulin kappa variable 3-11	UP(D)	2,468751	1,398E-05
Ighv5-15	Immunoglobulin heavy variable 5-15	UP(D)	2,463992	4,51892E-05
Ighv1-80	Immunoglobulin heavy variable 1-80	UP(D)	2,452668	4,49753E-05
Ighv1-56	Immunoglobulin heavy variable 1-56	UP(D)	2,417073	2,54059E-05
Ighv7-3	Immunoglobulin heavy variable 7-3	UP(D)	2,400919	5,00508E-05
Ighv6-6	Immunoglobulin heavy variable 6-6	UP(D)	2,373259	0,000175154
Ighv1-26	Immunoglobulin heavy variable 1-26	UP(D)	2,340892	7,64951E-05
Igkv3-7	Immunoglobulin kappa variable 3-7	UP(D)	2,313811	3,14871E-05
Ighv5-17	Immunoglobulin heavy variable 5-17	UP(D)	2,297423	2,41942E-05
Ighv1-85	Immunoglobulin heavy variable 1-85	UP(D)	2,269718	1,21416E-05
Igkv3-8	Immunoglobulin kappa variable 3-8	UP(D)	2,24059	1,79182E-05
Igha	Immunoglobulin heavy constant alpha	UP(D)	2,233271	2,43913E-05
Ighv1-32	Immunoglobulin heavy variable 1-32	UP(D)	2,223415	1,83593E-05
Igkv4-59	Immunoglobulin kappa variable 4-59	UP(D)	2,219996	0,000649463
Ighv1-39	Immunoglobulin heavy variable 1-39	UP(D)	2,219915	3,9045E-05
Ighv5-4	Immunoglobulin heavy variable 5-4	UP(D)	2,218446	0,000354697
Ighv1-37	Immunoglobulin heavy variable 1-37	UP(D)	2,20106	4,26797E-05
Igkv13-84	Immunoglobulin kappa chain variable 13-84	UP(D)	2,19989	4,72581E-05
Ighv2-6	Immunoglobulin heavy variable 2-6	UP(D)	2,183847	3,71037E-05
Igkv4-80	Immunoglobulin kappa variable 4-80	UP(D)	2,175198	2,24461E-05
Ighv9-4	Immunoglobulin heavy variable 9-4	UP(D)	2,168834	1,40392E-05
Igkv3-4	Immunoglobulin kappa variable 3-4	UP(D)	2,164317	3,63617E-05
Igkv10-95	Immunoglobulin kappa variable 10-95	UP(D)	2,164143	1,56062E-05
Ighv2-4	Immunoglobulin heavy variable V2-4	UP(D)	2,152911	1,22629E-05
Igkv3-2	Immunoglobulin kappa variable 3-2	UP(D)	2,143267	1,39763E-05
Ighv10-1	Immunoglobulin heavy variable 10-1	UP(D)	2,139018	1,79674E-05
Igkv3-1	Immunoglobulin kappa variable 3-1	UP(D)	2,137117	1,11766E-05
Ighv1-43	Immunoglobulin heavy variable V1-43	UP(D)	2,133713	2,27735E-05
Igkv10-96	Immunoglobulin kappa variable 10-96	UP(D)	2,123301	2,85705E-05
Ighv1-69	Immunoglobulin heavy variable 1-69	UP(D)	2,111835	4,70575E-05
Ighv8-14	Immunoglobulin heavy variable 8-13	UP(D)	2,11051	0,000108458
Ighv1-4	Immunoglobulin heavy variable 1-4	UP(D)	2,110384	2,67013E-05
Ighv10-3	Immunoglobulin heavy variable V10-3	UP(D)	2,109727	0,000103493
Igkv6-29	Immunoglobulin kappa chain variable 6-29	UP(D)	2,108656	2,1717E-05
Igkv14-100	Immunoglobulin kappa chain variable 14-100	UP(D)	2,103544	1,72872E-05
Ighv1-62-3	Ig heavy chain V region 1-62-3	UP(D)	2,101752	0,000178754

Igkv6-20	Immunoglobulin kappa variable 6-20	UP(D)	2,100655	1,90792E-05
Igkv5-39	Immunoglobulin kappa variable 5-39	UP(D)	2,09464	4,91528E-05
Ighv1-74	Immunoglobulin heavy variable V1-74	UP(D)	2,094476	2,07072E-05
Ighv1-77	Immunoglobulin heavy variable 1-77	UP(D)	2,086679	1,72005E-05
Ighv1-82	Immunoglobulin heavy variable 1-82	UP(D)	2,086285	1,61842E-05
Igkv13-85	Immunoglobulin kappa chain variable 13-85	UP(D)	2,083985	1,45588E-05
Igkv5-48	Ig kappa chain V-V region L7	UP(D)	2,083877	4,73861E-05
Igkv1-115	Immunoglobulin kappa variable 1-99	UP(D)	2,080784	2,13978E-05
Ighv6-5	Immunoglobulin heavy variable V6-5	UP(D)	2,078298	3,25211E-05
Ighv1-52	Immunoglobulin heavy variable 1-52	UP(D)	2,075601	1,85229E-05
Ighv1-70	Immunoglobulin heavy variable 1-70	UP(D)	2,075272	4,70942E-05
Igkv4-92	Immunoglobulin kappa variable 4-92	UP(D)	2,072162	1,60065E-05
Igkv4-62	Immunoglobulin kappa variable 4-62	UP(D)	2,069767	1,29002E-05
Igkv5-40-1	Immunoglobulin kappa variable 5-40-1	UP(D)	2,068266	8,08931E-05
Ighv6-7	Immunoglobulin heavy variable V6-7	UP(D)	2,063071	4,69098E-05
Ighv5-1	Immunoglobulin heavy variable 5-1	UP(D)	2,061682	7,41565E-06
Ighv1-55	Immunoglobulin heavy variable 1-55	UP(D)	2,05409	0,000263588
Ighv1-66	Immunoglobulin heavy variable 1-66	UP(D)	2,053211	2,85323E-05
Ighv14-4	Immunoglobulin heavy variable 14-4	UP(D)	2,05137	3,78784E-05
Igkv8-34	Immunoglobulin kappa variable 8-34	UP(D)	2,047021	2,91859E-05
Ighv1-84	Immunoglobulin heavy variable 1-84	UP(D)	2,044093	1,69041E-05
Igkv4-50	Immunoglobulin kappa variable 4-50	UP(D)	2,033523	2,34426E-05
Ighv1-33	Immunoglobulin heavy variable 1-33	UP(D)	2,029047	8,40413E-05
Igkv9-123	Immunoglobulin kappa variable 9-123	UP(D)	2,025056	3,14152E-05
Ighv2-2	Immunoglobulin heavy variable 2-2	UP(D)	2,024781	1,2994E-05
Igkv5-43	Immunoglobulin kappa chain variable 5-43	UP(D)	2,023984	2,62565E-05
Ighv3-7	Immunoglobulin heavy variable 3-7	UP(D)	2,017776	0,000104341
Ighv1-22	Immunoglobulin heavy variable 1-22	UP(D)	2,007573	1,24414E-05
Ighv2-5	Immunoglobulin heavy variable 2-5	UP(D)	2,003811	1,24937E-05
Ighv1-21-1	Immunoglobulin heavy variable 1-21-1	UP(D)	2,002292	5,28196E-05
Ighv1-36	Immunoglobulin heavy variable 1-36	UP(D)	2,001255	4,62177E-05
Ighv1-25	Immunoglobulin kappa variable 1-25	UP(D)	1,999181	1,21249E-05
Ighv1-59	Immunoglobulin heavy variable V1-59	UP(D)	1,998215	3,89883E-05
Ighv1-76	Immunoglobulin heavy variable 1-76	UP(D)	1,997389	5,44329E-05
Ighv14-2	Immunoglobulin heavy variable 14-2	UP(D)	1,996771	6,17544E-05
Ighv1-12	Immunoglobulin heavy variable V1-12	UP(D)	1,995246	3,33483E-05
Igkv17-134	Immunoglobulin kappa variable 17-134	UP(D)	1,993742	4,61496E-05
Igkv4-79	Immunoglobulin kappa variable 4-79	UP(D)	1,99112	1,11578E-05
Igkv19-93	Immunoglobulin kappa chain variable 19-93	UP(D)	1,990508	2,55746E-05
Igkv3-5	Immunoglobulin kappa chain variable 3-5	UP(D)	1,986897	2,08651E-05
Ighv5-16	Immunoglobulin heavy variable 5-16	UP(D)	1,986013	0,000490023
Igkv4-58	Immunoglobulin kappa variable 4-58	UP(D)	1,984759	4,53065E-05
Igkv5-45	Immunoglobulin kappa chain variable 5-45	UP(D)	1,979461	4,72959E-05
Igkv1-117	Immunoglobulin kappa variable 1-117	UP(D)	1,976239	4,62935E-05
Igkv1-108	Immunoglobulin kappa variable 1-108	UP(D)	1,976095	4,54967E-05
Ighv15-2	Immunoglobulin heavy variable V15-2	UP(D)	1,972091	2,79147E-05

Ighv7-2	Immunoglobulin heavy variable 7-2	UP(D)	1,969074	2,96333E-05
Ighv9-3	Immunoglobulin heavy variable V9-3	UP(D)	1,968117	1,58436E-05
Igkv4-65	Immunoglobulin kappa chain variable 4-65	UP(D)	1,96693	3,70924E-05
Ighv1-18	Immunoglobulin heavy variable V1-18	UP(D)	1,964458	7,66873E-05
Ighv3-6	Ig heavy chain V region 3-6	UP(D)	1,960099	3,81438E-05
Ighv3-8	Immunoglobulin heavy variable V3-8	UP(D)	1,958354	0,000132198
Igkv8-28	Immunoglobulin kappa variable 8-28	UP(D)	1,955236	0,000102195
Ighv1-75	Immunoglobulin heavy variable 1-75	UP(D)	1,95061	7,82712E-05
Ighv1-17	Immunoglobulin heavy variable 1-17	UP(D)	1,948285	1,32175E-05
Igkv12-41	Immunoglobulin heavy variable 12-41	UP(D)	1,947874	2,27296E-05
Igkv8-19	Immunoglobulin kappa variable 8-19	UP(D)	1,946527	2,00559E-05
Igkv12-46	Immunoglobulin kappa variable 12-46	UP(D)	1,943494	7,86856E-05
Igkv4-54	Immunoglobulin kappa chain variable 4-54	UP(D)	1,939039	3,78941E-05
Igkv12-40	Immunoglobulin heavy variable 12-40	UP(D)	1,936221	0,000165339
Igkv12-44	Immunoglobulin kappa variable 12-44	UP(D)	1,934594	5,06264E-05
Ighv1-50	Immunoglobulin heavy variable 1-50	UP(D)	1,932819	0,000159461
Ighv1-2	Immunoglobulin heavy variable 1-2	UP(D)	1,928345	6,33521E-05
Ighv1-61	Ig heavy chain V region 3	UP(D)	1,928332	2,27256E-05
Ighv5-6	Immunoglobulin heavy variable 5-6	UP(D)	1,92394	2,24444E-05
Igkc	Immunoglobulin kappa constant	UP(D)	1,918386	7,82879E-05
Igkv3-12	Immunoglobulin kappa variable 3-12	UP(D)	1,917397	1,72005E-05
Ighv9-2	Immunoglobulin heavy variable 9-2	UP(D)	1,905901	1,23135E-05
Igkv9-124	Immunoglobulin kappa chain variable 9-124	UP(D)	1,898092	8,20352E-05
Ighv1-27	Immunoglobulin heavy variable 1-27	UP(D)	1,891259	0,000155784
Ighv14-1	Immunoglobulin heavy variable 14-1	UP(D)	1,881154	1,94369E-05
Ighv1-31	Immunoglobulin heavy variable 1-31	UP(D)	1,879359	5,74218E-05
Ighv1-38	Probable non-functional immunoglobulin heavy variable 1-38-4	UP(D)	1,876343	2,84354E-05
Igkv3-9	Immunoglobulin kappa variable 3-9	UP(D)	1,875846	6,20453E-05
Igkv4-57	Immunoglobulin kappa variable 4-57	UP(D)	1,874248	2,77027E-05
Iglc1	Immunoglobulin lambda constant 1	UP(D)	1,868199	0,000195941
Igkv8-27	Immunoglobulin kappa chain variable 8-27	UP(D)	1,866328	4,08991E-05
Igkv1-110	Immunoglobulin kappa variable 1-110	UP(D)	1,853703	2,1549E-05
Igkv5-37	Immunoglobulin kappa variable 5-37	UP(D)	1,853429	5,00365E-05
Igkv3-10	Immunoglobulin kappa variable 3-10	UP(D)	1,853411	2,46049E-05
Igkv17-127	Immunoglobulin kappa variable 17-127	UP(D)	1,847779	2,06242E-05
Ighv5-9	Immunoglobulin heavy variable 5-9	UP(D)	1,844385	7,92255E-06
Igkv6-17	Immunoglobulin kappa variable 6-17	UP(D)	1,840901	3,41047E-05
Ighv1-21	Immunoglobulin heavy variable 1-21-1	UP(D)	1,838058	8,44706E-05
Ighv1-81	Immunoglobulin heavy variable 1-81	UP(D)	1,832813	1,33484E-05
Ighv1-78	Immunoglobulin heavy variable 1-78	UP(D)	1,82919	4,12158E-05
Igkv9-120	Ig kappa chain V-V region MOPC 41	UP(D)	1,827616	4,15864E-05
Ighv14-3	Immunoglobulin heavy variable V14-3	UP(D)	1,825134	4,22443E-05
Ighv1-34	Immunoglobulin heavy variable 1-34	UP(D)	1,824994	0,000170463
Ighv2-6-8	Immunoglobulin heavy variable 2-6-8	UP(D)	1,823368	1,19409E-05
Iglc4	Immunoglobulin lambda constant 4	UP(D)	1,814971	1,12931E-05
Igkv8-26	Immunoglobulin kappa variable 8-26	UP(D)	1,814224	3,9734E-05

Ighv1-30	Immunoglobulin heavy variable 1-30	UP(D)	1,808748	0,001250152
Ighv1-62	Ig heavy chain V region 1-62-3	UP(D)	1,792005	2,75376E-05
Igkv4-57-1	Immunoglobulin kappa variable 4-57-1	UP(D)	1,789106	1,35837E-05
Igkv4-81	Immunoglobulin kappa variable 4-81	UP(D)	1,78826	0,000231116
Ighv1-9	Immunoglobulin heavy variable V1-9	UP(D)	1,774702	0,000134759
Igkv4-78	Immunoglobulin kappa variable 4-78	UP(D)	1,769766	5,69201E-05
Ighv1-19	Immunoglobulin heavy variable V1-19	UP(D)	1,769326	2,0637E-05
Igkv17-121	Immunoglobulin kappa variable 17-121	UP(D)	1,752632	0,000171027
Ighv1-15	Immunoglobulin heavy variable 1-15	UP(D)	1,744905	0,004259293
Igkv8-21	Immunoglobulin kappa variable 8-21	UP(D)	1,735988	9,63148E-05
Ighv2-3	Immunoglobulin heavy variable 2-3	UP(D)	1,721498	1,44164E-05
Ighv5-12	Immunoglobulin heavy variable 5-12	UP(D)	1,713431	3,44746E-05
Igkv3-3	Immunoglobulin kappa variable 3-3	UP(D)	1,683577	0,000101717
Ighv8-8	Immunoglobulin heavy variable 8-8	UP(D)	1,646881	4,6526E-05
Ighv1-7	Immunoglobulin heavy variable V1-7	UP(D)	1,638951	6,0072E-05
Igkv6-32	Immunoglobulin kappa variable 6-32	UP(D)	1,637352	2,01761E-05
Ighv1-28	Immunoglobulin heavy variable 1-28	UP(D)	1,637233	1,33484E-05
Ighv2-9	Immunoglobulin heavy variable 2-9	UP(D)	1,630883	2,24268E-05
Ighv1-5	Immunoglobulin heavy variable V1-5	UP(D)	1,62865	0,000229915
Igkv4-55	Immunoglobulin kappa variable 4-55	UP(D)	1,627099	0,000442916
Igkv4-90	Immunoglobulin kappa chain variable 4-90	UP(D)	1,627008	0,00010704
Ighv1-86	Immunoglobulin kappa variable 1-86	UP(D)	1,616125	9,02684E-05
Igkj5	Immunoglobulin kappa joining 5	UP(D)	1,60611	4,93765E-05
Ighv1-63	Immunoglobulin heavy variable V1-63	UP(D)	1,600555	0,000286222
Igkj1	Immunoglobulin kappa joining 1	UP(D)	1,595506	7,70735E-05
Igsf6	NA	UP(D)	1,594943	0,000182565
Ighv1-64	Immunoglobulin heavy variable 1-64	UP(D)	1,594637	0,001016654
Igkv4-68	Immunoglobulin kappa variable 4-68	UP(D)	1,594111	3,03335E-05
Igkv2-116	Immunoglobulin heavy variable 2-116	UP(D)	1,584057	1,37053E-05
Ighv8-13	Immunoglobulin heavy variable 8-13	UP(D)	1,58303	1,50499E-05
Ighv5-9-1	Immunoglobulin heavy variable 5-9-1	UP(D)	1,57329	1,23135E-05
Ighv1-73	Immunoglobulin heavy variable 1-73	UP(D)	1,56084	5,68825E-05
Ighv1-83	Immunoglobulin heavy variable 1-83	UP(D)	1,554823	0,000357687
Ighv1-71	Immunoglobulin heavy variable 1-71	UP(D)	1,550219	0,000124902
Ighv1-20	Immunoglobulin heavy variable V1-20	UP(D)	1,549851	7,41565E-06
Igkv10-94	Immunoglobulin kappa variable 10-94	UP(D)	1,528881	3,10885E-05
Igkv15-103	Immunoglobulin kappa chain variable 15-103	UP(D)	1,520912	3,07306E-05
Igkv4-74	Immunoglobulin kappa variable 4-74	UP(D)	1,519513	0,010136701
Ighv8-9	Immunoglobulin heavy variable V8-9	UP(D)	1,509825	0,00029336
Igkv4-77	Immunoglobulin kappa variable 4-77	UP(D)	1,505421	6,80698E-05
Ighv4-1	Immunoglobulin heavy variable 4-1	UP(D)	1,504017	0,00017205
Igkv14-126	Immunoglobulin kappa variable 14-126	UP(D)	1,493909	0,001119296
Igkv1-122	Immunoglobulin kappa chain variable 1-122	UP(D)	1,489952	3,9252E-05
Ighv2-9-1	Immunoglobulin heavy variable 2-9-1	UP(D)	1,486335	0,000131473
Ighv6-3	Immunoglobulin heavy variable 6-3	UP(D)	1,480372	2,30539E-05
Igkv1-35	Immunoglobulin kappa variable 1-35	UP(D)	1,437979	4,84414E-05

Ighv1-53	Immunoglobulin heavy variable 1-53	UP(D)	1,435702	2,9713E-05
Iglv2	Immunoglobulin lambda variable 2	UP(D)	1,435017	3,88761E-06
Igkv4-63	Immunoglobulin kappa variable 4-63	UP(D)	1,424475	0,003100021
Igkv4-72	Immunoglobulin kappa chain variable 4-72	UP(D)	1,420407	0,002554132
Igkv1-132	Immunoglobulin kappa variable 1-132	UP(D)	1,401539	1,28962E-05
Igkv1-133	Immunoglobulin kappa variable 1-133	UP(D)	1,353419	5,57687E-05
Ighv1-67	Immunoglobulin heavy variable V1-67	UP(D)	1,353262	1,24812E-05
Ighv1-19-1	Immunoglobulin heavy variable 1-19-1	UP(D)	1,35123	2,85368E-05
Ighv8-12	Immunoglobulin heavy variable V8-12	UP(D)	1,346521	4,66171E-05
Igkv4-71	Immunoglobulin kappa chain variable 4-71	UP(D)	1,320406	0,000253842
Ighg3	Ig gamma-3 chain C region	UP(D)	1,320073	0,001385028
Igkv8-31	Immunoglobulin kappa variable 8-31	UP(D)	1,318256	4,89248E-05
Igkv8-16	immunoglobulin kappa variable 8-16	UP(D)	1,315466	0,000793119
Igkv4-53	Immunoglobulin kappa variable 4-53	UP(D)	1,298448	0,000254865
Igkv6-14	Immunoglobulin kappa variable 6-14	UP(D)	1,29698	8,60564E-06
Ighv1-42	Immunoglobulin heavy variable V1-42	UP(D)	1,295163	0,000187251
Ighv6-4	Immunoglobulin heavy variable V6-4	UP(D)	1,294585	0,00022562
Igkv4-60	Immunoglobulin kappa variable 4-60	UP(D)	1,286708	3,88147E-05
Igkv4-86	Immunoglobulin kappa variable 4-86	UP(D)	1,276338	3,09341E-05
Igkv4-70	Immunoglobulin kappa chain variable 4-70	UP(D)	1,272792	0,001887071
Ighv1-62-1	Immunoglobulin heavy variable 1-62-1	UP(D)	1,259148	0,000556684
Ighe	Ig epsilon chain C region	UP(D)	1,248241	7,7328E-05
Igkv14-130	Immunoglobulin kappa variable 14-130	UP(D)	1,248088	9,72305E-05
Igkv2-112	Immunoglobulin kappa variable 2-112	UP(D)	1,242291	0,000290523
Iglv1	Immunoglobulin lambda chain variable region	UP(D)	1,242272	0,000258841
Ighv1-79	Immunoglobulin kappa variable 1-79	UP(D)	1,238163	3,03474E-05
Igkv6-15	Immunoglobulin kappa variable 6-15	UP(D)	1,236529	0,000435612
Igkv4-91	Immunoglobulin kappa chain variable 4-91	UP(D)	1,222303	0,000560744
Igkv8-23-1	immunoglobulin kappa variable 8-23-1	UP(D)	1,202952	0,000620013
Ighv1-72	Ig heavy chain V region 1-72	UP(D)	1,198094	9,28962E-06
Igkv12-89	Immunoglobulin kappa chain variable 12-89	UP(D)	1,187129	0,000274891
Ighv1-62-2	Immunoglobulin heavy variable 1-62-2	UP(D)	1,182234	2,64442E-05
Igkv16-104	Immunoglobulin kappa variable 16-104	UP(D)	1,182065	0,000133636
Ighv4-2	IgM heavy chain VDJ region	UP(D)	1,179356	9,42265E-05
Ighv9-1	Immunoglobulin heavy variable 9-1	UP(D)	1,170058	0,000128823
Igkv12-38	Immunoglobulin kappa chain variable 12-38	UP(D)	1,164827	0,000325246
Igkv4-73	Immunoglobulin kappa variable 4-74	UP(D)	1,162281	4,36545E-05
Igkv1-131	Immunoglobulin kappa variable 1-131	UP(D)	1,155763	2,18869E-05
Igkv1-135	Immunoglobulin kappa variable 1-135	UP(D)	1,155126	0,000543733
Igkv3-6	Immunoglobulin kappa variable 3-6	UP(D)	1,152119	0,000145004
Ighv5-12-4	Immunoglobulin heavy variable 5-12-4	UP(D)	1,145454	0,000112351
Igkv8-24	Immunoglobulin kappa chain variable 8-24	UP(D)	1,139751	0,000158026
Ighv8-6	Immunoglobulin heavy variable V8-6	UP(D)	1,138856	9,67405E-05
Igkv1-99	Immunoglobulin kappa variable 1-99	UP(D)	1,133169	0,000183584
Igkv12-98	Immunoglobulin kappa variable 12-98	UP(D)	1,1315	0,00036701
Ighg2c	Immunoglobulin heavy constant gamma 2C	UP(D)	1,126418	3,326E-05

Igkv6-25	Immunoglobulin kappa chain variable 6-25	UP(D)	1,125962	1,70875E-05
Igkv1-88	Immunoglobulin kappa chain variable 1-88	UP(D)	1,12578	0,000117409
Igkv6-13	Immunoglobulin kappa variable 6-13	UP(D)	1,12541	0,000919361
Ighv1-58	Immunoglobulin heavy variable 1-58	UP(D)	1,112912	0,007632438
Igkv14-111	Immunoglobulin kappa variable 14-111	UP(D)	1,112462	0,000110122
Igkv4-61	Immunoglobulin kappa chain variable 4-61	UP(D)	1,11134	4,24284E-05
Jchain	Immunoglobulin J chain	UP(D)	1,104429	8,09145E-05
Igkv6-23	Immunoglobulin kappa variable 6-23	UP(D)	1,103654	5,39957E-05
Ighv8-11	Immunoglobulin heavy variable V8-11	UP(D)	1,098955	0,000494422
Igkv8-30	Immunoglobulin kappa chain variable 8-30	UP(D)	1,098602	2,46739E-05
Igkv4-56	Immunoglobulin kappa variable 4-56	UP(D)	1,095479	0,000460964
Ighv8-5	Immunoglobulin heavy variable V8-5	UP(D)	1,094603	1,32528E-05
Igkv12-47	immunoglobulin kappa variable 12-47	UP(12)	1,091345	0,003860936
Ighv1-54	Immunoglobulin heavy variable V1-54	UP(D)	1,090311	1,52227E-05
Ighg2b	Ig gamma-2B chain C region	UP(D)	1,088386	4,8036E-05
Igkv2-137	Immunoglobulin kappa chain variable 2-137	UP(D)	1,082944	0,000276367
Iglj2	Immunoglobulin lambda joining 2	UP(8)	1,080354	0,000365055
Ighv1-14	Immunoglobulin kappa variable 1-14	UP(D)	1,070701	0,003787653
Ighv3-2	Immunoglobulin heavy variable 3-2	UP(D)	1,068828	0,015427685
Ighg1	Ig gamma-1 chain C region secreted form	UP(D)	1,064601	0,000375409
Igkj2	Immunoglobulin kappa joining 2	UP(12)	1,053354	0,000334865
Ighv8-4	immunoglobulin heavy variable V8-4	UP(D)	1,050004	0,000112387
Ighj3	Immunoglobulin heavy joining 3	UP(12)	1,049851	0,000203107
Igkv4-69	Immunoglobulin kappa variable 4-69	UP(D)	1,041042	0,001085013
Igkv4-51	Immunoglobulin kappa chain variable 4-51	UP(D)	1,031409	0,000479724
Ighv3-1	Immunoglobulin heavy variable 3-1	UP(D)	1,028226	0,001072377
Igkv9-129	Immunoglobulin kappa variable 9-129	UP(D)	1,019466	7,50375E-05
Igkv2-109	Immunoglobulin kappa variable 2-109	UP(D)	1,017896	4,2895E-05
Igkj4	Immunoglobulin kappa joining 4	UP(12)	1,01743	0,000135141
Ighv7-1	Immunoglobulin heavy variable 7-1	UP(12)	1,003221	0,001403375
Regulation and tissue repair				
Tyrobp	TYRO protein tyrosine kinase-binding protein	UP(D)	2,364903	5,81443E-05
Slamf7	SLAM family member 7	UP(D)	2,225496	3,21326E-05
Aoah	acyloxyacyl hydrolase [EC:3.1.1.77]	UP(D)	2,170462	1,28695E-05
Fcer1g	high affinity immunoglobulin epsilon receptor subunit gamma	UP(D)	2,149737	0,000193335
Fcgr2b	low affinity immunoglobulin gamma Fc receptor II-b	UP(D)	2,132235	2,90635E-05
Pdcd1	programmed cell death protein 1	UP(D)	2,073949	4,75262E-05
Arg1	Arginase-1	UP(D)	2,059942	4,73352E-05
Chl1	L1 cell adhesion molecule like protein	UP(D)	2,04095	1,41571E-05
Cd200r2	cell surface glycoprotein CD200 receptor	UP(D)	1,917203	1,22629E-05
Lair1	leukocyte-associated Ig-like receptor	UP(D)	1,909598	0,00012657
Wfdc21	Protein Wfdc21	UP(D)	1,861915	2,16746E-05
Fcgr3	low affinity immunoglobulin gamma Fc receptor II-c	UP(D)	1,841431	9,64472E-05
Lilrb4a	leukocyte immunoglobulin-like receptor	UP(D)	1,83873	3,73375E-05
Cd300lf	CO300B/D/F antigen	UP(D)	1,831814	5,77154E-05
Ass1	argininosuccinate synthase [EC:6.3.4.5]	UP(D)	1,798539	4,13462E-05

Cd200r1	cell surface glycoprotein CD200 receptor	UP(D)	1,777666	8,96099E-05
Trem2	triggering receptor expressed on myeloid cells 2	UP(D)	1,756511	6,88102E-05
Mmp8	matrix metalloproteinase-8 (neutrophil collagenase) [EC:3.4.24.34]	UP(D)	1,716975	2,99437E-05
Cd200r4	cell surface glycoprotein CD200 receptor	UP(D)	1,706413	3,65722E-05
Slamf8	SLAM family member 8	UP(D)	1,638288	3,24072E-05
Fcgr4	low affinity immunoglobulin gamma Fc receptor III	UP(D)	1,597777	9,38255E-05
Acod1	aconitate decarboxylase [EC:4.1.1.6]	UP(D)	1,574847	3,6761E-05
Mmp12	matrix metalloproteinase-12 (macrophage elastase) [EC:3.4.24.65]	UP(D)	1,548912	8,80359E-05
Mmp13	Collagenase 3	UP(D)	1,522556	2,11587E-05
Tnip3	TNFAIP3-interacting protein 3	UP(D)	1,423849	7,86978E-05
Mmp3	matrix metalloproteinase-3 (stromelysin 1, progelatinase) [EC:3.4.24.17]	UP(D)	1,40916	0,000232178
Ctla4	cytotoxic T-lymphocyte-associated protein 4	UP(D)	1,368578	0,00016498
Mmp10	matrix metalloproteinase-10 (stromelysin 2) [EC:3.4.24.22]	UP(D)	1,248864	0,00013263
Il1rn	interleukin 1 receptor antagonist	UP(D)	1,244346	0,000408599
Slpi	antileukoproteinase	UP(D)	1,185214	0,000254756
Col24a1	collagen type V/XI/XXIV/XXVII, alpha	UP(D)	1,169066	2,24721E-05
Reg3g	regenerating islet-derived 3 gamma	UP(8)	1,167323	0,000307617
Spsb4	SPRY domain-containing SOCS box protein 1/4	UP(8)	1,162106	0,000143615
Hmox1	heme oxygenase 1 [EC:1.14.14.18]	UP(D)	1,12594	1,22926E-05
A2m	alpha-2-macroglobulin	UP(D)	1,123508	9,21566E-05
Fcrlb	NA	UP(D)	1,121208	2,29193E-05
Retnla	Resistin-like alpha	UP(D)	1,108239	0,005687287
Ereg	epiregulin	UP(D)	1,105559	0,000168334
Btnl6	butyrophilin 6	UP(D)	1,093474	0,000874956
Tigit	T-cell immunoreceptor with Ig and ITIM domains	UP(8)	1,070677	0,00023369
Fgg	Fibrinogen gamma chain	UP(12)	1,038051	0,000425448
Ly6k	lymphocyte antigen 6 complex, locus K	UP(12)	1,032531	0,009785454
Cd274	Programmed cell death 1 ligand 1	UP(8)	1,030962	4,09815E-06
Ly6i	Lymphocyte antigen 6I	UP(12)	1,009717	5,99584E-05
Pdcd1lg2	Programmed cell death 1 ligand 2	UP(8)	1,009324	3,02313E-05
Smpdl3b	Acid sphingomyelinase-like phosphodiesterase 3b	UP(8)	1,007903	1,46933E-05
Fcgr1	High affinity immunoglobulin gamma Fc receptor I	UP(8)	1,004063	2,14204E-05
Gene/protein regulation				
Derl3	Derlin-2/3	UP(D)	2,25272	1,31629E-05
Ovol3	Putative transcription factor ovo-like protein 3	UP(D)	2,234413	1,60519E-05
Npl	Nuclear protein localization protein 4 homolog	UP(D)	2,165087	0,000541129
Ubd	ubiquitin D	UP(D)	2,155088	2,79661E-05
Fut1	galactoside 2-L-fucosyltransferase 1/2 [EC:2.4.1.69]	UP(D)	2,143103	1,51975E-05
Serpina3f	Serine protease inhibitor A3F	UP(D)	2,06118	7,26077E-05
Rnf149	E3 ubiquitin-protein ligase RNF149 [EC:2.3.2.27]	UP(D)	2,032577	1,24636E-05
Rnf128	E3 ubiquitin-protein ligase RNF128 [EC:2.3.2.27]	UP(D)	1,983871	0,000121657
Sdf2l1	Stromal cell-derived factor 2-like protein 1	UP(D)	1,969222	0,000168545
Timp1	metalloproteinase inhibitor 1	UP(D)	1,953594	1,26771E-05
P4ha3	prolyl 4-hydroxylase [EC:1.14.11.2]	UP(D)	1,878234	3,17217E-05

Ear6	Ear6 protein	UP(D)	1,842501	1,2687E-05
Cstb	cystatin-A/B	UP(D)	1,830246	1,44391E-05
Cstdc4	Cystatin domain-containing 4	UP(D)	1,791522	2,80586E-05
Serpina3g	Serine protease inhibitor A3G	UP(D)	1,695611	0,001231103
Serpina3i	Serine (or cysteine) peptidase inhibitor, clade A, member 3I	UP(D)	1,521036	0,000322051
Ctss	cathepsin S [EC:3.4.22.27]	UP(D)	1,520351	3,51307E-05
Spic	transcription factor Spi-C	UP(D)	1,402411	3,84526E-05
Stfa2l1	stefin A2 like 1	UP(D)	1,390768	0,000282332
Rnase2a	Ribonuclease, RNase A family, 2A (liver, eosinophil-derived neurotoxin)	UP(8)	1,279519	0,006481001
Tfec	transcription factor EC	UP(D)	1,269696	3,74224E-05
Wfdc17	Activated macrophage/microglia WAP domain protein	UP(D)	1,26686	0,000203263
Stfa2	cystatin-A/B	UP(D)	1,205505	4,08492E-05
B230303A05Rik	U1 small nuclear ribonucleoprotein 1C pseudogene	UP(D)	1,184436	2,16408E-05
Aicda	Single-stranded DNA cytosine deaminase	UP(D)	1,112878	0,00019665
Csta2	cystatin-A/B	UP(D)	1,088477	0,000269932
Cpa6	Carboxypeptidase A6	UP(8)	1,08247	0,008522467
Serpina3h	Serine protease inhibitor A3H	UP(D)	1,082125	0,000452751
Ctsk	cathepsin K [EC:3.4.22.38]	UP(D)	1,064556	3,06389E-05
Eaf2	ELL-associated factor 2	UP(12)	1,059271	6,37398E-05
B3galt5	beta-1,3-galactosyltransferase 5 [EC:2.4.1.-]	UP(D)	1,038163	0,02047854
Arnt2		UP(8)	1,037611	0,00031628
Basp1	Brain acid soluble protein 1 ·	UP(8)	1,036079	3,40934E-07
Gfi1	Growth factor independent protein 1	UP(D)	1,034997	0,00013217
Cpxm1	Probable carboxypeptidase X1	UP(8)	1,03199	6,67821E-06
Ctsl	Procathepsin L	UP(8)	1,011147	0,000106147
Fcor	Foxo1-corepressor	UP(8)	1,004627	4,09815E-06
Energy and lipid metabolism				
Pla2g7	platelet-activating factor acetylhydrolase [EC:3.1.1.47]	UP(D)	2,167659	0,000342664
Hk3	hexokinase [EC:2.7.1.1]	UP(D)	1,75556	3,40112E-05
Gla	Growth factor independent protein 1	UP(D)	1,303367	0,001139193
Pla1a	phosphatidylserine sn-1 acylhydrolase [EC:3.1.1.111]	UP(D)	1,285157	1,12799E-05
Olr1	oxidised low-density lipoprotein receptor 1	UP(8)	1,201657	1,92057E-06
Lpcat2	lysophosphatidylcholine acyltransferase / lyso-PAF acetyltransferase [EC:2.3.1.23 2.3.1.67]	UP(D)	1,182206	0,000256472
Msr1	macrophage scavenger receptor 1	UP(D)	1,129434	5,68353E-05
Cyp7b1	Cytochrome P450 7B1	UP(12)	1,000947	1,8032E-05
Cell cycle				
Zbp1	Z-DNA-binding protein 1	UP(D)	2,095708	1,45709E-05
Bcl2a1d	hematopoietic Bcl-2-related protein A1	UP(D)	1,946559	1,98711E-05
Inhba	inhibin beta A chain	UP(D)	1,914957	3,05306E-05
Bcl2a1c	hematopoietic Bcl-2-related protein A1	UP(D)	1,878795	2,2889E-05
Mefv	pyrin	UP(D)	1,869159	7,69807E-05
Arl11	ADP-ribosylation factor-like protein 11	UP(D)	1,822941	5,14453E-05
Bcl2a1a	hematopoietic Bcl-2-related protein A1	UP(D)	1,806497	1,60123E-05
Gpnmb	transmembrane glycoprotein NMB	UP(D)	1,788346	7,79915E-06
Serpinb2	plasminogen activator inhibitor 2	UP(D)	1,743963	2,55263E-05

Bcl2a1b	hematopoietic Bcl-2-related protein A1	UP(D)	1,722721	0,000300459
Spdl1	Spindle apparatus coiled-coil domain-containing protein 1	UP(D)	1,385308	0,000681824
Gas2l3	growth arrest-specific 2 like 3	UP(8)	1,021524	3,40934E-07
Iron retention				
Lcn2	lipocalin 2	UP(D)	1,294766	0,000262707
Coagulation				
Procr	protein C receptor, endothelial (EPCR)	UP(D)	1,763864	0,000780925
Kng1	Kininogen-1	UP(D)	1,657824	0,000132478
Serpina10	serpin A	UP(D)	1,509817	0,000302798
Tfpi2	tissue factor pathway inhibitor 2	UP(8)	1,238266	0,000163469
F10	coagulation factor X [EC:3.4.21.6]	UP(D)	1,139223	0,011061204
F9	Coagulation factor IX ·	UP(D)	1,039342	0,000189797
Kng2	Kininogen 2	UP(8)	1,029456	0,000438362
Organogenesis				
Grem1	Gremlin-1	UP(D)	2,066399	0,000393424
Acp5	Tartrate-resistant acid phosphatase type 5	UP(8)	1,002048	1,92057E-06
Transport				
Calhm6	calcium homeostasis modulator family member 6	UP(D)	2,47254	0,000138133
Slc11a1	Natural resistance-associated macrophage protein 1	UP(D)	1,945349	6,48599E-05
Clca3b	calcium-activated chloride channel regulator 3/4	UP(D)	1,752304	8,53964E-05
Slc6a12	solute carrier family 6 (neurotransmitter transporter, betaine/GABA) member 12	UP(D)	1,67971	0,00066572
Slc7a2	solute carrier family 7 (cationic amino acid transporter), member 2	UP(D)	1,514576	0,000478625
Atp6v0d2	V-type H+-transporting ATPase subunit d	UP(D)	1,417533	3,35248E-05
Slc26a4	solute carrier family 26 (sodium-independent chloride/iodide transporter), member 4	UP(D)	1,328667	0,000965582
Stra6l	Stimulated by retinoic acid gene 6 protein-like	UP(D)	1,261297	0,000458754
Slc7a11	solute carrier family 7 (L-type amino acid transporter), member 11	UP(D)	1,252177	0,004853068
Orm1	Alpha-1-acid glycoprotein	UP(D)	1,214203	9,4431E-05
Fabp5	fatty acid-binding protein 5, epidermal	UP(8)	1,126686	0,000389619
Fabp7	fatty acid-binding protein 7, brain	UP(D)	1,11511	8,64264E-05
Orm2	Alpha-1-acid glycoprotein 2	UP(12)	1,026997	0,000944036
Clca3a2	chloride channel accessory 3A2	UP(8)	1,025085	1,35473E-06
Tmc5	Transmembrane channel-like protein 5	UP(8)	1,013459	0,000736957
Signal transduction				
Fpr3	formyl peptide receptor-like	UP(D)	1,939812	4,39963E-05
Gpr84	G protein-coupled receptor 84	UP(D)	1,376085	0,000444582
Gpr176	G protein-coupled receptor 176	UP(D)	1,057485	4,25885E-05
B430306N03Rik	RIKEN cDNA B430306N03 gene	UP(D)	1,045158	0,00029486
Hcar2	Hydroxycarboxylic acid receptor 2	UP(8)	1,009821	3,09485E-05
Others				
Olfr111	olfactory receptor	UP(D)	2,361714	6,0023E-05
Fst	Follistatin	UP(D)	2,137075	2,99182E-05
Gm4841	IRG-type G domain-containing protein	UP(D)	2,012881	6,08222E-05
Snord23		UP(D)	1,982959	1,1588E-05

Glipr1	glioma pathogenesis-related protein 1	UP(D)	1,951526	8,57867E-05
Acpp	Prostatic acid phosphatase	UP(D)	1,923057	3,04455E-05
Gpr55	G protein-coupled receptor 55	UP(D)	1,897331	1,80251E-05
Stac2	SH3 and cysteine-rich domain-containing protein 2	UP(D)	1,809912	4,17147E-05
Gm26317	NA	UP(D)	1,748444	6,47113E-05
Olfr110	olfactory receptor	UP(D)	1,697922	8,52677E-05
Mir155hg	NA	UP(D)	1,584538	0,000123754
Gm4951	Interferon-gamma-inducible GTPase Ifgga2 protein	UP(D)	1,423841	0,000283024
Guca1a	guanylate cyclase activator 1	UP(D)	1,387229	0,000104839
Slfn4	Schlafen4	UP(D)	1,364935	0,00203079
Adamts16	a disintegrin and metalloproteinase with thrombospondin motifs 16 [EC:3.4.24.-]	UP(8)	1,274171	0,000250165
Ptafr	platelet-activating factor receptor	UP(D)	1,267027	3,22525E-05
Snord35a	NA	UP(8)	1,205115	0,003104284
AW112010	NA	UP(D)	1,14618	3,28245E-05
Gm25097	NA	UP(D)	1,086533	3,50016E-05
Nts	Neurotensin/neuromedin N	UP(8)	1,082151	3,88505E-05
Adamts4	A disintegrin and metalloproteinase with thrombospondin motifs 4	UP(8)	1,069408	4,40162E-05
Mt2	Metallothionein-2	UP(12)	1,051526	0,003152007
Lhfpl2	LHFPL tetraspan subfamily member protein	UP(D)	1,051134	0,001832487
Capg	Actin regulatory protein CAP-G	UP(8)	1,035002	2,84631E-06
Marcksl1	MARCKS-related protein	UP(8)	1,030052	7,64541E-07
Ch25h		UP(8)	1,016673	4,66372E-06
No annotation found				
Gm12250	NA	UP(D)	2,123809	1,08301E-05
Speer4e	NA	UP(D)	1,863469	1,60407E-05
Gm5424	NA	UP(D)	1,75971	7,67412E-05
Creg2	protein CREG	UP(D)	1,266881	0,000306081
Ankrd55	Ankyrin repeat domain-containing protein 55	UP(D)	1,247566	3,89478E-05
Gm24535	predicted gene, 24535	UP(8)	1,17265	0,004120279
Tmem202	NA	UP(8)	1,128053	7,96434E-05
Lilr4b	leukocyte immunoglobulin-like receptor, subfamily B, member 4B	UP(D)	1,124924	0,001408036
Ceacam19	carcinoembryonic antigen-related cell adhesion molecule 19	UP(D)	1,113009	0,000528596
Gm22580	predicted gene, 22580	UP(D)	1,088951	6,05855E-05
4930430E12Rik	NA	UP(8)	1,071137	1,18712E-05
Ppp1r14bl	Protein phosphatase 1, regulatory inhibitor subunit 14B-like	UP(8)	1,070265	6,09209E-05
Gm21149	predicted gene, 21149	UP(8)	1,05712	2,23056E-05
Gm31160	mucin-12	UP(D)	1,039695	4,08935E-05
Ms4a14	Membrane-spanning 4-domains, subfamily A, member 14	UP(8)	1,037517	4,87018E-06
4930572O03Rik	NA	UP(D)	1,030066	0,000610495
4933430I17Rik		UP(8)	1,015123	5,44707E-06
Ms4a4a	Membrane-spanning 4-domains subfamily A member 3	UP(8)	1,011756	0,000824244
Gm21190	predicted gene, 21190	UP(8)	1,00553	9,44382E-05
