# Supplementary material for: Transcriptional profiling of a fungal granuloma reveals a low metabolic activity of Paracoccidioides brasiliensis yeasts and an actively regulated host immune response
Source: Front Cell Infect Microbiol. 2023 Oct 5;13:1268959. doi: 10.3389/fcimb.2023.1268959 (PMC10585178; doi:10.3389/fcimb.2023.1268959)
Supplement: Supplementary file 4 [file Table_3.pdf]

Supplementary Table 3. Downregulated mice genes.

Acession number	Protein	Expression status	Log (Fold Change)	Adjusted p-value
**Muscle contraction and organization**				
Sln	sarcolipin	DOWN(D)	-2,15699	0,001587
Myh8	myosin heavy chain 1/2/3/4/8/13/7B/15	DOWN(8)	-1,80004	0,000855
Myh1	myosin heavy chain 1/2/3/4/8/13/7B/15	DOWN(8)	-1,77174	0,001517
Tnnc1	troponin C, slow skeletal and cardiac muscles	DOWN(D)	-1,77016	0,000138
Myh4	Myosin-4	DOWN(8)	-1,7189	0,002297
Xirp2	xin actin-binding repeat containing 2	DOWN(8)	-1,65061	0,002191
Myh13	myosin heavy chain 1/2/3/4/8/13/7B/15	DOWN(8)	-1,61232	0,00143
Csrp3	cysteine and glycine-rich protein	DOWN(D)	-1,52498	0,002522
Tnnt2	troponin T, cardiac muscle	DOWN(D)	-1,45826	0,011979
Myl7	myosin regulatory light chain 7	DOWN(D)	-1,4156	0,001571
Smpx	Small muscular protein	DOWN(8)	-1,39498	0,002656
Trdn	triadin	DOWN(8)	-1,38785	0,003233
Retn	resistin	DOWN(D)	-1,37561	0,003771
Ttn	titin [EC:2.7.11.1]	DOWN(8)	-1,36393	0,000316
Lmod2	leiomodin	DOWN(8)	-1,33695	0,003945
Mylk3	myosin-light-chain kinase [EC:2.7.11.18]	DOWN(D)	-1,33469	0,001185
Myoz2	myozenin 2	DOWN(D)	-1,32969	0,005108
Myom2	myomesin	DOWN(8)	-1,31504	0,002159
Xirp1	xin actin-binding repeat containing 1	DOWN(D)	-1,29326	0,002107
Fndc5	fibronectin type III domain-containing protein 4/5	DOWN(D)	-1,26338	0,0012
Tbx20	T-box protein 20	DOWN(D)	-1,25822	0,001607
Mylk4	Myosin light chain kinase family member 4	DOWN(8)	-1,2387	0,00432
Cmya5	cardiomyopathy-associated protein 5	DOWN(8)	-1,23691	0,000695
Mybpc3	myosin-binding protein C, cardiac-type	DOWN(D)	-1,22767	0,007734
Tnni3	troponin I, cardiac muscle	DOWN(D)	-1,22069	0,003786
Kcng2	potassium voltage-gated channel subfamily G member 2	DOWN(D)	-1,2144	0,004898
Obscn	obscurin-RhoGEF [EC:2.7.11.1]	DOWN(D)	-1,19734	0,003321
Myh3	Myosin-3	DOWN(8)	-1,18001	0,007459
Myh6	myosin heavy chain 6/7	DOWN(D)	-1,17542	0,000529
Mybphl	myosin-binding protein H	DOWN(D)	-1,17353	0,008096
Synpo2l	Synaptopodin 2-like protein	DOWN(D)	-1,16931	0,008231
Ryr2	ryanodine receptor 2	DOWN(D)	-1,16664	0,000979
Kcnj5	potassium inwardly-rectifying channel subfamily J member 5	DOWN(D)	-1,16526	0,00749
Pln	Phospholamban	DOWN(D)	-1,16348	0,005872
Alpk3	alpha-kinase	DOWN(D)	-1,14371	0,002334
Casq2	calsequestrin 2	DOWN(D)	-1,14245	0,005635
Ldb3	PDZ and LIM domain protein 5/6/7	DOWN(D)	-1,14008	0,001257
Myh7	myosin heavy chain 6/7	DOWN(D)	-1,13854	0,004939
Hrc	sarcoplasmic reticulum histidine-rich calcium-binding protein	DOWN(D)	-1,13631	0,003143
Myl1	Myosin light chain 1/3, skeletal muscle isoform	DOWN(8)	-1,12704	0,006081
Trim55	Tripartite motif-containing 55	DOWN(12)	-1,12272	0,008765
Coro6	coronin-1B/1C/6	DOWN(12)	-1,11778	0,001661

Myo7b	Unconventional myosin-VIIb	DOWN(8)	-1,0907	0,000183
Tcap	Telethonin	DOWN(8)	-1,0795	0,008244
Myo18b	Myo18b protein	DOWN(8)	-1,07525	0,002078
Synm	Synemin	DOWN(8)	-1,05866	0,000487
Smyd1	[histone H3]-lysine4/36 N-trimethyltransferase SMYD [EC:2.1.1.354 2.1.1.357]	DOWN(D)	-1,05077	0,000733
Srl	sarcalumenin	DOWN(D)	-1,02872	0,002283
Actn2	Alpha-actinin-2	DOWN(12)	-1,02565	0,012348
Popdc2	Popeye domain-containing protein 2	DOWN(12)	-1,00174	0,001606
**Nervous system**				
Scn3a	voltage-gated sodium channel type III alpha	DOWN(D)	-1,47153	0,004764
Kcna1	potassium voltage-gated channel Shaker-related subfamily A member 1	DOWN(D)	-1,43254	0,005246
Gria1	glutamate receptor 1	DOWN(D)	-1,38642	0,002846
Lrtm1	leucine-rich repeats and transmembrane domains 1	DOWN(D)	-1,33558	0,004851
Slc17a7	MFS transporter, ACS family, solute carrier family 17 (sodium-dependent inorganic phosphate cotransporter), member 6/7/8	DOWN(8)	-1,32168	0,000511
Kcnh2	potassium voltage-gated channel Eag-related subfamily H member 2	DOWN(D)	-1,3173	0,013463
Ckmt2	creatine kinase [EC:2.7.3.2]	DOWN(D)	-1,26754	0,010927
Shank3	SH3 and multiple ankyrin repeat domains protein	DOWN(D)	-1,25195	0,005262
Bcan	brevican	DOWN(12)	-1,21116	0,001381
Scn1a	voltage-gated sodium channel type I alpha	DOWN(8)	-1,16132	0,004522
Kcnj12	potassium inwardly-rectifying channel subfamily J member 12/18	DOWN(8)	-1,1479	0,001293
Nptx1	neuronal pentraxin 1	DOWN(12)	-1,1369	0,000261
Slc7a10	solute carrier family 7 (D/L-type amino acid transporter), member 10	DOWN(D)	-1,13547	0,00345
Mapt	microtubule-associated protein tau	DOWN(D)	-1,10244	0,001421
Rgs6	regulator of G-protein signaling	DOWN(D)	-1,08417	0,000508
Colq	Acetylcholinesterase collagenic tail peptide	DOWN(12)	-1,07291	0,000504
Prx	Periaxin	DOWN(8)	-1,06468	0,001385
Kcna2	Potassium voltage-gated channel subfamily A member 2	DOWN(D)	-1,05175	0,000648
Dpysl5	dihydropyrimidinase-like 5	DOWN(D)	-1,04199	0,00076
Scn5a	Sodium channel protein type 5 subunit alpha	DOWN(12)	-1,0341	0,002499
Scn2a	Sodium channel protein type 2 subunit alpha	DOWN(12)	-1,01968	0,014568
**Organogenesis**				
Aard	alanine and arginine rich domain containing protein	DOWN(D)	-1,47845	0,009029
Tnxb	tenascin	DOWN(D)	-1,43596	0,006338
Bmp6	bone morphogenetic protein 6	DOWN(D)	-1,4241	0,008604
Gata4	GATA-binding protein 4	DOWN(D)	-1,36945	0,00133
Hjv	hemojuvelin	DOWN(12)	-1,24769	0,019055
Aplnr	apelin receptor	DOWN(12)	-1,22146	0,009693
Sox11	transcription factor SOX11/12 (SOX group C)	DOWN(D)	-1,13558	0,002878
Adra1b	adrenergic receptor alpha-1B	DOWN(12)	-1,10776	0,000901
Rbp4	retinol-binding protein 4	DOWN(12)	-1,09793	0,004239
Col13a1	Collagen alpha-1(XIII) chain	DOWN(D)	-1,07994	0,003297

Lrrc17		DOWN(12)	-1,02049	0,01788
Hhatl	Hedgehog acyltransferase-like protein	DOWN(8)	-1,01487	0,001763
**Lipid metabolism**				
Acacb	Acetyl-CoA carboxylase 2	DOWN(D)	-1,74821	0,020854
Cyp4a31	long-chain fatty acid omega-monooxygenase [EC:1.14.14.80]	DOWN(D)	-1,24077	0,004107
Cyp1a1	cytochrome P450 family 1 subfamily A1 [EC:1.14.14.1]	DOWN(D)	-1,23017	0,001407
Cyp2e1	cytochrome P450 family 2 subfamily E1 [EC:1.14.14.-]	DOWN(D)	-1,21999	0,003685
Plin4	perilipin-4	DOWN(D)	-1,19548	0,001265
Thrsp	thyroid hormone responsive	DOWN(D)	-1,14802	0,001858
Cyp4a10	Cytochrome P450 4A10	DOWN(8)	-1,14637	0,002356
Plin1	perilipin-1	DOWN(D)	-1,13331	0,00144
Cyp4a32	Cytochrome P450, family 4, subfamily a, polypeptide 32	DOWN(8)	-1,11972	0,003217
Tecrl	trans-2,3-enoyl-CoA reductase-like [EC:1.3.1.-]	DOWN(D)	-1,07444	0,000485
Acoxl	acyl-Coenzyme A oxidase-like	DOWN(D)	-1,05588	0,00309
**Energy metabolism**				
Cox8b	cytochrome c oxidase subunit 8	DOWN(D)	-1,3867	0,004553
Cox6a2	Cytochrome c oxidase subunit 6A2, mitochondrial	DOWN(D)	-1,22813	0,006289
Pck1	phosphoenolpyruvate carboxykinase (GTP) [EC:4.1.1.32]	DOWN(D)	-1,1931	0,000458
Glb1l3	galactosidase, beta 1 like 3	DOWN(8)	-1,16418	0,000181
Cox7a1	cytochrome c oxidase subunit 7a	DOWN(12)	-1,12561	0,008107
Pdk4	Pyruvate dehydrogenase kinase, isoenzyme 4	DOWN(8)	-1,02608	0,001765
**Pro-inflammatory**				
Bpifa1	BPI fold-containing family A	DOWN(D)	-1,23836	0,00444
Cyp2f2	cytochrome P450 family 2 subfamily F [EC:1.14.14.1]	DOWN(D)	-1,17127	0,006241
Aoc3	primary-amine oxidase [EC:1.4.3.21]	DOWN(12)	-1,09154	0,001974
Spon2	Spondin-2	DOWN(12)	-1,05775	0,023491
Tril	TLR4 interactor with leucine-rich repeats	DOWN(D)	-1,05367	0,000741
Cfd	complement factor D [EC:3.4.21.46]	DOWN(D)	-1,04996	0,001562
**Iron retention**				
Hamp	hepcidin	DOWN(D)	-1,50399	0,002563
**Cell adhesion, activation and differentiation**				
Lmntd1	lamin tail domain containing 1	DOWN(D)	-1,89557	0,003664
Fgf12	Fibroblast growth factor 12	DOWN(D)	-1,21961	0,007567
Fgfr4	fibroblast growth factor receptor 4 [EC:2.7.10.1]	DOWN(D)	-1,08753	0,005028
Nrxn2	Neurexin-2	DOWN(8)	-1,04558	0,000144
Pcdhac2	protocadherin alpha	DOWN(D)	-1,04251	0,009676
Ctnna3	Catenin alpha-3	DOWN(D)	-1,03931	0,005232
Pcdh1	Protocadherin 1	DOWN(8)	-1,02909	0,000143
Rtkn2	Rhotekin-2	DOWN(8)	-1,01313	0,000268
Megf6	Multiple epidermal growth factor-like domains protein 6	DOWN(8)	-1,0064	9,25E-05
**Regulation and tissue repair**				
Adipoq	adiponectin	DOWN(D)	-1,18296	0,003149
Bves	Blood vessel epicardial substance	DOWN(8)	-1,13558	0,000806
Ltbp4	latent transforming growth factor beta binding protein 2/3/4	DOWN(D)	-1,12502	0,004277
Gpr17	Uracil nucleotide/cysteinyl leukotriene receptor	DOWN(D)	-1,11834	0,000416
Smad6	SMAD family member 6	DOWN(8)	-1,032	7,8E-05

Pcolce2	procollagen C-endopeptidase enhancer	DOWN(D)	-1,02778	0,006885
Colgalt2	collagen beta-1,O-galactosyltransferase [EC:2.4.1.50]	DOWN(D)	-1,02311	0,000835
**cAMP and cGMP biosynthesis**				
Gnao1	guanine nucleotide-binding protein G(o) subunit alpha	DOWN(D)	-1,41267	0,012449
Rxfp1	relaxin family peptide receptor 1	DOWN(D)	-1,38785	0,013621
Adcy1	adenylate cyclase 1 [EC:4.6.1.1]	DOWN(D)	-1,24772	0,013178
Adrb3	adrenergic receptor beta-3	DOWN(D)	-1,21197	0,002305
Glp1r	glucagon-like peptide 1 receptor	DOWN(D)	-1,1953	0,000924
Nppa	natriuretic peptide A	DOWN(D)	-1,12655	0,000283
Adcy8	Adenylate cyclase type 8	DOWN(8)	-1,05135	0,001078
**Blood pressure regulation**				
Adra1a	adrenergic receptor alpha-1A	DOWN(D)	-1,73527	0,00122
Npr3	atrial natriuretic peptide clearance receptor	DOWN(D)	-1,52009	0,000709
Ace3	peptidyl-dipeptidase A [EC:3.4.15.1]	DOWN(12)	-1,15522	0,000219
Corin	atrial natriuretic peptide-converting enzyme [EC:3.4.21.-]	DOWN(12)	-1,15145	0,00092
Tnni3k	Serine/threonine-protein kinase TNNI3K	DOWN(8)	-1,00934	0,014761
**Gene/protein regulation**				
Csdc2	cold shock domain containing C2, RNA binding	DOWN(D)	-1,76843	0,001264
Ckm	E3 ubiquitin-protein ligase TRIM63	DOWN(8)	-1,68995	0,001892
Rbm20	RNA-binding protein 20	DOWN(D)	-1,67619	0,001821
Esr2	estrogen receptor beta	DOWN(D)	-1,51308	0,00245
Klhl31	kelch-like protein 31	DOWN(8)	-1,42214	0,001068
Cst8	cystatin-8	DOWN(8)	-1,30161	2,08E-06
Trim63	tripartite motif-containing protein 63 [EC:2.3.2.27]	DOWN(8)	-1,26794	0,002642
Eef1a2	elongation factor 1-alpha	DOWN(8)	-1,10921	0,001195
Nova2	Polypyrimidine tract-binding protein 2	DOWN(12)	-1,04812	0,002358
Ebf2	Transcription factor COE2	DOWN(8)	-1,02657	0,004055
Elavl2	ELAV-like protein 2	DOWN(12)	-1,02007	0,011519
**Transport**				
Kcnj3	potassium inwardly-rectifying channel subfamily J member 3	DOWN(D)	-1,51171	0,015166
Adprhl1	ADP-ribosylhydrolase like 1	DOWN(12)	-1,30803	0,003794
Aqp7	aquaporin-7	DOWN(D)	-1,2781	0,005235
Slc5a12	solute carrier family 5 (sodium-coupled monocarboxylate transporter), member 8/12	DOWN(D)	-1,26125	0,014509
Slc2a4	MFS transporter, SP family, solute carrier family 2 (facilitated glucose transporter), member 4	DOWN(D)	-1,25699	0,006532
Doc2g	double C2-like domain-containing protein gamma	DOWN(12)	-1,21385	0,002939
Lrp3	low-density lipoprotein receptor-related protein 3/10/12	DOWN(12)	-1,14528	0,013666
Fabp3	fatty acid-binding protein 3, muscle and heart	DOWN(12)	-1,11428	0,001624
Mb	myoglobin	DOWN(D)	-1,10865	0,00437
Slc38a5	solute carrier family 38 (sodium-coupled neutral amino acid transporter), member 5	DOWN(D)	-1,05608	0,002482
Atp1a2	Sodium/potassium-transporting ATPase subunit alpha-2	DOWN(8)	-1,03719	0,000263
Atp13a4	Probable cation-transporting ATPase 13A4	DOWN(8)	-1,02692	0,003493
Kcnk3	Potassium channel subfamily K member 3	DOWN(8)	-1,00991	0,000719
**Signal transduction**				

Ucp1	solute carrier family 25 (mitochondrial uncoupling protein), member 7	DOWN(8)	-1,6587	0,000398
Mpp3	MAGUK p55 subfamily member 3	DOWN(D)	-1,36734	0,002995
Scube2	signal peptide, CUB and EGF-like domain-containing protein 2	DOWN(D)	-1,16772	0,001641
Hcar1	Hydroxycarboxylic acid receptor 1	DOWN(12)	-1,16474	0,00307
Erbb4	receptor tyrosine-protein kinase erbB-4 [EC:2.7.10.1]	DOWN(D)	-1,14125	0,009709
Lgr6	leucine-rich repeat-containing G protein-coupled receptor 6	DOWN(12)	-1,07716	0,001466
Paqr9	progestin and adipoQ receptor family member IX	DOWN(D)	-1,04455	0,001391
Itga8	Integrin alpha-8	DOWN(8)	-1,02737	0,000599
**Cell cycle**				
Cidec	cell death-inducing DFFA-like effector c	DOWN(D)	-1,26985	0,017819
Gsn	Gelsolin	DOWN(8)	-1,03578	5,24E-06
**PRRs and other receptors**				
Cd209a	CD209 antigen-like protein A	DOWN(12)	-1,17387	0,008003
**Others**				
Tshr	Thyrotropin receptor	DOWN(D)	-1,76475	0,003504
Fendrr	NA	DOWN(D)	-1,40719	0,004302
Gsg1l	Germ cell-specific gene 1-like protein	DOWN(D)	-1,30943	0,005855
Krt13	Keratin, type I cytoskeletal 13	DOWN(8)	-1,29005	0,036135
Adamts17	a disintegrin and metalloproteinase with thrombospondin motifs 17 [EC:3.4.24.-]	DOWN(D)	-1,25736	0,00268
Mlip	Muscular LMNA-interacting protein	DOWN(12)	-1,18391	0,029693
Rptn	Repetin	DOWN(8)	-1,17084	0,020501
Acvr1c	activin receptor type-1C [EC:2.7.11.30]	DOWN(D)	-1,13719	0,002813
Sphkap	A-kinase anchor protein SPHKAP	DOWN(8)	-1,10536	0,000819
Car3	Carbonic anhydrase 3	DOWN(D)	-1,07018	0,014829
Irs1	Insulin receptor substrate 1	DOWN(12)	-1,06038	0,002529
Trim67	Tripartite motif-containing protein 67	DOWN(12)	-1,04076	0,000549
Mtcl1	Microtubule cross-linking factor 1	DOWN(8)	-1,03356	0,001025
Nckap5	Nck-associated protein 5-like	DOWN(8)	-1,02879	0,000565
Inmt	Indolethylamine N-methyltransferase	DOWN(8)	-1,01816	0,000597
Adamts19	disintegrin and metalloproteinase with thrombospondin motifs 19	DOWN(12)	-1,0127	0,01235
**No annotation found**				
Tmem182	NA	DOWN(8)	-1,52722	0,002295
9330159F19Rik	NA	DOWN(D)	-1,46908	0,001081
Prrt4	Proline-rich transmembrane protein 4	DOWN(D)	-1,2205	0,002247
Tspan18	Tetraspanin-18	DOWN(D)	-1,20367	0,00951
Gm34595	NA	DOWN(D)	-1,18726	0,001482
Tmem121b	NA	DOWN(8)	-1,17223	0,00053
Ankrd63	NA	DOWN(12)	-1,17033	0,000733
1110002E22Rik	RIKEN cDNA 1110002E22 gene	DOWN(8)	-1,1566	0,003219
Klra14-ps	killer cell lectin-like receptor subfamily A, member 14, pseudogene	DOWN(12)	-1,1553	0,01856
Sbk3	serine/threonine-protein kinase SBK [EC:2.7.11.1]	DOWN(D)	-1,1166	0,000692
Platr21	pluripotency associated transcript 21	DOWN(12)	-1,00796	0,008207
