# Supplementary material for: Transcriptional profiling of a fungal granuloma reveals a low metabolic activity of Paracoccidioides brasiliensis yeasts and an actively regulated host immune response
Source: Front Cell Infect Microbiol. 2023 Oct 5;13:1268959. doi: 10.3389/fcimb.2023.1268959 (PMC10585178; doi:10.3389/fcimb.2023.1268959)
Supplement: Supplementary file 5 [file Table_4.pdf]

Supplementary Table 4. Mice genes found exclusively in the infection.

Acession number	Protein
**PRRs and other receptors**	
Itln1	Intelectin-1a
Clec4b2	C-type lectin domain family 6 member A
Klrb1	Killer cell lectin-like receptor subfamily B member 1
Cd209e	CD209 antigen-like protein E
**Antigen processing and presentation**	
Trav6-1	T cell receptor alpha variable 6-1
Trav7-1	T cell receptor alpha variable 7-1
Trav6-2	T cell receptor alpha variable 6-2
Trav6d-3	T cell receptor alpha variable 6D-3
Trav6d-4	T cell receptor alpha variable 6D-4
Trav7d-4	T cell receptor alpha variable 7D-4
Trav6d-7	T cell receptor alpha variable 6D-7
Trav7n-4	T cell receptor alpha variable 7N-4
Trav9n-1	T cell receptor alpha variable 9N-1
Trav6-3	T cell receptor alpha variable 6-3
Trav7-4	T cell receptor alpha variable 7-4
Trav9-1	T cell receptor alpha variable 9-1
Trav6-5	T cell receptor alpha variable 6-5
Trav6-7-dv9	T cell receptor alpha variable 6-7-DV9
**Cytokines and chemokines**	
Il17a	interleukin 17A
Il17f	interleukin 17F
Il21	interleukin 21
Ccl1	C-C motif chemokine 1
Il13ra2	interleukin 13 receptor alpha-2
**Pro-inflammatory**	
Fcnb	ficolin
Prg3	Proteoglycan 3
Saa4	Serum amyloid A-4 protein
Mpo	Myeloperoxidase
Ctsg	Cathepsin G
**Regulation and tisse repair**	
Mmp1b	Matrix metalloproteinase-1 (interstitial collagenase) [EC:3.4.24.7]
Mmp7	Matrix metalloproteinase-7 (matrilysin, uterine) [EC:3.4.24.23]
Tff1	Trefoil factor 1
Btnl1	Butyrophilin-like protein 1
Fnd3c2	Fibronectin type III domain-containing 3C2
**Antibody**	
Igkv9-128	Immunoglobulin kappa chain variable 9-128
Igkv9-119	Immunoglobulin kappa chain variable 9-119
Igkv11-118	Immunoglobulin kappa chain variable 11-118
Igkv11-114	Immunoglobulin kappa chain variable 11-114

Igkv11-106	Immunoglobulin kappa variable 11-106
Igkv12-67	Immunoglobulin kappa chain variable 12-67
Igkv13-64	Immunoglobulin kappa chain variable 13-64
Ighd5-5	Immunoglobulin heavy diversity 5-5
Ighd5-4	Immunoglobulin heavy diversity 5-4
Ighd5-3	Immunoglobulin heavy diversity 5-3
Ighd5-2	Immunoglobulin heavy diversity 5-2
Ighv5-3	Immunoglobulin heavy variable V5-3
Ighv5-8	Immunoglobulin heavy variable V5-8
Ighv1-16	Immunoglobulin heavy variable V1-16
**Cell adhesion, activation and differentiation**	
Fgf23	Fibroblast growth factor 23
Madcam1	Mucosal addressin cell adhesion molecule 1
Lrfn5	Leucine-rich repeat and fibronectin type-III domain-containing protein 5
Prss16	Thymus-specific serine protease ·
Krt1	Keratin, type I cytoskeletal 19
**Gene/protein regulation**	
Glrp1	Glrp1 protein
Gbx2	Homeobox protein GBX-2
Prss50	Probable threonine protease PRSS50
Wfdc18	WAP four-disulfide core domain protein 18
St6galnac1	Alpha-N-acetylgalactosaminide alpha-2,6-sialyltransferase 1
Cebpe	CCAAT/enhancer-binding protein epsilon
Csta3	Cystatin A family member 3
Prss22	Brain-specific serine protease 4
**Signal transduction**	
Npbwr1	neuropeptides B/W receptor 1
Epha8	Ephrin type-A receptor 8
Pde6h	Retinal cone rhodopsin-sensitive cGMP 3',5'-cyclic phosphodiesterase subunit gamma
Drd1	D(1A) dopamine receptor
Vmn2r95	Vomeronasal 2, receptor 95
Adgrf1	Adhesion G-protein coupled receptor F1
**Cell cycle**	
Ms4a3	Membrane-spanning 4-domains subfamily A member 3
Nap1l2	Nucleosome assembly protein 1-like 2
**Coagulation**	
F13b	Coagulation factor XIII B chain
Fga	Fibrinogen alpha chain
**Lipid metabolism**	
Apoc2	Apolipoprotein C-II ·
**Transport**	
Mgarp	Protein MGARP
**Iron retention**	
Timd2	T-cell immunoglobulin and mucin domain-containing protein 2
**Others**	
Pdyn	Proenkephalin-B

Ccin	Calicin
Speer1	Spermatogenesis-associated glutamate (E)-rich protein 1
Dspp	Dentin sialophosphoprotein
Dmp1	Dentin matrix acidic phosphoprotein 1
Npy	Pro-neuropeptide Y
Prok2	Prokineticin-2
Lrrn1	Leucine-rich repeat neuronal protein 1
Dll3	Delta-like protein 3
Olfr527	Olfactory receptor 527
Olfr531	Olfactory receptor 531
Olfr46	Olfactory receptor 46
Dlgap2	Disks large-associated protein 2
Snord59a	Small nucleolar RNA, C/D box 59A
Pkd1l1	Polycystic kidney disease protein 1-like 1
Olfr56	Olfactory receptor 56
Tmem132e	Transmembrane protein 132E
Mir484	microRNA 484
Speer2	Spermatogenesis-associated glutamate (E)-rich protein 2
Tmprss3	Transmembrane protease serine 3
Olfr113	Olfactory receptor 113
Olfr114	Olfactory receptor 114
Olfr115	Olfactory receptor 115
Olfr116	Olfactory receptor 116
Olfr127	Olfactory receptor 127
Olfr128	Olfactory receptor 128
Oosp2	Oocyte-secreted protein 2
Ct55	Cancer/testis antigen 55
**No annotation found**	
Mroh3	Maestro heat-like repeat family member 3
Tmem236	Transmembrane protein 236
Gm6455	
Gm8871	
Gm5861	
Gm8890	
Gm8897	
Gm6460	
Gm8922	
Gm8926	
4933402N22Rik	
Gm10144	
4930438A08Rik	L-amino-acid oxidase [EC:1.4.3.2]
Lypd8l	
Gm11634	
1700012B07Rik	
Gm11735	
Tcstv1	2-cell-stage, variable group, member 1

Gm4131
Gm4606
Gm14718
Gm25520
Gm20807
Gm20831
