# Supplementary material for: Transcriptional profiling of a fungal granuloma reveals a low metabolic activity of Paracoccidioides brasiliensis yeasts and an actively regulated host immune response
Source: Front Cell Infect Microbiol. 2023 Oct 5;13:1268959. doi: 10.3389/fcimb.2023.1268959 (PMC10585178; doi:10.3389/fcimb.2023.1268959)
Supplement: Supplementary file 8 [file Table_7.pdf]

Supplementary Table 7. Upregulated yeasts genes.

Acession number	Protein	Expression status	Log (Fold Change)	Adjusted p-value
**Siderophores sinthesis and transport**				
PADG_00097	L-ornithine N5-oxygenase SidA	UP(D)	3,594664	2,31522E-08
PADG_00095	MFS siderochrome iron transporter 1	UP(D)	3,188131	1,11937E-08
PADG_00103	ABC transporter 1	UP(D)	2,774636	3,70273E-08
PADG_00100	Hydroxyornithine transacylase SID3	UP(D)	2,354155	3,39742E-06
PADG_00102	fusarinine C synthase	UP(D)	2,227774	5,25749E-07
PADG_00104	Oxidoreductase OXR1	UP(D)	1,957033	4,86888E-05
PADG_00099	Acyl-CoA ligase SIDI	UP(D)	1,406269	8,21301E-05
PADG_00096	Probable dehydratase NIT22	UP(D)	1,181456	0,000117426
**Virulence factors**				
PADG_02842	Superoxide dismutase, Cu-Zn	UP(D)	1,424825	3,48438E-05
PADG_04701	alcohol dehydrogenase [EC:1.1.1.-]	UP(8)	1,042112	1,75152E-06
PADG_04274	polysaccharide synthase Cps1	UP(8)	1,027865	0,001102958
**Gene/protein regulation**				
PADG_01203	Methyltransf_2 domain-containing protein	UP(D)	2,761002	2,59574E-07
PADG_01204	N-acetyltransferase domain-containing protein	UP(D)	1,337301	0,000297601
PADG_02981	Putative intracellular protease/amidase	UP(D)	1,326706	3,86945E-05
**Amino acid metabolism**				
PADG_07440	Amino acid permease	UP(D)	1,812242	8,64386E-07
PADG_08406	O-acetylhomoserine/O-acetylserine sulfhydrylase [EC:2.5.1.49 2.5.1.47]	UP(D)	1,637297	0,000873306
PADG_00215	aromatic-L-amino-acid/L-tryptophan decarboxylase [EC:4.1.1.28 4.1.1.105]	UP(D)	1,514618	0,000419168
PADG_02214	4-aminobutyrate aminotransferase / (S)-3-amino-2-methylpropionate transaminase [EC:2.6.1.19 2.6.1.22]	UP(8)	1,140801	4,11864E-06
**Transport**				
PADG_00917	copper-transporting P-type ATPase	UP(D)	1,773482	0,000919061
PADG_08041	MFS transporter	UP(D)	1,566054	3,94814E-05
**Other metabolisms**				
PADG_06196	Flavin oxidoreductase hxnT	UP(D)	1,98727	4,73024E-06
PADG_03085	Mannose-P-dolichol utilization defect 1 protein homolog	UP(12)	1,076101	0,000243588
PADG_05433	pyridoxamine 5'-phosphate oxidase [EC:1.4.3.5]	UP(8)	1,032033	5,69744E-05
**Cell cylce**				
PADG_07329	Pfam: hepsim	UP(D)	2,313777	0,001041351
**Signal transduction**				
PADG_00105	Response regulatory domain-containing protein	UP(D)	1,071989	0,009366313
**No annotation found**				
PADG_07436	Uncharacterized protein	UP(12)	1,174975	0,002752736
PADG_02015	Uncharacterized protein	UP(8)	1,159171	5,5054E-06

PADG_04104	Uncharacterized protein	UP(8)	1,158234	5,37787E-05
PADG_04228	Uncharacterized protein	UP(12)	1,042927	0,010131922
PADG_01316	Uncharacterized protein	UP(8)	1,035318	0,001020631
PADG_08359	Short-chain dehydrogenase/reductase 2	UP(8)	1,014308	0,00023174
PADG_08475	Bys1 family protein	UP(D)	1,013813	2,79248E-05
