# Supplementary material for: Transcriptional profiling of a fungal granuloma reveals a low metabolic activity of Paracoccidioides brasiliensis yeasts and an actively regulated host immune response
Source: Front Cell Infect Microbiol. 2023 Oct 5;13:1268959. doi: 10.3389/fcimb.2023.1268959 (PMC10585178; doi:10.3389/fcimb.2023.1268959)
Supplement: Supplementary file 9 [file Table_8.pdf]

Supplementary Table 8. Downregulated yeasts genes.

Acession number	Protein	Expression status	Log (Fold Change)	Adjusted p-value
**Gene/protein regulation**				
PADG_00073	SAM-dependent methyltransferase UbiE/COQ5 family protein	DOWN(8)	-1,39758	0,001413293
PADG_00517	DnaJ domain-containing protein	DOWN(D)	-1,27835	0,000881547
PADG_04314	Zn(2)-C6 fungal-type domain-containing protein	DOWN(8)	-1,24281	4,13477E-05
PADG_05381	RNA recognition motif containing protein	DOWN(12)	-1,23434	0,001365058
PADG_04889	Zn(2)-C6 fungal-type domain-containing protein	DOWN(D)	-1,23166	0,002812763
PADG_05497	GATA-binding protein, other eukaryote	DOWN(D)	-1,22538	0,002127227
PADG_02195	DNA repair protein Swi5/Sae3	DOWN(8)	-1,21794	0,001339732
PADG_04429	Structure-specific endonuclease subunit SLX4	DOWN(8)	-1,14817	0,000989517
PADG_12501	CCHC-type domain-containing protein	DOWN(12)	-1,12982	0,001179446
PADG_11403	methyltransferase	DOWN(12)	-1,11346	0,000977374
PADG_11441	HTH CENPB-type domain-containing protein	DOWN(8)	-1,11024	0,000930833
PADG_01793	Nuclear architecture-related protein 1	DOWN(D)	-1,10606	0,006768258
PADG_03377	Ubiquitin-like-specific protease	DOWN(12)	-1,09838	0,001866278
PADG_03791	type IV protein arginine methyltransferase [EC:2.1.1.322]	DOWN(8)	-1,08822	0,005202474
PADG_01183	TAM domain methyltransferase	DOWN(12)	-1,07928	0,002060487
PADG_00670	TAM domain methyltransferase	DOWN(D)	-1,07707	0,001051091
PADG_00411	C6 transcription factor (Ctf1B)	DOWN(D)	-1,05195	0,005550265
PADG_12203	ribosomal biogenesis protein LAS1	DOWN(8)	-1,0468	0,000603147
PADG_00759	prefoldin subunit 4	DOWN(8)	-1,04389	0,003743707
PADG_01958	Translation initiation factor 4E	DOWN(12)	-1,04272	0,007995696
PADG_02035	Non-specific serine/threonine protein kinase	DOWN(8)	-1,04168	0,004765044
PADG_03501	CCHC-type domain-containing protein	DOWN(12)	-1,04116	0,002060487
PADG_04908	InfB (Translation initiation factor) domain-containing protein	DOWN(12)	-1,03846	0,00417871
PADG_03236	DNA-binding protein creA	DOWN(12)	-1,03587	0,004856195
PADG_00373	NmrA domain-containing protein	DOWN(8)	-1,03413	0,000626493
PADG_04616	Smad nuclear-interacting protein 1	DOWN(12)	-1,03304	0,004737069
PADG_06413	C6 finger domain transcription factor nscR	DOWN(8)	-1,00975	0,008026004
PADG_05350	Surfeit locus protein 5 subunit 22 of mediator complex-domain-containing protein	DOWN(8)	-1,00864	0,016467202
PADG_04917	SprT-like domain-containing protein	DOWN(12)	-1,00771	0,005081481

PADG_00716	eukaryotic translation initiation factor 2C	DOWN(8)	-1,00712	8,67927E-05
PADG_07794	EKC/KEOPS complex subunit BUD32	DOWN(12)	-1,00227	0,017543585
**Energy metabolism**				
PADG_07982	3-oxoacyl-[acyl-carrier-protein] reductase	DOWN(D)	-1,6235	0,000507095
PADG_11844	aconitate hydratase	DOWN(D)	-1,52072	0,010419846
PADG_07615	glucan 1,3-beta-glucosidase [EC:3.2.1.58]	DOWN(D)	-1,47922	0,000308752
PADG_00393	hexokinase [EC:2.7.1.1]	DOWN(12)	-1,38879	0,000134246
PADG_03602	acyl-CoA dehydrogenase	DOWN(D)	-1,32085	0,000697344
PADG_04322	rhodocoxin reductase	DOWN(8)	-1,30477	0,000335187
PADG_04699	NADH dehydrogenase (ubiquinone) 1 alpha subcomplex subunit 2	DOWN(D)	-1,24476	0,000708432
PADG_00403	benzoate 4-monooxygenase cytochrome P450	DOWN(D)	-1,17316	0,000793491
PADG_08013	succinate dehydrogenase (ubiquinone) iron-sulfur subunit [EC:1.3.5.1]	DOWN(12)	-1,14543	0,001432556
PADG_06764	succinate dehydrogenase (ubiquinone) membrane anchor subunit	DOWN(D)	-1,10912	0,003020469
PADG_03395	sulfite reductase (NADPH) hemoprotein beta-component [EC:1.8.1.2]	DOWN(8)	-1,10366	3,97735E-06
PADG_05273	PNPLA domain-containing protein	DOWN(12)	-1,07871	0,000294774
PADG_05290	Cytochrome b5 heme-binding domain-containing protein	DOWN(D)	-1,07453	0,005482746
PADG_08216	NADH dehydrogenase (ubiquinone) Fe-S protein 8 [EC:7.1.1.2]	DOWN(D)	-1,05558	0,003519666
PADG_03050	Mitochondrial zinc maintenance protein 1, mitochondria	DOWN(8)	-1,01841	0,002043982
**Cell cycle**				
PADG_03379	Ras homolog gene family, member A	DOWN(8)	-1,47733	0,000980373
PADG_03907	Calmodulin-binding protein Sha1	DOWN(D)	-1,32769	0,002625672
PADG_06942	kinetochore protein Spc24, fungi type	DOWN(8)	-1,25839	0,00733853
PADG_03022	PLK/PLK1 protein kinase	DOWN(D)	-1,25446	0,002207404
PADG_03336	annexin ANXC4	DOWN(12)	-1,24611	0,002362671
PADG_00567	Cell division control protein	DOWN(D)	-1,24467	0,007797456
PADG_02357	kinetochore protein Spc25, fungi type	DOWN(D)	-1,23929	0,000591552
PADG_11709	serine/threonine-protein kinase TTK/MPS1 [EC:2.7.12.1]	DOWN(D)	-1,23479	5,54006E-05
PADG_01088	Ase1/PRC1/MAP65 family protein	DOWN(8)	-1,20056	0,005994429
PADG_03219	Myosin regulatory light chain cdc4	DOWN(8)	-1,18769	0,006834469
PADG_02243	Spo12 family protein	DOWN(12)	-1,12485	0,002901531
PADG_01683	GTPase binding protein Rid1	DOWN(D)	-1,1012	0,000230408
PADG_06409	protein OPY2	DOWN(12)	-1,07115	0,005650656
PADG_04667	NIMA (never in mitosis gene a)-related kinase 2 [EC:2.7.11.1]	DOWN(12)	-1,07109	0,007872998

PADG_01930	Uncharacterized protein	DOWN(8)	-1,06836	5,69744E-05
**Virulence factors**				
PADG_01954	superoxide dismutase, Fe-Mn family [EC:1.15.1.1]	DOWN(D)	-2,55152	5,85186E-06
PADG_02598	beta-glucosidase	DOWN(D)	-1,6122	1,11583E-06
PADG_05660	Fungal nitric oxide reductase	DOWN(D)	-1,38287	0,007156901
PADG_01716	alcohol dehydrogenase zinc-binding	DOWN(D)	-1,35725	0,000574079
PADG_01987	alcohol dehydrogenase	DOWN(8)	-1,2762	0,007506831
PADG_05344	thioredoxin-dependent peroxiredoxin [EC:1.11.1.24]	DOWN(D)	-1,22454	0,006713465
PADG_12387	MFS fluconazole resistance protein	DOWN(D)	-1,22205	0,000540352
PADG_03691	Glycosidase	DOWN(12)	-1,20574	0,006151669
PADG_05140	Protein SUR7	DOWN(8)	-1,08391	0,000408789
**Amino acid metabolism**				
PADG_01952	asparagine synthase (glutamine-hydrolysing) [EC:6.3.5.4]	DOWN(D)	-1,41864	0,000120708
PADG_02278	5-aminolevulinate synthase [EC:2.3.1.37]	DOWN(D)	-1,4147	0,003543567
PADG_03785	ATP phosphoribosyltransferase	DOWN(8)	-1,28408	0,000449676
PADG_00349	kynureninase [EC:3.7.1.3]	DOWN(12)	-1,25626	0,004406369
PADG_07609	dihydroxy-acid dehydratase [EC:4.2.1.9]	DOWN(D)	-1,23921	0,001361747
**Other metabolisms**				
PADG_07076	flavin-binding monooxygenase	DOWN(D)	-2,60886	2,60265E-07
PADG_00106	ectonucleotide pyrophosphatase/phosphodiesterase	DOWN(8)	-1,54321	4,92606E-05
PADG_07960	tetraspanin	DOWN(D)	-1,42462	0,001086297
PADG_02384	porphobilinogen synthase [EC:4.2.1.24]	DOWN(D)	-1,38467	0,000980843
PADG_12236	thiamine pyrophosphokinase [EC:2.7.6.2]	DOWN(12)	-1,35168	0,000471889
PADG_00348	7,8-dihydropterin-6-yl-methyl-4-(beta-D-ribofuranosyl)aminobenzene 5'-phosphate synthase [EC:2.5.1.105]	DOWN(8)	-1,1748	0,000429496
PADG_06740	betaine aldehyde dehydrogenase	DOWN(D)	-1,16951	0,000879492
PADG_07849	N5-hydroxyornithine acetyltransferase [EC:2.3.1.-]	DOWN(12)	-1,12162	0,005081481
PADG_07986	gamma-glutamyltranspeptidase / glutathione hydrolase [EC:2.3.2.2 3.4.19.13]	DOWN(12)	-1,08719	0,004937728
PADG_05855	gluconolactonase [EC:3.1.1.17]	DOWN(8)	-1,08609	0,009950104
PADG_02285	Cytochrome P450-DIT2	DOWN(12)	-1,08483	0,002342264
PADG_02123	Aflatoxin B1 aldehyde reductase member 2	DOWN(12)	-1,08236	0,009558458
PADG_03920	Tyrosine/serine protein phosphatase	DOWN(12)	-1,06263	0,004074648
PADG_04432	alpha-amylase [EC:3.2.1.1]	DOWN(D)	-1,06127	0,00292087
PADG_05184	Ethanolamine-phosphate cytidylyltransferase	DOWN(12)	-1,05906	0,012720465
PADG_06123	Cytidine deaminase-like protein	DOWN(8)	-1,03202	0,001677866

PADG_01233	4HBT domain-containing protein	DOWN(8)	-1,02317	0,004030614
PADG_00400	Beta-hexosaminidase	DOWN(12)	-1,00129	0,002159862
**Transport**				
PADG_04197	ATP-binding cassette, subfamily G (WHITE), member 2, PDR	DOWN(D)	-1,84782	0,000495824
PADG_03294	P-type Na+/K+ transporter [EC:7.2.2.3 7.2.2.-]	DOWN(D)	-1,62896	1,27763E-06
PADG_04541	pH-response regulator protein palA/RIM20	DOWN(D)	-1,34939	4,61534E-05
PADG_05084	solute carrier family 31 (copper transporter), member 1	DOWN(D)	-1,29919	0,001426004
PADG_00924	kinesin family protein	DOWN(8)	-1,2839	0,00294678
PADG_04803	NADPH oxidase 1	DOWN(12)	-1,24725	0,00271291
PADG_11807	FAD-binding FR-type domain-containing protein	DOWN(8)	-1,20878	0,001125053
PADG_05835	ATP-binding cassette, subfamily B (MDR/TAP), member 1 [EC:7.6.2.2]	DOWN(D)	-1,19516	0,011001768
PADG_02809	MFS transporter, NNP family, nitrate/nitrite transporter	DOWN(D)	-1,17178	0,001264654
PADG_00150	solute carrier family 20 (sodium-dependent phosphate transporter)	DOWN(12)	-1,1267	0,000887073
PADG_07610	MFS transporter	DOWN(8)	-1,0765	9,48131E-05
PADG_01256	MFS sugar transporter	DOWN(8)	-1,00564	0,038918944
**Signal transduction**				
PADG_07386	ER membrane protein Wsc4	DOWN(D)	-1,68009	0,002359476
PADG_04598	guanine nucleotide-binding protein subunit alpha, other	DOWN(D)	-1,29413	5,75891E-05
PADG_04943	cell wall integrity and stress response component	DOWN(12)	-1,14422	0,005000612
PADG_05209	PH domain-containing protein	DOWN(12)	-1,07296	0,001786692
**Membrane antigen**				
PADG_04649	expression library immunization antigen 1	DOWN(D)	-1,36685	0,000601332
**Lipid metabolism**				
PADG_12430	long-chain acyl-CoA synthetase [EC:6.2.1.3]	DOWN(12)	-1,1162	0,006151669
**No annotation found**				
PADG_00497	Uncharacterized protein	DOWN(D)	-2,90016	3,70273E-08
PADG_08212	Uncharacterized protein	DOWN(D)	-2,35091	3,60126E-06
PADG_05763	oxidoreductase	DOWN(D)	-2,0453	2,86135E-06
PADG_04544	Uncharacterized protein	DOWN(D)	-1,91389	0,000551501
PADG_05362	NACHT domain-containing protein	DOWN(D)	-1,68802	0,00019332
PADG_01513	Uncharacterized protein	DOWN(D)	-1,67529	3,73096E-06
PADG_04482	Uncharacterized protein	DOWN(D)	-1,56185	0,000938842
PADG_04785	Uncharacterized protein	DOWN(D)	-1,50151	0,00011005
PADG_11139	Uncharacterized protein	DOWN(D)	-1,4762	0,018354614
PADG_00503	Uncharacterized protein	DOWN(D)	-1,46718	0,000327817
PADG_07981	Uncharacterized protein	DOWN(D)	-1,45884	0,003403167

PADG_05754	Uncharacterized protein	DOWN(12)	-1,45594	9,73083E-05
PADG_04024	Uncharacterized protein	DOWN(12)	-1,44864	7,38458E-05
PADG_03372	Uncharacterized protein	DOWN(12)	-1,44838	0,005023691
PADG_05781	Uncharacterized protein	DOWN(12)	-1,42829	0,000183939
PADG_12190	Uncharacterized protein	DOWN(12)	-1,42666	0,000243588
PADG_07394	Uncharacterized protein	DOWN(8)	-1,4086	0,004820891
PADG_05485	Uncharacterized protein	DOWN(12)	-1,39678	7,84343E-05
PADG_03113	Uncharacterized protein	DOWN(D)	-1,38151	0,003089127
PADG_08719	Uncharacterized protein	DOWN(8)	-1,3416	0,00272488
PADG_07065	Uncharacterized protein	DOWN(D)	-1,33361	0,000686818
PADG_00818	Uncharacterized protein	DOWN(D)	-1,33086	9,32678E-05
PADG_05279	Uncharacterized protein	DOWN(D)	-1,2711	0,001499742
PADG_02439	Uncharacterized protein	DOWN(8)	-1,25348	4,87376E-05
PADG_05859	Uncharacterized protein	DOWN(8)	-1,24861	0,002407308
PADG_12191	Uncharacterized protein	DOWN(8)	-1,24756	0,000240148
PADG_04292	Uncharacterized protein	DOWN(D)	-1,23515	0,002205478
PADG_07555	Uncharacterized protein	DOWN(12)	-1,23084	0,002362671
PADG_01986	Uncharacterized protein	DOWN(8)	-1,21794	6,32695E-05
PADG_07811	NAD dependent epimerase/dehydratase family protein	DOWN(D)	-1,20784	0,00231787
PADG_07578	Uncharacterized protein	DOWN(8)	-1,20592	0,000261883
PADG_07577	Uncharacterized protein	DOWN(12)	-1,19359	0,002066219
PADG_11879	Uncharacterized protein	DOWN(D)	-1,18278	0,002224044
PADG_00404	Uncharacterized protein	DOWN(D)	-1,18031	0,005307509
PADG_00870	Uncharacterized protein	DOWN(D)	-1,17394	0,003288465
PADG_12220	Uncharacterized protein	DOWN(12)	-1,16256	0,000991469
PADG_03654	Uncharacterized protein	DOWN(D)	-1,16002	0,001306557
PADG_02424	Uncharacterized protein	DOWN(8)	-1,15547	0,001755113
PADG_11893	Uncharacterized protein	DOWN(12)	-1,15396	0,004605148
PADG_07995	Uncharacterized protein	DOWN(D)	-1,15316	0,005059482
PADG_04591	Uncharacterized protein	DOWN(12)	-1,14988	0,003614564
PADG_02738	Uncharacterized protein	DOWN(D)	-1,14449	0,00069151
PADG_07537	Uncharacterized protein	DOWN(8)	-1,13798	0,004147573
PADG_00496	Protoglobin domain-containing protein	DOWN(D)	-1,1375	0,003686323
PADG_05629	heat shock factor-binding protein 1	DOWN(12)	-1,13576	0,003614564
PADG_05755	Uncharacterized protein	DOWN(12)	-1,13568	0,001171024
PADG_06800	Uncharacterized protein	DOWN(D)	-1,13544	0,002380642
PADG_00356	Uncharacterized protein	DOWN(12)	-1,13506	0,005176819
PADG_05316	Uncharacterized protein	DOWN(8)	-1,13486	0,000416166
PADG_05722	Uncharacterized protein	DOWN(D)	-1,1342	0,001369932
PADG_05496	Uncharacterized protein	DOWN(8)	-1,13408	0,000477489
PADG_06694	Uncharacterized protein	DOWN(12)	-1,13056	0,006412308
PADG_01403	acyltransferase	DOWN(D)	-1,12978	0,001403147
PADG_03023	Oxidorredutase	DOWN(12)	-1,12773	0,000885066
PADG_07498	Oxidoreductase	DOWN(8)	-1,12532	0,000861917

PADG_05753	Uncharacterized protein	DOWN(8)	-1,12338	0,000135921
PADG_08467	Uncharacterized protein	DOWN(D)	-1,1209	0,00138618
PADG_06871	Uncharacterized protein	DOWN(8)	-1,10938	6,70576E-07
PADG_06151	Uncharacterized protein	DOWN(8)	-1,10801	0,000230884
PADG_03140	phosphotransferase enzyme family protein	DOWN(12)	-1,10509	0,002752736
PADG_11103	Uncharacterized protein	DOWN(8)	-1,097	0,001040947
PADG_11303	Uncharacterized protein	DOWN(12)	-1,09614	0,003531071
PADG_05055	Uncharacterized protein	DOWN(8)	-1,09309	0,003184251
PADG_00883	Uncharacterized protein	DOWN(D)	-1,08791	0,002843609
PADG_06173	Uncharacterized protein	DOWN(D)	-1,08404	0,000693875
PADG_02853	Uncharacterized protein	DOWN(12)	-1,08179	0,004015094
PADG_07231	Uncharacterized protein	DOWN(12)	-1,08157	0,00076621
PADG_11129	Uncharacterized protein	DOWN(8)	-1,07924	0,000525461
PADG_03951	serine/threonine-protein kinase SRPK3 [EC:2.7.11.1]	DOWN(12)	-1,07696	0,007629575
PADG_04507	Uncharacterized protein	DOWN(12)	-1,06913	0,002957284
PADG_12300	Uncharacterized protein	DOWN(12)	-1,06597	0,006211405
PADG_11071	DAGKc domain-containing protein	DOWN(12)	-1,05966	0,002193196
PADG_07110	Uncharacterized protein	DOWN(D)	-1,05714	0,004321106
PADG_06452	Uncharacterized protein	DOWN(8)	-1,0547	8,33183E-05
PADG_00271	XPG_I_2 domain-containing protein	DOWN(12)	-1,05445	0,011517685
PADG_05127	Uncharacterized protein	DOWN(12)	-1,04944	0,002362671
PADG_06738	Pkinase_fungal domain-containing protein	DOWN(8)	-1,04918	0,000429496
PADG_07218	Uncharacterized protein	DOWN(12)	-1,04713	0,007910191
PADG_02695	Uncharacterized protein	DOWN(12)	-1,04531	0,006151669
PADG_00657	Uncharacterized protein	DOWN(8)	-1,04435	0,002695168
PADG_12146	Uncharacterized protein	DOWN(12)	-1,04241	0,006519973
PADG_12416	Uncharacterized protein	DOWN(8)	-1,04155	0,002119758
PADG_01988	Uncharacterized protein	DOWN(D)	-1,03948	0,011937741
PADG_05151	Uncharacterized protein	DOWN(12)	-1,03767	0,00458842
PADG_01875	Uncharacterized protein	DOWN(12)	-1,03576	0,000991469
PADG_03398	Uncharacterized protein	DOWN(12)	-1,03335	0,002097094
PADG_02527	Oxidoreductase	DOWN(12)	-1,03289	0,002544685
PADG_00385	Uncharacterized protein	DOWN(8)	-1,03252	0,00489703
PADG_08414	Uncharacterized protein	DOWN(8)	-1,02874	0,004765044
PADG_08296	Uncharacterized protein	DOWN(8)	-1,02837	0,00406934
PADG_07387	integral membrane protein	DOWN(12)	-1,02707	0,002007335
PADG_12182	Uncharacterized protein	DOWN(8)	-1,02629	0,000910933
PADG_07921	Uncharacterized protein	DOWN(12)	-1,02341	0,001314486
PADG_00769	Uncharacterized protein	DOWN(8)	-1,02239	0,000148228
PADG_02723	F-box domain-containing protein	DOWN(8)	-1,02231	0,005606739
PADG_08224	CBS domain-containing protein	DOWN(12)	-1,01917	0,001451516
PADG_12111	Uncharacterized protein	DOWN(12)	-1,0178	0,003149892
PADG_11362	Goodbye domain-containing protein	DOWN(8)	-1,01503	0,000416166
PADG_07074	Uncharacterized protein	DOWN(12)	-1,0115	0,012414806

PADG_04158	Uncharacterized protein	DOWN(8)	-1,0108	0,003914098
PADG_08421	Uncharacterized protein	DOWN(12)	-1,01068	0,002890692
PADG_06089	SET domain-containing protein	DOWN(12)	-1,00784	0,005120214
PADG_11867	Uncharacterized protein	DOWN(8)	-1,00708	0,002742621
PADG_05498	Uncharacterized protein	DOWN(8)	-1,0027	0,001841351
PADG_03613	Uncharacterized protein	DOWN(12)	-1,00168	0,028596545
