# Supplementary material for: Transcriptional profiling of a fungal granuloma reveals a low metabolic activity of Paracoccidioides brasiliensis yeasts and an actively regulated host immune response
Source: Front Cell Infect Microbiol. 2023 Oct 5;13:1268959. doi: 10.3389/fcimb.2023.1268959 (PMC10585178; doi:10.3389/fcimb.2023.1268959)
Supplement: Supplementary file 10 [file Table_9.pdf]

Supplementary Table 9. Downregulated yeast proteins present in the granulomatous lesions.

Acession number	Protein	Expression status	Log (Fold Change)	Adjusted p-value
**Gene/protein regulation**				
C1GK81	SF3b1 domain-containing protein	DOWN(D)	-8,24706	1,67E-06
C1GKC9	Hsp90-like protein	DOWN(8)	-7,31103	4,46E-05
C1G6I0	heat shock 70kDa protein 4	DOWN(8)	-6,75185	4,8E-05
C1GGQ1	peptidyl-prolyl isomerase D [EC:5.2.1.8]	DOWN(D)	-6,41275	6,51E-05
C1GDK4	Pre-mRNA splicing factor	DOWN(8)	-6,19463	0,000537
C1G0E5	40S ribosomal protein S14	DOWN(8)	-5,76095	0,001957
C1G3B1	40S ribosomal protein S12	DOWN(8)	-5,68669	0,000183
A0A0A0HVT2	26S proteasome regulatory subunit T3	DOWN(D)	-5,52288	8,45E-05
C1GJT7	mannosyl-oligosaccharide alpha-1,3-glucosidase [EC:3.2.1.207]	DOWN(8)	-5,1066	0,000974
C1GIA6	GrpE protein homolog	DOWN(D)	-4,90736	0,000115
A0A0A0HYV6	60S ribosomal protein L5	DOWN(D)	-4,83992	0,001244
C1G3T9	Ubiquitin-60S ribosomal protein L40	DOWN(D)	-4,81993	4,55E-06
C1GLX8	chaperonin GroEL	DOWN(D)	-4,75563	0,000957
C1GA06	Peptidyl-prolyl cis-trans isomerase (PPIase) (EC 5.2.1.8)	DOWN(8)	-4,751	5,76E-05
C1G953	Prohibitin	DOWN(8)	-4,75032	0,00017
C1G5P0	pre-mRNA-splicing helicase BRR2 [EC:3.6.4.13]	DOWN(D)	-4,64777	2,39E-05
C1GCK9	KOW domain-containing protein	DOWN(D)	-4,32901	0,000367
C1G7B3	Fungal_trans domain-containing protein	DOWN(D)	-4,25803	8,78E-05
C1G159	26S proteasome regulatory subunit T5	DOWN(8)	-4,18131	0,000219
C1GEW2	Single-stranded DNA-binding protein	DOWN(D)	-4,14922	3,58E-05
C1GJD9	Dolichyl-diphosphooligosaccharide--protein glycosyltransferase subunit WBP1 (Oligosaccharyl transferase subunit WBP1)	DOWN(D)	-3,95437	4,95E-05
C1G3P2	histidyl-tRNA synthetase [EC:6.1.1.21]	DOWN(D)	-3,88974	0,002497
C1G3P9	calnexin	DOWN(12)	-3,47349	0,000455
C1G4M5	Translation initiation factor RLI1	DOWN(12)	-3,44291	0,002348
C1G0B0	26S protease regulatory subunit 8	DOWN(D)	-3,40898	0,000184
C1GAE3	mitochondrial transcription factor 1	DOWN(8)	-3,30744	0,002154
C1GC92	Prohibitin	DOWN(D)	-3,2977	0,000783
C1G0E3	40S ribosomal protein S16	DOWN(8)	-3,18882	0,027828
C1GKL7	60S ribosomal protein L12	DOWN(D)	-3,12329	0,00097
A0A0A0HWY2	Ribosomal_L6 domain-containing protein	DOWN(D)	-3,11933	0,000447
C1GCN9	mannosyl-oligosaccharide glucosidase [EC:3.2.1.106]	DOWN(8)	-2,96435	0,000537
C1GIZ3	elongation factor 3	DOWN(8)	-2,91132	0,01155
C1GEV1	RNA helicase (EC 3.6.4.13)	DOWN(D)	-2,68877	0,026883

C1GC66	60S ribosomal protein L22	DOWN(D)	-2,55525	0,003243
C1G0P0	molecular chaperone DnaK	DOWN(12)	-2,42964	0,026103
C1GEE2	protein disulfide-isomerase A6 [EC:5.3.4.1]	DOWN(8)	-2,22092	0,019177
C1G5L7	Dolichyl-phosphate-mannose--protein mannosyltransferase (EC 2.4.1.109)	DOWN(8)	-1,56863	0,023974
C1GAW5	pre-mRNA-processing-splicing factor 8	DOWN(8)	-1,43368	0,049192
C1FZ98	Mitochondrial metallochaperone Sco1	DOWN(8)	-1,40066	0,027603
**Energy metabolism**				
C1GLB8	Malate dehydrogenase (EC 1.1.1.37)	DOWN(8)	-8,76505	3,33E-06
C1GIX4	Malate dehydrogenase (EC 1.1.1.37)	DOWN(D)	-8,55589	2,1E-05
C1GJI4	Transaldolase (EC 2.2.1.2)	DOWN(12)	-7,0939	3,57E-05
C1G547	Pyruvate dehydrogenase (Acetyl-transferring) E1 component, alpha subunit	DOWN(D)	-6,41594	7,72E-05
C1GIF6	ATP synthase subunit 5, mitochondrial	DOWN(D)	-6,39389	5,17E-05
C1GHR9	Acyl-CoA dehydrogenase	DOWN(D)	-6,04624	1,37E-05
C1G421	acetyl-CoA acyltransferase 1 [EC:2.3.1.16]	DOWN(D)	-5,80073	9,78E-05
Q1XA91	Cytochrome c oxidase subunit 2	DOWN(8)	-5,67874	0,00017
C1GBZ4	glutamate dehydrogenase (NADP+) [EC:1.4.1.4]	DOWN(8)	-5,63618	0,000217
C1G1C8	Fructose-bisphosphate aldolase (FBP aldolase) (EC 4.1.2.13)	DOWN(D)	-5,39239	0,000179
C1G911	Alternative oxidase (EC 1.-.-.-)	DOWN(8)	-5,33852	0,001063
C1G2W2	Pyruvate kinase (EC 2.7.1.40)	DOWN(D)	-5,32367	0,00092
C1G736	acyl-CoA oxidase [EC:1.3.3.6]	DOWN(D)	-5,10798	0,000658
C1FZL2	Succinate dehydrogenase [ubiquinone] flavoprotein subunit, mitochondrial (EC 1.3.5.1)	DOWN(D)	-4,84814	0,001121
C1GAG3	Isocitrate dehydrogenase [NADP] (EC 1.1.1.42)	DOWN(8)	-4,8401	0,00021
C1GDR6	Uncharacterized protein	DOWN(D)	-4,83387	0,000721
C1G098	NADH-ubiquinone oxidoreductase 21 kDa subunit	DOWN(8)	-4,66391	0,000522
C1GIX7	pyruvate dehydrogenase E2 component (dihydrolipoamide acetyltransferase) [EC:2.3.1.12]	DOWN(D)	-4,53902	0,000149
C1GGE5	acetyl-CoA C-acetyltransferase [EC:2.3.1.9]	DOWN(8)	-4,49206	0,000276
A0A0A0HTA9	Cytochrome c1, heme protein, mitochondrial	DOWN(D)	-4,42711	0,000553
C1G2P3	enoyl-CoA hydratase [EC:4.2.1.17]	DOWN(D)	-4,37849	2,82E-05
C1G3G7	Isocitrate lyase	DOWN(8)	-4,31998	0,00124
C1GM03	Cytochrome b-c1 complex subunit 2	DOWN(D)	-4,10832	0,000135
C1FZY1	L-lactate dehydrogenase (cytochrome) [EC:1.1.2.3]	DOWN(8)	-3,96582	0,000926
C1GLZ6	Citrate synthase	DOWN(D)	-3,84389	0,001893

C1G4S5	Respiratory supercomplex factor 1, mitochondrial	DOWN(D)	-3,61144	0,000846
A0A0A0HST0	F-type H+-transporting ATPase subunit g	DOWN(D)	-3,39483	0,000612
C1G6K0	Isocitrate dehydrogenase [NAD] subunit, mitochondrial	DOWN(D)	-3,35986	7,17E-05
C1G413	NADH dehydrogenase [ubiquinone] flavoprotein 1, mitochondrial (EC 7.1.1.2)	DOWN(D)	-3,30789	0,004191
C1G294	succinyl-CoA synthetase alpha subunit [EC:6.2.1.4 6.2.1.5]	DOWN(D)	-3,29572	0,003605
C1GB03	ATP synthase F1 subunit epsilon	DOWN(D)	-3,28825	0,005182
C1GCI7	Isocitrate lyase	DOWN(8)	-3,26015	0,000788
C1G9P1	Isocitrate dehydrogenase [NAD] subunit, mitochondrial	DOWN(D)	-3,18604	4,85E-05
C1G0C7	succinyl-CoA synthetase beta subunit [EC:6.2.1.4 6.2.1.5]	DOWN(8)	-2,74039	0,024202
C1G4D1	Dihydrolipoamide acetyltransferase component of pyruvate dehydrogenase complex (EC 2.3.1.-)	DOWN(12)	-2,44792	0,002062
C1GDV0	Cytochrome b-c1 complex subunit Rieske, mitochondrial (EC 7.1.1.8)	DOWN(8)	-2,4296	0,007373
C1G056	Pyruvate dehydrogenase E1 component subunit beta (EC 1.2.4.1)	DOWN(8)	-2,14345	0,00371
C1GLX6	cytochrome c oxidase subunit 4	DOWN(8)	-2,06261	0,003536
**Transport**				
C1GJS2	Phosphatidylinositol transfer protein SFH5 (PITP SFH5)	DOWN(D)	-7,21769	1,52E-05
C1GLM2	voltage-dependent anion channel protein 2	DOWN(8)	-6,26569	9,37E-05
C1GM08	GTP-binding protein ypt3	DOWN(D)	-5,74473	0,000857
C1GGE1	Mitochondrial thiamine pyrophosphate carrier 1	DOWN(8)	-5,40703	0,00025
A0A0A0HWM 5	mitochondrial import receptor subunit TOM70	DOWN(D)	-5,26458	0,000113
C1GID7	carnitine O-acetyltransferase [EC:2.3.1.7]	DOWN(D)	-5,21507	7,38E-05
C1GHF6	Mitochondrial thiamine pyrophosphate carrier 1	DOWN(D)	-5,13899	0,00027
C1GAA6	Mitochondrial phosphate carrier protein	DOWN(12)	-4,55762	0,007916
C1FZ88	importin subunit alpha-6/7	DOWN(8)	-4,36192	0,003272
C1GMC5	Mitochondrial thiamine pyrophosphate carrier 1	DOWN(8)	-4,08467	0,000575
C1GLH5	Mitochondrial GTP/GDP carrier protein 1	DOWN(8)	-3,97911	0,001008
C1GCH8	Mitochondrial pyruvate carrier	DOWN(8)	-3,67335	0,028458
C1GML4	Mitochondrial thiamine pyrophosphate carrier 1	DOWN(8)	-3,35598	0,024533
C1GAF5	Coatomer subunit alpha	DOWN(8)	-2,60008	0,001756

C1GHQ7	Endoplasmic reticulum transmembrane protein	DOWN(D)	-2,33778	0,006484
**Amino acid metabolism**				
C1GC81	Spermidine synthase	DOWN(8)	-7,07988	0,000537
C1GBD8	3-oxoacid CoA-transferase [EC:2.8.3.5]	DOWN(8)	-6,39235	5,76E-05
C1GCG7	Arginine-requiring protein 2	DOWN(8)	-6,15968	9,72E-05
C1GBT4	1-pyrroline-5-carboxylate dehydrogenase [EC:1.2.1.88]	DOWN(8)	-6,07164	0,00025
C1GJD3	isovaleryl-CoA dehydrogenase [EC:1.3.8.4]	DOWN(8)	-5,64098	0,00017
C1GC48	Branched-chain-amino-acid aminotransferase (EC 2.6.1.42)	DOWN(8)	-5,58633	0,000276
C1G3V5	Aspartate aminotransferase (EC 2.6.1.1)	DOWN(12)	-5,05193	7,6E-05
C1GGJ2	Ketol-acid reductoisomerase, mitochondrial (EC 1.1.1.86) (Acetohydroxy-acid reductoisomerase) (Alpha-keto-beta-hydroxylacyl reductoisomerase)	DOWN(D)	-4,20248	0,006783
C1G0L5	Choline dehydrogenase	DOWN(D)	-4,06077	0,000428
C1G1E5	hydroxymethylglutaryl-CoA synthase [EC:2.3.3.10]	DOWN(D)	-3,91866	4,87E-05
C1GLQ9	Ornithine transcarbamylase	DOWN(8)	-2,62091	0,029275
**Virulence factors**				
C1GKT9	Thioredoxin domain-containing protein	DOWN(8)	-6,48413	3,21E-05
C1GJI6	Subtilisin-like serine protease pepC	DOWN(D)	-5,95319	0,00015
A0A0A0HTP6	Alcohol dehydrogenase 1	DOWN(D)	-5,2512	3,72E-05
C1GJI2	Superoxide dismutase [Cu-Zn]	DOWN(D)	-3,5651	0,00071
C1G7K8	cytochrome c peroxidase [EC:1.11.1.5]	DOWN(D)	-3,10035	0,000272
**Other metabolisms**				
C1GKG3	NAD(P)H:quinone oxidoreductase, type IV	DOWN(8)	-7,07626	9,72E-05
C1GBT0	Aldehyde dehydrogenase	DOWN(8)	-7,03539	1,18E-05
C1GDE1	Serine hydroxymethyltransferase (EC 2.1.2.1)	DOWN(D)	-5,7886	0,000728
C1G1P0	Pribosyltran_N domain-containing protein	DOWN(8)	-4,62886	0,000217
C1GJM4	Vacuolar aminopeptidase I	DOWN(D)	-4,32296	0,000825
C1G3H9	Aldehyde dehydrogenase	DOWN(8)	-2,97889	0,000851
C1GG77	Carboxypeptidase Y homolog A (EC 3.4.16.5)	DOWN(D)	-2,92665	0,00822
C1GJT8	Nucleoside diphosphate kinase (EC 2.7.4.6)	DOWN(D)	-2,46476	0,013929
C1GHN0	Mitochondrial-processing peptidase subunit beta	DOWN(D)	-1,70344	0,000409
**Cytoskeleton**				
C1G6U5	Tubulin beta chain	DOWN(D)	-6,03394	0,000478
A0A0A0HRG7	Actin	DOWN(D)	-5,72342	3,37E-05

C1FZT8	tubulin alpha	DOWN(D)	-2,70129	0,001761
A0A0A0HWK8	Actin	DOWN(D)	-2,07246	0,004465
**Cell cycle**				
C1GB68	HIT domain-containing protein	DOWN(8)	-7,89176	3,21E-05
C1G4I1	Cell division cycle 20-like protein 1, cofactor-APC complex	DOWN(D)	-7,09341	1,16E-05
C1GCT8	GTP-binding nuclear protein Ran	DOWN(D)	-5,57243	5,03E-06
**Signal transduction**				
C1G501	Calmodulin	DOWN(8)	-5,68868	0,002507
**No annotation found**				
C1G2H2	Uncharacterized protein	DOWN(8)	-7,51093	0,000276
C1G6F9	Uncharacterized protein	DOWN(D)	-7,28525	4,24E-05
C1G486	Uncharacterized protein	DOWN(8)	-4,86583	0,000537
C1GFJ7	Uncharacterized protein	DOWN(8)	-4,80627	0,000731
C1G0Q6	NMO domain-containing protein	DOWN(D)	-4,00433	0,000768
C1G3V2	Uncharacterized protein	DOWN(8)	-3,42439	0,000831
