# Supplementary material for: Transcriptional profiling of a fungal granuloma reveals a low metabolic activity of Paracoccidioides brasiliensis yeasts and an actively regulated host immune response
Source: Front Cell Infect Microbiol. 2023 Oct 5;13:1268959. doi: 10.3389/fcimb.2023.1268959 (PMC10585178; doi:10.3389/fcimb.2023.1268959)
Supplement: Supplementary file 11 [file Table_10.pdf]

Supplementary Table 10. Yeast proteins absent in the infection.

Acession number	Protein
**Gene/protein regulation**	
A0A0A0HQI3	Nucleoporin NDC1
A0A0A0HQN9	40S ribosomal protein S19
A0A0A0HR72	Sorting nexin-41
A0A0A0HSI5	UBA domain-containing protein
A0A0A0HSJ2	Nuclear pore complex component-domain-containing protein
A0A0A0HSS2	DNA polymerase epsilon subunit Dpb3
A0A0A0HT52	GTP cyclohydrolase
A0A0A0HTI2	VWFA domain-containing protein
A0A0A0HTN8	Acyl-protein thioesterase
A0A0A0HTQ5	Protein-serine/threonine kinase
A0A0A0HTY8	nucleolar protein 58
A0A0A0HU09	Poly(RC)-binding protein
A0A0A0HU55	Splicing factor 3A subunit 3
A0A0A0HUA0	Ubiquitin interaction motif protein
A0A0A0HUF3	U6 snRNA-associated Sm-like protein LSm3
A0A0A0HUI8	Histone H2A.Z
A0A0A0HUJ5	large subunit ribosomal protein L3e
A0A0A0HUV5	DNA helicase
A0A0A0HV09	ATP-dependent RNA helicase eIF4A
A0A0A0HV62	Ribosomal protein L19
A0A0A0HV65	Ubiquitin-conjugating enzyme variant MMS2
A0A0A0HV97	Transcription initiation factor TFIID/TFIIF subunit
A0A0A0HVN3	ATP-dependent RNA helicase DDX5/DBP2 [EC:3.6.4.13]
A0A0A0HW16	DNA-directed RNA polymerase II 138 kDa polypeptide
A0A0A0HWB1	E3 ubiquitin-protein ligase
A0A0A0HX32	mRNA decapping complex subunit Dcp2
A0A0A0HX33	actin-related protein 8
A0A0A0HYT2	Signal recognition particle subunit SRP72
A0A0A0HZA1	Exonuclease domain-containing protein
C1FYE5	glutamyl-tRNA synthetase [EC:6.1.1.17]
C1FYE8	HABP4_PAI-RBP1 domain-containing protein
C1FYH2	DNA-directed RNA polymerases I, II, and III subunit RPABC1
C1FYI5	U4/U6 small nuclear ribonucleoprotein PRP3
C1FYJ6;C1GIP8	Histone H4
C1FYP9	PARP-type domain-containing protein
C1FYQ2	T-complex protein 1 subunit gamma
C1FYQ6	protein TIF31
C1FYR6	40S ribosomal protein S7
C1FZ00	large subunit ribosomal protein L37Ae
C1FZ06	NA
C1FZ53	Translation initiation factor 4B
C1FZ57	large subunit ribosomal protein L32e
C1FZ84	Translocon-associated protein

C1FZC5	RNA_pol_L_2 domain-containing protein
C1FZD4	Eukaryotic translation initiation factor 3 subunit M
C1FZG1	Peptidyl-prolyl cis-trans isomerase
C1FZI7	Vacuolar protein sorting/targeting protein 10
C1FZK4	28 kDa ribonucleoprotein
C1FZL0	T-complex protein 1 subunit delta
C1FZL1	26S proteasome regulatory subunit T1
C1FZM4	tRNA (Guanine37-N1)-methyltransferase
C1FZM7	Polymerase II polypeptide D
C1FZN1	26S proteasome regulatory subunit RPN10
C1FZP0	Eukaryotic translation initiation factor 3 subunit D
C1G017	Zuotin
C1G0A3	Nuclear pore protein
C1G0G4	40S ribosomal protein S17
C1G0Q9	U1 snRNP-associated protein Usp107
C1G0S6	Mitochondrial genome maintenance protein MGM101
C1G109	Dolichol-phosphate mannosyltransferase subunit 1
C1G128	General negative regulator of transcription subunit 1
C1G136	Pre-RNA splicing factor Srp2, variant 2
C1G162	Protein arginine N-methyltransferase 1
C1G172	large subunit ribosomal protein L27e
C1G175	Proteasome subunit alpha type
C1G179	Transcription factor atf1
C1G1D6	Rnapii degradation factor def1
C1G1H8	histone chaperone ASF1
C1G1M5	ATP-dependent Clp protease ATP-binding subunit ClpB
C1G1N8	Hypoxia up-regulated 1
C1G1R9	RNase H type-1 domain-containing protein
C1G231	ThiF domain-containing protein
C1G240	DnaJ domain protein Psi
C1G2D0	Eukaryotic translation initiation factor 3 subunit F
C1G2D4	Protein phosphatase
C1G2F2	Rhomboid domain-containing protein
C1G2H3	mRNA export factor
C1G2I0	mRNA stability protein
C1G2I1	Carboxypeptidase D
C1G2S5	General negative regulator of transcription subunit 2
C1G2V1	30S ribosomal protein S17P
C1G2Y0	Methionine aminopeptidase
C1G349	protein disulfide-isomerase [EC:5.3.4.1]
C1G373	translation initiation factor 3 subunit C
C1G390	phenylalanyl-tRNA synthetase beta chain [EC:6.1.1.20]
C1G391	small subunit ribosomal protein S3e
C1G3I3	ISWI chromatin-remodeling complex ATPase ISW1
C1G3L4	Guanine nucleotide-binding protein subunit beta-like protein
C1G3X6	Iron-sulfur cluster assembly protein

C1G3Y0	RuvB-like helicase
C1G3Y6	E3 ubiquitin-protein ligase HUWE1
C1G3Y8	40S ribosomal protein S6
C1G412	Peptidase S59 domain-containing protein
C1G445	Hsp90 co-chaperone AHA1
C1G481	Proteasome-activating nucleotidase
C1G497	Chromatin modification-related protein
C1G4B4	Zn(2)-C6 fungal-type domain-containing protein
C1G4C0	N-alpha-acetyltransferase 15/16, NatA auxiliary subunit
C1G4I3	obg-like ATPase 1
C1G4I4	Translation machinery-associated protein 20
C1G4M6	Pre-mRNA-processing factor 40
C1G4S4	Transcription factor iws1
C1G4S9	Protein SEY1
C1G4T2	BZIP domain-containing protein
C1G4U9	Dynamin GTPase
C1G4X6	Mitochondrial-processing peptidase subunit alpha
C1G514	Hsp90 chaperone protein kinase-targeting subunit
C1G540	large subunit ribosomal protein L7/L12
C1G541	T-complex protein 1 subunit theta
C1G554	ubiquitin-activating enzyme E1 [EC:6.2.1.45]
C1G561	aminoacyl tRNA synthase complex-interacting multifunctional protein 1
C1G570	prolyl-tRNA synthetase [EC:6.1.1.15]
C1G571	T-complex protein 1 subunit alpha
C1G582	DnaJ domain-containing protein
C1G5E9	Peptidylprolyl isomerase
C1G5F5	Histone deacetylase
C1G5J1	60S acidic ribosomal protein P2
C1G5M9	valyl-tRNA synthetase [EC:6.1.1.9]
C1G5P6	PWWP domain-containing protein
C1G5Q5	U4/U6 small nuclear ribonucleoprotein PRP4
C1G5U7	Endoplasmic oxidoreductin-1
C1G5V0	Nucleic acid-binding protein
C1G5V8	Signal peptidase complex subunit 2
C1G5Y3	Protein phosphatase PP2A regulatory subunit B
C1G5Z1	Transcriptional repressor Sin3p
C1G631	26S proteasome regulatory subunit T2
C1G632	ubiquitin-conjugating enzyme (huntingtin interacting protein 2) [EC:2.3.2.23]
C1G651	Vacuolar protein sorting-associated protein 17
C1G675	Anucleate primary sterigmata protein A
C1G686	translation initiation factor 6
C1G696	Ferrochelatase
C1G6A7	Decapping enzyme Dcp1
C1G6D0	Proteasome subunit alpha type
C1G6E7	116 kDa U5 small nuclear ribonucleoprotein component

C1G6F6	heat shock 70kDa protein 1/2/6/8
C1G6M3	Ribosomal protein
C1G6R0	protein SSD1
C1G6R9	protein mago nashi
C1G6T3	60S ribosomal protein L6
C1G6U0	ATP-dependent Clp protease ATP-binding subunit ClpB
C1G6U1	Elongation factor 1-beta
C1G6W9	protein LSM14
C1G741	LsmAD domain-containing protein
C1G746	N-acetyltransferase domain-containing protein
C1G763	RNA-binding domain-containing protein
C1G7B9	transcription elongation factor SPT5
C1G7C1	26S proteasome regulatory subunit N2
C1G7F3	Piwi domain-containing protein
C1G7H6	RNA polymerase II transcription factor B subunit 3
C1G7J2	Pre-mRNA-processing factor 39
C1G7J3	RNA helicase
C1G7N7	Proteasome component PUP2
C1G7T3	Chaperone DnaJ
C1G7X8	RNA helicase
C1G810	40S ribosomal protein S4
C1G820	60S ribosomal protein L21-A
C1G821	40S ribosomal protein S9
C1G892	DNA-directed RNA polymerase
C1G8C2	Peptide-methionine (R)-S-oxide reductase
C1G8E5	peptide chain release factor subunit 3
C1G8K9	Xaa-Pro aminopeptidase
C1G8Q6	Tryptophan--tRNA ligase
C1G8T3	mitochondrial protein import protein ZIM17
C1G8T6	CwfJ domain-containing protein
C1G8U4	Proteasome subunit beta
C1G8V3	tyrosyl-tRNA synthetase [EC:6.1.1.1]
C1G8W2	Mago-bind domain-containing protein
C1G8X9	Peptidylprolyl isomerase
C1G8Y5	Mitochondrial intermediate peptidase
C1G8Z2	Nuclear pore complex subunit Nup159
C1G918	Transcription factor RfeD
C1G945	large subunit ribosomal protein L30e
C1G957	Dolichyl-diphosphooligosaccharide--protein glycosyltransferase subunit 1
C1G9A3	Uracil-DNA glycosylase
C1G9A5	protein disulfide-isomerase A1 [EC:5.3.4.1]
C1G9D3	HMG box domain-containing protein
C1G9N1	20S proteasome subunit beta 6
C1G9P6	PROTEASOME_ALPHA_1 domain-containing protein
C1G9T0	translation initiation factor 3 subunit A
C1G9U4	60S acidic ribosomal protein P0

C1G9U8	Chaperone DnaJ
C1G9X0	DNA damage checkpoint protein rad24
C1G9X1	Eukaryotic translation initiation factor 3 subunit J
C1G9X6	Probable carboxypeptidase
C1G9X9	large subunit ribosomal protein L36e
C1G9Z7	translation initiation factor 2 subunit 3
C1GA10	ATP-dependent RNA helicase DDX3X [EC:3.6.4.13]
C1GA39	mitochondrial presequence protease
C1GA62	alpha-mannosidase [EC:3.2.1.24]
C1GA83	ATP-dependent Clp protease, protease subunit [EC:3.4.21.92]
C1GA86	Replication factor C subunit 2
C1GAC4	Translation initiation factor 2 subunit beta
C1GAD6	Multiprotein-bridging factor 1
C1GAE8	26S proteasome non-ATPase regulatory subunit 8
C1GAF6	mitochondrial FAD-linked sulfhydryl oxidase [EC:1.8.3.2]
C1GAF7	Transcriptional regulator
C1GAG0	tRNA (Guanine9-N1)-methyltransferase
C1GAG5	translation initiation factor 3 subunit I
C1GAK2	Endoribonuclease L-PSP
C1GAL5	Prp8 binding protein
C1GAM9	40S ribosomal protein S24
C1GAP0	Protein FYV10
C1GAQ4	DNA-directed RNA polymerase II subunit RPB2 [EC:2.7.7.6]
C1GAT3	splicing factor U2AF 35 kDa subunit
C1GB14	Histone deacetylase
C1GB62	leucyl-tRNA synthetase [EC:6.1.1.4]
C1GB65	40S ribosomal protein S10-A
C1GB82	protein transport protein SEC24
C1GBA2	Mitochondrial RNA splicing protein
C1GBC4	mannosyl-oligosaccharide alpha-1,2-mannosidase [EC:3.2.1.113]
C1GBC8	WD repeat-containing protein
C1GBE8	Threonine-tRNA ligase
C1GBG1	aspartyl-tRNA synthetase [EC:6.1.1.12]
C1GBI7	NEDD8-activating enzyme E1 regulatory subunit
C1GBL0	Peptidyl-prolyl cis-trans isomerase
C1GBL7	E3 ubiquitin-protein ligase PEP5
C1GBM4	large subunit ribosomal protein L26e
C1GBN3	RRM domain-containing protein
C1GBT6	RRM domain-containing protein
C1GC24	nucleolar protein 15
C1GC90	Ribonucleoprotein
C1GCF0	ATP-dependent RNA helicase SUB2
C1GCK8	Nascent polypeptide-associated complex subunit alpha
C1GCS3	Deubiquitination-protection protein dph1
C1GCV8	T-complex protein 1 subunit zeta
C1GCV9	Nucleolar protein 58

C1GCZ3	UDP-glucose:glycoprotein glucosyltransferase [EC:2.4.1.-]
C1GD28	protein transport protein SEC13
C1GD93	Chaperone protein dnaJ 3
C1GDH2	Type 2A phosphatase activator tip41
C1GDI5	Endonuclease
C1GDP1	RNA binding protein Jsn1
C1GDQ2	Asparagine--tRNA ligase
C1GDR3	Pre-mRNA-splicing factor cef1
C1GDR8	Deoxyhypusine hydroxylase
C1GDT4	DNA topoisomerase 2-associated protein PAT1
C1GE31	Rho GDP-dissociation inhibitor
C1GE59	RNA helicase
C1GE74	26S proteasome regulatory subunit RPN1
C1GEA1	U2 small nuclear ribonucleoprotein B
C1GEC6	Alpha-1,2 mannosyltransferase KTR1
C1GEF6	SRP9-21 domain-containing protein
C1GEF7	Ribonucleoside-diphosphate reductase small chain
C1GEL8	Cullin-associated NEDD8-dissociated protein
C1GEM3	Histone acetyltransferase type B subunit 2
C1GEN2	DnaJ domain-containing protein
C1GEN5	large subunit ribosomal protein L4e
C1GEQ8	Alpha-1,2 mannosyltransferase KTR1
C1GET9	DNA replication licensing factor MCM5
C1GF01	E3 ubiquitin ligase complex SCF subunit
C1GF12	glycyl-tRNA synthetase [EC:6.1.1.14]
C1GF13	Glycyl-tRNA synthetase
C1GF57	nucleosome assembly protein 1-like 1
C1GF61	seryl-tRNA synthetase [EC:6.1.1.11]
C1GF88	RNA helicase
C1GF93	Protein-L-isoaspartate O-methyltransferase
C1GF97	Ubiquitin thioesterase OTU1
C1GFA3	60S ribosomal protein L27a
C1GFE3	Transcription elongation factor Spt6
C1GFG5	Histone H1
C1GFH3	Flap endonuclease 1
C1GFI8	translation initiation factor 2A
C1GFJ3	Transcription initiation factor TFIID subunit 12
C1GFQ8	SURF1-like protein
C1GFS4	Translation machinery-associated protein 22
C1GFU5	EKC/KEOPS complex subunit BUD32
C1GFX4	translation initiation factor 2 subunit 1
C1GGD8	Ubiquitin-conjugating enzyme
C1GGR5	small subunit ribosomal protein S20e
C1GGT8	40S ribosomal protein S1
C1GH39	TAF4 domain-containing protein
C1GH47	tRNA pseudouridine13 synthase

C1GH63	40S ribosomal protein S25
C1GHE3	Transcription factor C subunit 7
C1GHL1	DNA-directed RNA polymerase subunit
C1GHT1	Translocation protein sec63
C1GHV2	40S ribosomal protein S5
C1GHW5	26S proteasome non-ATPase regulatory subunit 11
C1GI59	GYF domain-containing protein
C1GIH5	Replication factor C subunit 1
C1GIH8	Mitochondrial intermembrane space import and assembly protein 40
C1GIL9	arginyl-tRNA synthetase [EC:6.1.1.19]
C1GIQ4	HMG box domain-containing protein
C1GIS6	U6 snRNA-associated Sm-like protein LSm4
C1GIV4	Proteasome subunit alpha type
C1GJ49	RRM domain-containing protein
C1GJ94	tRNA threonylcarbamoyladenosine dehydratase
C1GJI0	Probable endonuclease LCL3
C1GJJ6	chloride channel, nucleotide-sensitive, 1A
C1GJL2	cysteinyl-tRNA synthetase [EC:6.1.1.16]
C1GJS9	UBX domain-containing protein
C1GJX2	Ubiquitinyl hydrolase 1
C1GJZ6	Phenylalanine--tRNA ligase
C1GK43	C2H2-type domain-containing protein
C1GK54	Pre-mRNA-splicing factor ATP-dependent RNA helicase PRP16
C1GK70	NEDD8-activating enzyme E1 catalytic subunit
C1GK84	SAP domain-containing protein
C1GK92	Splicing factor U2AF subunit
C1GKA3	RRM domain-containing protein
C1GKF9	mRNA 3'-end-processing protein yth1
C1GKG6	Protein phosphatase methylesterase 1
C1GKL8	protein transport protein SEC23
C1GKM9	protein disulfide-isomerase A6 [EC:5.3.4.1]
C1GKR7	large subunit ribosomal protein L24e
C1GKU6	Peptidyl-prolyl cis-trans isomerase-like 1
C1GKX0	Eukaryotic translation initiation factor 4C
C1GL26	translation initiation factor 3 subunit B
C1GL41	T-complex protein 1 subunit beta
C1GL49	replication factor A1
C1GL60	Protein MPE1
C1GL98	polyadenylate-binding protein
C1GLA2	Eukaryotic translation initiation factor 5A
C1GLA9	translation initiation factor 5B
C1GLB5	DNA replication licensing factor MCM3
C1GLC7	transcriptional activator HAP5
C1GLD3	FACT complex subunit SPT16
C1GLD7	dolichyl-phosphate-mannose-protein mannosyltransferase [EC:2.4.1.109]
C1GLF9	26S proteasome regulatory subunit rpn-8

C1GLI8	CCCH zinc finger and RRM domain-containing protein
C1GLP2	SAP domain-containing protein
C1GLR4	KH domain-containing protein
C1GLS6	Ubiquitin conjugation factor E4 B
C1GLV0	Histone chaperone
C1GM02	Mitochondrial DNA replication protein YHM2
C1GM32	RuvB-like helicase 2
C1GMI6	lysyl-tRNA synthetase, class II [EC:6.1.1.6]
C1GMJ7	Heterochromatin protein HP1
C1GMJ8	T-complex protein 1 subunit epsilon
C1GMR5	Peroxin-1
C1GMU3	Peptidylprolyl isomerase
C1GMV5	DnaJ domain protein
C1GMV8	40S ribosomal protein S2
C1GMZ9	ATPase family AAA domain-containing protein 1-B
C1GN19	40S ribosomal protein S28
C1GN32	RNA binding effector protein Scp160
C1GN40	Chromatin assembly factor 1 subunit B
C1GN69	Transcription factor BYE1
**Amino acid metabolism**	
A0A0A0HQV9	Homoserine O-acetyltransferase
A0A0A0HRQ4	glutaryl-CoA dehydrogenase [EC:1.3.8.6]
A0A0A0HS25	DAO domain-containing protein
A0A0A0HTR8	omega-amidase [EC:3.5.1.3]
C1FYE6	Glutaminase A
C1FZL3	dipeptidyl aminopeptidase B [EC:3.4.14.-]
C1G025	Aromatic-L-amino-acid decarboxylase
C1G032	DAO domain-containing protein
C1G0F9	kynureninase [EC:3.7.1.3]
C1G0J6	Phospho-2-dehydro-3-deoxyheptonate aldolase
C1G140	DAO domain-containing protein
C1G197	Arginase
C1G297	1-(5-phosphoribosyl)-5-[(5-phosphoribosylamino)methylideneamino] imidazole-4-carboxamide isomerase
C1G2Y9	Argininosuccinate lyase
C1G312	ornithine--oxo-acid transaminase [EC:2.6.1.13]
C1G388	Aspartate aminotransferase
C1G3B0	Galactonate dehydratase
C1G3G3	gamma-glutamyltranspeptidase / glutathione hydrolase [EC:2.3.2.2 3.4.19.13]
C1G3U9	Homocitrate synthase
C1G452	saccharopine dehydrogenase (NADP+, L-glutamate forming) [EC:1.5.1.10]
C1G4C3	Anthranilate phosphoribosyltransferase
C1G4J8	Kynurenine formamidase
C1G4R2	S-adenosylmethionine synthetase [EC:2.5.1.6]
C1G4U7	Glycine cleavage system H protein

C1G553	GST N-terminal domain-containing protein
C1G596	3-hydroxyisobutyrate/3-hydroxypropionate dehydrogenase [EC:1.1.1.31 1.1.1.59]
C1G5K1	Cystathionine gamma-lyase
C1G5P3	3-hydroxyanthranilate 3,4-dioxygenase
C1G6B4	Methionine adenosyltransferase 2 subunit beta
C1G6C1	Cysteine synthase
C1G6H2	threonine synthase [EC:4.2.3.1]
C1G6P2	2,4-dihydroxyhept-2-ene-1,7-dioic acid aldolase
C1G6V9	aminomethyltransferase [EC:2.1.2.10]
C1G765	Alanine--glyoxylate transaminase
C1G792	threonine dehydratase [EC:4.3.1.19]
C1G7A3	succinate-semialdehyde dehydrogenase / glutarate-semialdehyde dehydrogenase [EC:1.2.1.16 1.2.1.79 1.2.1.20]
C1G7L2	3-isopropylmalate dehydratase [EC:4.2.1.33]
C1G8C8	2-oxoisovalerate dehydrogenase E1 component alpha subunit [EC:1.2.4.4]
C1G8D6	S-methyl-5'-thioadenosine phosphorylase
C1G8P1	2-oxoisovalerate dehydrogenase E1 component beta subunit [EC:1.2.4.4]
C1G8V0	aspartate aminotransferase, cytoplasmic [EC:2.6.1.1]
C1G8Z9	Proline iminopeptidase
C1G900	Tyrosine decarboxylase
C1G949	ATP phosphoribosyltransferase
C1GAY3	proline dehydrogenase [EC:1.5.5.2]
C1GB53	anthranilate synthase component I [EC:4.1.3.27]
C1GC00	Homoserine kinase
C1GCG4	Glutamine synthetase
C1GCK1	Betaine aldehyde dehydrogenase
C1GD55	5-oxoprolinase (ATP-hydrolysing) [EC:3.5.2.9]
C1GD67	Peptidyl-prolyl cis-trans isomerase
C1GDG5	cystathionine beta-synthase [EC:4.2.1.22]
C1GDK1	Glutamate-5-semialdehyde dehydrogenase
C1GEZ1	Arginine biosynthesis bifunctional protein ArgJ, mitochondrial
C1GF60	Phosphoglycerate dehydrogenase
C1GF68	Dihydrolipoyllysine-residue succinyltransferase
C1GF82	Cystathionine gamma-synthase
C1GF86	Cys-Gly metallodipeptidase DUG1 [EC:3.4.13.-]
C1GFM4	Phosphoserine phosphatase
C1GFV8	Saccharopine dehydrogenase [NAD(+), L-lysine-forming]
C1GG15	1,2-dihydroxy-3-keto-5-methylthiopentene dioxygenase
C1GG64	Imidazoleglycerol-phosphate dehydratase
C1GG82	Glutamate decarboxylase
C1GGZ4	Phosphoserine transaminase
C1GGZ6	Homoaconitase, mitochondrial
C1GHU8	2-isopropylmalate synthase
C1GHZ0	3-hydroxyisobutyryl-CoA hydrolase [EC:3.1.2.4]

C1GI69	tryptophan synthase [EC:4.2.1.20]
C1GIR0	Pyrroline-5-carboxylate reductase
C1GJ05	dihydroxy-acid dehydratase [EC:4.2.1.9]
C1GJ38	anthranilate synthase / indole-3-glycerol phosphate synthase / phosphoribosylanthranilate isomerase [EC:4.1.3.27 4.1.1.48 5.3.1.24]
C1GJD0	3-methylcrotonyl-CoA carboxylase alpha subunit [EC:6.4.1.4]
C1GK23	Dihydroxy-acid dehydratase
C1GK60	Aromatic amino acid aminotransferase
C1GL14	Phospho-2-dehydro-3-deoxyheptonate aldolase
C1GL38	Branched-chain amino acid aminotransferase
C1GL59	Imidazole glycerol phosphate synthase hisHF
C1GLF6	L-2-aminoadipate reductase [EC:1.2.1.95]
C1GLK1	Aconitate hydratase, mitochondrial
C1GLM1	asparagine synthase (glutamine-hydrolysing) [EC:6.3.5.4]
C1GLR3	Acetohydroxy-acid synthase small subunit
C1GLY5	Aspartate-semialdehyde dehydrogenase
C1GMH9	Fumarylacetoacetase
C1GMI0	Homogentisate 1,2-dioxygenase
C1GMI2	4-hydroxyphenylpyruvate dioxygenase [EC:1.13.11.27]
C1GN02	Cystathionine beta-lyase
C1GN18	ATP phosphoribosyltransferase
**Energy metabolism**	
A0A0A0HRM2	V-type H+-transporting ATPase subunit A [EC:7.1.2.2]
A0A0A0HUI2	NADH dehydrogenase [ubiquinone] 1 alpha subcomplex assembly factor 3
A0A0A0HVE7	NADH-ubiquinone oxidoreductase
A0A0A0HVK7	NADPH--cytochrome P450 reductase
A0A0A0HVL2	Long-chain-fatty-acid-CoA ligase
A0A0A0HWQ9	NADH-ubiquinone oxidoreductase 78 kDa subunit, mitochondria
A0A0A0HX82	phosphoglucomutase [EC:5.4.2.2]
C1FYU2	Mitochondrial protein Fmp25
C1FYW8	chitinase [EC:3.2.1.14]
C1FZ35	Cyanate hydratase
C1FZV1	ribulose-phosphate 3-epimerase [EC:5.1.3.1]
C1G002	6-phosphofructokinase 1 [EC:2.7.1.11]
C1G0R1	Glucose-6-phosphate isomerase
C1G0V2	NADH-ubiquinone oxidoreductase kD subunit
C1G137	electron-transferring-flavoprotein dehydrogenase [EC:1.5.5.1]
C1G1H4	pyruvate decarboxylase [EC:4.1.1.1]
C1G1T5	Glutamate synthase (NADH)
C1G256	NADH dehydrogenase [ubiquinone] 1 alpha subcomplex subunit
C1G278	acyl-CoA dehydrogenase [EC:1.3.8.7]
C1G350	NADH dehydrogenase (Ubiquinone) 1 alpha subcomplex 5
C1G3P1	Ubiquinone biosynthesis monooxygenase COQ6, mitochondrial
C1G437	V-type proton ATPase subunit a
C1G440	fructose-1,6-bisphosphatase I [EC:3.1.3.11]
C1G496	Oxoglutarate dehydrogenase (succinyl-transferring)

C1G4R7	V-type proton ATPase subunit H
C1G5C9	NADH dehydrogenase [ubiquinone] 1 beta subcomplex subunit 7
C1G5Z3	Beta-glucosidase
C1G658	Homoserine O-acetyltransferase
C1G6C3	sulfite oxidase [EC:1.8.3.1]
C1G6E6	acetyl-CoA C-acetyltransferase [EC:2.3.1.9]
C1G6V7	V-type proton ATPase subunit C
C1G794	Respiratory supercomplex factor 1, mitochondrial
C1G7G3	Glucose-6-phosphate 1-epimerase
C1G7N9	acetyl-CoA acyltransferase 1 [EC:2.3.1.16]
C1G890	sulfite reductase (NADPH) hemoprotein beta-component [EC:1.8.1.2]
C1G8D0	NADH-ubiquinone oxidoreductase 30.4 kDa subunit, mitochondrial
C1G8L6	Cytochrome b5 heme-binding domain-containing protein
C1G8R5	6-phosphogluconate dehydrogenase [EC:1.1.1.44 1.1.1.343]
C1G950	NADH-ubiquinone oxidoreductase 24 kDa subunit
C1G977	Hexokinase
C1G9B6	Nfu_N domain-containing protein
C1GA79	Pyruvate dehydrogenase complex component Pdx1
C1GA89	inorganic pyrophosphatase [EC:3.6.1.1]
C1GAJ0	ATP synthase mitochondrial F1 complex assembly factor 2
C1GAJ4	Quinone oxidoreductase
C1GB77	NADH-ubiquinone oxidoreductase 40 kDa subunit, mitochondrial
C1GBI8	Ribose-5-phosphate isomerase
C1GBX3	oxalate---CoA ligase [EC:6.2.1.8]
C1GC37	Cytochrome b5 heme-binding domain-containing protein
C1GCK7	ATP synthase subunit d, mitochondrial
C1GCW1	Cytochrome c oxidase assembly protein COX20, mitochondrial
C1GCX3	2,3-bisphosphoglycerate-independent phosphoglycerate mutase [EC:5.4.2.12]
C1GDJ9	Iron sulfur cluster assembly protein 1, mitochondrial
C1GF69	NADH dehydrogenase [ubiquinone] 1 beta subcomplex subunit 9
C1GFQ2	Ubiquinone biosynthesis protein COQ4, mitochondrial
C1GGI8	Delta(3,5)-Delta(2,4)-dienoyl-CoA isomerase
C1GHV7	V-type proton ATPase subunit
C1GI20	triosephosphate isomerase (TIM) [EC:5.3.1.1]
C1GI70	Vacuolar proton pump subunit B
C1GIJ5	Probable electron transfer flavoprotein subunit alpha
C1GJQ8	2Fe-2S ferredoxin-type domain-containing protein
C1GK68	NADH-ubiquinone oxidoreductase 49 kDa subunit, mitochondrial
C1GKM7	ATP synthase subunit gamma
C1GKQ0	Pyruvate decarboxylase
C1GKV7	Vacuolar ATP synthase subunit E
C1GL06	Succinate dehydrogenase [ubiquinone] iron-sulfur subunit, mitochondrial
C1GL12	Glycogen debranching enzyme
C1GLI3	fumarate hydratase, class II [EC:4.2.1.2]
C1GLP3;A0A0A0HSX1	Cytochrome b2

C1GMD0	NADH dehydrogenase (ubiquinone) Fe-S protein 8 [EC:7.1.1.2]
Q1XAA6	NADH-ubiquinone oxidoreductase chain 5
**Cell cycle**	
A0A0A0HUN4	Mitotic checkpoint protein BUB3
A0A0A0HWU6	Tubulin gamma chain
C1FZX8	Protein kinase dsk1
C1G389	serine/threonine-protein phosphatase 2A regulatory subunit A
C1G402	Cell division control protein Cdc31
C1G681	Reduced viability upon starvation protein
C1G6F8	Cyclin-dependent kinases regulatory subunit
C1G6J0	Protein stu1
C1G788	Restriction of telomere capping protein 5
C1G7B8	Nuclear movement protein nudC
C1G7R4	Myosin regulatory light chain cdc4
C1G857	Cohesin complex subunit SA-1/2
C1G861	Rab-GAP TBC domain-containing protein
C1G8I3	Autophagy-related protein 13
C1G994	ANAPC4_WD40 domain-containing protein
C1GBL1	Zinc finger protein zpr1
C1GCC3	Serine/threonine-protein kinase sid2
C1GCN4	Pheromone-dependent cell cycle arrest protein Far11
C1GEM8	cell division control protein 12
C1GEQ3	NAP family protein
C1GFC0	Anucleate primary sterigmata protein ApsB
C1GG95	Pro-apoptotic serine protease NMA111
C1GIL6	Structural maintenance of chromosomes protein
C1GJ83	cell division control protein 11
C1GL30	Velvet domain-containing protein
C1GLF0	Centromere protein C
C1GMD9	Protein SDS23
**Transport**	
A0A0A0HT88	Long-chain fatty acid transporter
A0A0A0HUB3	Oxysterol binding protein (Osh1)
A0A0A0HVG2	Importin N-terminal domain-containing protein
A0A0A0HVN6	Microtubule binding protein HOOK3
A0A0A0HWB7	Mitochondrial import inner membrane translocase subunit tim23
A0A0A0HWS3	vacuolar protein sorting-associated protein 1
A0A0A0HWW6	K(+)/H(+) antiporter 1
A0A0A0HXV1	AP complex subunit beta
C1FYF2	Peroxin-19
C1FYK9	MHD domain-containing protein
C1FYP6	Bud site selection protein
C1FYZ4	Mitochondrial thiamine pyrophosphate carrier 1
C1FZ16	60S ribosomal export protein NMD3
C1FZH1	Actin binding protein
C1FZH6	dynamin 1-like protein [EC:3.6.5.5]

C1FZK1	Ran GTPase-activating protein
C1FZY5	Plasma membrane channel protein
C1G0A9	Calcium/proton exchanger
C1G0N4	Importin N-terminal domain-containing protein
C1G112	Vacuolar protein sorting-associated protein 35
C1G1C6	DUF221 domain-containing protein
C1G1D9	MFS domain-containing protein
C1G1M0	Autophagy-related protein 27
C1G207	MFS domain-containing protein
C1G226	Autophagy-related protein 3
C1G2M5	Nuclear protein export protein Yrb2
C1G2Y7	ABC transporter domain-containing protein
C1G351	Coatomer subunit beta
C1G3G2	Mitochondrial import inner membrane translocase subunit tim14
C1G3N9	Sorting nexin-4
C1G3Q1	Sorting nexin 3
C1G3T6	P-type Na+/K+ transporter [EC:7.2.2.3 7.2.2.-]
C1G4F7	Mitochondrial thiamine pyrophosphate carrier 1
C1G4I7	Mitochondrial inner membrane translocase subunit TIM44
C1G4R8	M protein repeat protein
C1G584	AA_permease domain-containing protein
C1G5H2	Probable lysosomal cobalamin transporter
C1G5L4	dynein cytoplasmic 1 intermediate chain
C1G635	ATP-binding cassette, subfamily F, member 2
C1G6S0	Coatomer subunit beta
C1G6Y4	carnitine O-acetyltransferase [EC:2.3.1.7]
C1G764	Vacuolar protein sorting-associated protein 29
C1G784	MICOS complex subunit MIC60
C1G7G9	MFS transporter, SP family, sugar:H+ symporter
C1G7U5	ATP-dependent permease PDR12
C1G7W6	MFS domain-containing protein
C1G812	Sorting nexin-3
C1G8F9	SSD domain-containing protein
C1G8G0	importin-4
C1G8H8	Vacuolar protein sorting-associated protein 27
C1G8S5	AA_permease domain-containing protein
C1G9E6	mitochondrial import inner membrane translocase subunit TIM10
C1G9U1	COPII-coated vesicle component Erv46
C1G9W2	Small COPII coat GTPase SAR1
C1G9W9	Calcium-transporting ATPase
C1GAU7	Transmembrane protein
C1GB16	mitochondrial import inner membrane translocase subunit TIM50
C1GBG4	coatomer subunit gamma
C1GBP4	MFS domain-containing protein
C1GCX1	Vesicle-fusing ATPase
C1GDK3	Mitochondrial thiamine pyrophosphate carrier 1

C1GE21	DUF1681 domain-containing protein
C1GE52	actin-related protein 3
C1GE93	Solute carrier family 25 (Mitochondrial S-adenosylmethionine transporter), member 26
C1GER2	MFS domain-containing protein
C1GEX3	ATP-binding cassette, subfamily C (CFTR/MRP), member 1 [EC:7.6.2.3]
C1GF89	Mitochondrial thiamine pyrophosphate carrier 1
C1GG11	Vacuolar protein sorting-associated protein
C1GGB5	Calcium-binding protein NCS-1
C1GGB6	Transmembrane 9 superfamily member
C1GGE8	Kinesin-like protein
C1GGX1	ABC transporter domain-containing protein
C1GGX4	DUF410 domain-containing protein
C1GH26	FYVE-type domain-containing protein
C1GHB9	Coatomer subunit delta
C1GHD6	Potassium/sodium efflux P-type ATPase, fungal-type
C1GHE5	importin subunit beta-1
C1GHP1	Protein CASP
C1GHP2	CNH domain-containing protein
C1GI05	Probable vacuolar protein sorting-associated protein 16 homolog
C1GI19	Vacuolar protein 8
C1GIA8	Mitochondrial dicarboxylate transporter
C1GIB2	protein transport protein SEC31
C1GIX9	SEC7 domain-containing protein
C1GJH7	ER membrane protein complex subunit 1
C1GJN2	AP-1 complex subunit mu
C1GJQ7	Mitochondrial fusion protein
C1GK04	ENTH domain-containing protein
C1GK73	t-SNARE coiled-coil homology domain-containing protein
C1GKA9	Mitochondrial thiamine pyrophosphate carrier 1
C1GLH9	V-SNARE
C1GLJ9	Importin N-terminal domain-containing protein
C1GLP7	t-SNARE coiled-coil homology domain-containing protein
C1GLV2	solute carrier family 25 (peroxisomal adenine nucleotide transporter), member 17
C1GM90	Phosphatidylinositol-phosphatidylcholine transfer protein
C1GMA2	Vacuolar-sorting protein snf7
C1GMS5	Thiosulfate sulfurtransferase
C1GMU5	t-SNARE coiled-coil homology domain-containing protein
C1GN84	FAD-binding FR-type domain-containing protein
**Lipid metabolism**	
C1FZU0	Sterol esterase
C1G064	fatty acid synthase subunit alpha, fungi type [EC:2.3.1.86]
C1G065	Fatty acid synthase beta subunit dehydratase
C1G0P4	long-chain acyl-CoA synthetase [EC:6.2.1.3]
C1G106	3-oxoacyl-[acyl-carrier protein] reductase
C1G239	Protein PBN1

C1G2R7	acyl-coenzyme A thioesterase 13 [EC:3.1.2.-]
C1G4Q3	Glycerol 3-phosphatase 1
C1G699	Squalene monooxygenase
C1G6W7	4HBT domain-containing protein
C1G873	Sphingosine-1-phosphate phosphohydrolase
C1G9R2	Diacylglycerol O-acyltransferase
C1G9R7	Cytochrome P450 51
C1G9V7	Patatin-like phospholipase domain-containing protein
C1GBJ3	ATP citrate synthase
C1GC21	sterol carrier protein 2 [EC:2.3.1.176]
C1GCQ2	3,2-trans-enoyl-CoA isomerase, Delta(2)-enoyl-CoA isomerase
C1GCZ4	Long-chain specific acyl-CoA dehydrogenase
C1GD48	ethanolamine-phosphate cytidylyltransferase [EC:2.7.7.14]
C1GDE5	propionyl-CoA synthetase [EC:6.2.1.17]
C1GDH4	leukotriene-A4 hydrolase [EC:3.3.2.6]
C1GDJ1	acetyl-CoA carboxylase / biotin carboxylase 1 [EC:6.4.1.2 6.3.4.14 2.1.3.15]
C1GDN1	Beta-ketoacyl-acyl-carrier-protein synthase II
C1GFX9	Glycolipid transferprotein HET-C2
C1GGA8	Long-chain-fatty-acid-CoA ligase
C1GKG2	17beta-estradiol 17-dehydrogenase / very-long-chain 3-oxoacyl-CoA reductase [EC:1.1.1.62 1.1.1.330]
C1GLB2	C-22 sterol desaturase
**Virulence factors**	
A0A0A0HR13	Alcohol dehydrogenase
C1G3N5	Thioredoxin reductase
C1G450	Alcohol dehydrogenase zinc-binding domain-containing protein
C1G489	superoxide dismutase, Fe-Mn family [EC:1.15.1.1]
C1G4T8	Superoxide dismutase
C1G7E0	Thioredoxin domain-containing protein
C1GAY0	Cell wall glucanase (Utr2)
C1GBB1	Peroxiredoxin 5, atypical 2-Cys peroxiredoxin
C1GBS5	1,3-beta-glucanosyltransferase
C1GDK8	Thioredoxin domain-containing protein
C1GE18	Thioredoxin
C1GE86	Survival factor 1
**Other metabolisms**	
A0A0A0HQV2	NAD(P)H-hydrate epimerase [EC:5.1.99.6]
A0A0A0HRF1	Hydroxyacylglutathione hydrolase
A0A0A0HTY3	1,4-alpha-glucan branching enzyme [EC:2.4.1.18]
A0A0A0HU84	Terpenoid synthase
A0A0A0HUK5	Pyruvate decarboxylase
A0A0A0HUQ2	AB hydrolase-1 domain-containing protein
A0A0A0HV38	Cysteine desulfurase
A0A0A0HV89	Alkaline phosphatase
A0A0A0HWR3	phosphoribosylformylglycinamidine synthase [EC:6.3.5.3]
A0A0A0HXV8	Glycolate oxidase, subunit GlcD

A0A0A0HYM8	Ribose-phosphate diphosphokinase
A0A0A0HYZ8	Guanylate kinase
C1FYK8	cyclin-dependent kinase [EC:2.7.11.22]
C1FYN6	UDP-galactopyranose mutase
C1FYX3	CAP20 protein
C1FYZ9	Saccharopine dehydrogenase
C1FZ38	Phospholipase
C1FZ45	GST N-terminal domain-containing protein
C1FZ74	Uracil phosphoribosyltransferase
C1FZC4	2-nitropropane dioxygenase
C1FZD3	Uridylate kinase
C1FZD8	Trehalose 6-phosphate synthase
C1FZS1	Fe2OG dioxygenase domain-containing protein
C1G019	Guanine deaminase
C1G022	Hydantoinase
C1G072	Inositol-1-monophosphatase
C1G0D2	Xanthine phosphoribosyltransferase 1
C1G0E1	urate oxidase [EC:1.7.3.3]
C1G0H6	NAD dependent epimerase/dehydratase family protein
C1G0Q2	ADP-ribosylation factor GTPase-activating protein 2/3
C1G0Q3	Dihydropteroate synthase
C1G0T1	Glycogenin glucosyltransferase
C1G0V6	Protoglobin domain-containing protein
C1G0X0	Vacuolar membrane protease
C1G0X3	2-succinylbenzoate-CoA ligase
C1G0Y9	Glutaredoxin
C1G161	Peroxisomal dehydratase
C1G164	phosphoacetylglucosamine mutase [EC:5.4.2.3]
C1G168	succinate---hydroxymethylglutarate CoA-transferase [EC:2.8.3.13]
C1G1A3	Mitochondrial methylglutaconyl-CoA hydratase
C1G1F4	Ankyrin repeat protein
C1G1J5	Lactam utilization protein lamB
C1G1W6	Mannose-6-phosphate isomerase
C1G1X9	Alpha-1,4 glucan phosphorylase
C1G208	Geranylgeranyl pyrophosphate synthase
C1G215	Phosphoglycolate phosphatase
C1G217	Nudix hydrolase domain-containing protein
C1G245	Sugar phosphate phosphatase
C1G246	Serine/threonine-protein phosphatase
C1G2D3	Choline-phosphate cytidylyltransferase
C1G2L8	Delta-aminolevulinic acid dehydratase
C1G2Z8	Pyridoxal phosphate homeostasis protein
C1G343	Dihydroorotate dehydrogenase
C1G347	ACB domain-containing protein
C1G356	Mannitol-1-phosphate 5-dehydrogenase
C1G3A8	Inosine-uridine preferring nucleoside hydrolase

C1G3H0	Short chain dehydrogenase/reductase family
C1G3K3	NADPH dehydrogenase
C1G3N0	Citrate lyase subunit beta
C1G3V6	L-tyrosine decarboxylase
C1G3Z9	Kynurenine-oxoglutarate transaminase
C1G479	Mannose-1-phosphate guanyltransferase
C1G4I6	Protein phosphatase
C1G4W6	NADH dehydrogenase
C1G534	Epimerase domain-containing protein
C1G5D0	Glutamate--cysteine ligase
C1G5D2	Striatin Pro11
C1G5H5	Bromo domain-containing protein
C1G5I8	carbamoyl-phosphate synthase large subunit [EC:6.3.5.5]
C1G5J3	ATP-dependent (S)-NAD(P)H-hydrate dehydratase
C1G5L8	Pirin
C1G5P1	NAK protein kinase
C1G5S1	Glutathione S-transferase kappa
C1G5Z2	acetyl-CoA hydrolase [EC:3.1.2.1]
C1G620	4-coumarate-CoA ligase
C1G624	GTPase-activating protein GYP2
C1G653	Nucleoside-diphosphate-sugar epimerase
C1G6D2	Inositol polyphosphate phosphatase
C1G6D9	Secondary thiamine-phosphate synthase enzyme
C1G6V8	Adenylyl-sulfate kinase
C1G713	H+-translocating NAD(P) transhydrogenase [EC:1.6.1.2 7.1.1.1]
C1G7E4	Aldehyde dehydrogenase
C1G7R6	metallopeptidase MepB [EC:3.4.24.-]
C1G7T9	5'-deoxynucleotidase
C1G7X1	S-(hydroxymethyl)glutathione dehydrogenase
C1G855	Acetyl-coenzyme A transporter 1
C1G898	Aldehyde dehydrogenase
C1G8E0	M protein repeat protein
C1G8F8	Ser/Thr protein phosphatase
C1G8J4	Beta-lactamase domain-containing protein
C1G8V5	Glycosidase
C1G8V9	Beta-hexosaminidase
C1G8X2	Thiamine thiazole synthase
C1G9D5	urease [EC:3.5.1.5]
C1G9D6	cytochrome-b5 reductase [EC:1.6.2.2]
C1G9K7	phosphomannomutase [EC:5.4.2.8]
C1G9L3	Orotate phosphoribosyltransferase
C1G9M7	30 kDa heat shock protein
C1G9M8	Fumarylacetoacetate hydrolase domain-containing protein
C1G9U6	Uroporphyrinogen decarboxylase
C1GA02	Zinc metalloprotease
C1GA81	Aspartyl aminopeptidase

C1GAA3	Morphogenesis protein
C1GAA7	Cystathionine beta-synthase
C1GAC7	Tyrosinase
C1GAC9	Altered inheritance of mitochondria protein 24, mitochondrial
C1GAG4	C-1-tetrahydrofolate synthase
C1GAH9	D-xylulose reductase A
C1GAK7	Adenine phosphoribosyltransferase
C1GAM6	C-1-tetrahydrofolate synthase
C1GAN6	Rhodocoxin reductase
C1GAT1	Amidase
C1GAT8	UTP--glucose-1-phosphate uridylyltransferase [EC:2.7.7.9]
C1GAT9	Oxysterol-binding protein
C1GAV1	Nicotinamidase
C1GAY4	peptide-methionine (S)-S-oxide reductase [EC:1.8.4.11]
C1GB24	Serine/threonine-protein kinase srk1
C1GB27	adenylosuccinate lyase [EC:4.3.2.2]
C1GB32	3,4-dihydroxy-2-butanone-4-phosphate synthase
C1GB51	Epsin
C1GB78	uroporphyrinogen-III synthase [EC:4.2.1.75]
C1GB99	Trehalose-6-phosphate synthase
C1GBF1	AFG3 family protein [EC:3.4.24.-]
C1GBQ7	chorismate mutase [EC:5.4.99.5]
C1GBS0	carbamoyl-phosphate synthase / aspartate carbamoyltransferase [EC:6.3.5.5 2.1.3.2]
C1GBT1	Serine/threonine-protein phosphatase
C1GBW5	Chorismate synthase
C1GC65	Glutathione peroxidase
C1GCB4	Dienelactone hydrolase family protein
C1GCG5	Glucose 1-dehydrogenase
C1GCJ9	Amidophosphoribosyltransferase
C1GCP5	Cleft lip and palate transmembrane protein
C1GCX5	glycine hydroxymethyltransferase [EC:2.1.2.1]
C1GD47	Monothiol glutaredoxin-5
C1GD57	Xaa-Pro dipeptidase [EC:3.4.13.9]
C1GD89	Orotidine 5'-phosphate decarboxylase
C1GD97	Malic enzyme
C1GDM0	Isochorismatase domain-containing protein
C1GDQ6	Ubiquitin carboxyl-terminal hydrolase
C1GDQ9	Alkaline phosphatase
C1GDU7	Pyridoxal 5'-phosphate synthase
C1GDX6	Pyridox_oxase_2 domain-containing protein
C1GDY6	GMP synthase
C1GDY8	Nudix hydrolase domain-containing protein
C1GE04	Molybdopterin binding domain-containing protein
C1GE06	Oxidoreductase, variant 2
C1GE37	NADPH2:quinone reductase
C1GE78	Porphobilinogen deaminase

C1GED3	Glycerate-and formate-dehydrogenase
C1GEH1	Pyruvate decarboxylase
C1GEU2	Glycogen [starch] synthase
C1GEU7	Farnesyl pyrophosphate synthetase
C1GEX6	CTP synthase
C1GEY4	Xaa-Pro aminopeptidase [EC:3.4.11.9]
C1GEY6	pyridoxal 5'-phosphate synthase pdxS subunit [EC:4.3.3.6]
C1GF19	Lactonohydrolase
C1GFB1	Nicotinate-nucleotide pyrophosphorylase [carboxylating]
C1GFB5	Aldose 1-epimerase
C1GFG3	Phosphatidylinositol-4-phosphate 5-kinase its3
C1GFR5	ABM domain-containing protein
C1GFS6	Phosphoribosylaminoimidazole-succinocarboxamide synthase
C1GFV7	BAR adaptor protein RVS167
C1GFX8	manganese-transporting P-type ATPase [EC:7.2.2.-]
C1GG52	Lysine decarboxylase-like protein
C1GG60	Phosphoribosylamine-glycine ligase
C1GG92	Tetratricopeptide repeat domain-containing protein
C1GGC2	Cysteine proteinase 1, mitochondrial
C1GGQ3	Formamidase
C1GGQ6	Phosphoglucomutase
C1GGV9	Aminopeptidase
C1GGY0	Phenol 2-monooxygenase
C1GGY3	Arp2/3 complex subunit Arc16
C1GGY5	Salicylate hydroxylase
C1GGY6	Acid phosphatase
C1GH49	GMP synthase [glutamine-hydrolyzing]
C1GH68	Acid phosphatase
C1GHG2	Amidase
C1GHK4	Betaine aldehyde dehydrogenase
C1GHS5	Amidase
C1GHU1	D-arabinono-1,4-lactone oxidase
C1GHW9	Dihydroxyacetone kinase
C1GI49	Phosphatidylinositol 4-kinase
C1GI75	Universal stress protein
C1GIC4	Urease accessory protein UreG
C1GIE5	Hydroxymethylglutaryl-CoA lyase
C1GIN4	Annexin
C1GIR6	Lectin, mannose-binding 2
C1GJ33	Phenylacetate 2-hydroxylase
C1GJ51	WD repeat-containing protein
C1GJJ9	Sorbitol utilization protein SOU2
C1GJU2	Methylenetetrahydrofolate reductase
C1GJU4	thymidylate kinase
C1GJZ9	inosine-5'-monophosphate dehydrogenase
C1GK29	Glucan 1,3-beta-glucosidase

C1GKB3	S-formylglutathione hydrolase
C1GKJ4	Phosphoribosylglycinamide formyltransferase
C1GKJ6	Deoxyuridine 5'-triphosphate nucleotidohydrolase
C1GKJ7	APH domain-containing protein
C1GKN3	Endo-1,3(4)-beta-glucanase
C1GKQ6	Chitin synthase
C1GKW3	Dihydroorotase
C1GL27	Esterase/lipase
C1GL55	Oligosaccharyltransferase
C1GLD0	Purine nucleoside phosphorylase
C1GLE2	D-xylose reductase
C1GLG2	adenylate kinase [EC:2.7.4.3]
C1GLG4	NADP:D-xylose dehydrogenase
C1GLH2	Coproporphyrinogen oxidase
C1GLJ8	CBS and PB1 domain-containing protein
C1GLK9	PPM-type phosphatase domain-containing protein
C1GLP9	Diphosphomevalonate decarboxylase
C1GLZ5	Glycos_transf_1 domain-containing protein
C1GMG6	3'(2'),5'-bisphosphate nucleotidase
C1GMI8	Mannose-1-phosphate guanyltransferase
C1GMP4	Thymidylate synthase
C1GN16	Hydroxyacid-oxoacid transhydrogenase
**Cytoskeleton**	
A0A0A0HS41	Spindle pole body component
C1FYR9	Actin-related protein 2/3 complex subunit 5
C1FYZ5	PTK9 protein tyrosine kinase 9
C1FZC6	Actin cytoskeleton-regulatory complex protein PAN1
C1FZZ3	Profilin
C1G0N2	Actin cytoskeleton protein (VIP1)
C1G1V9	Actin-related protein 2
C1G5M0	Actin cytoskeleton-regulatory complex protein SLA1
C1G9F9	dynein light chain, cytoplasmic
C1G9M3	Actin-related protein 2/3 complex subunit
C1GDK5	Fimbrin
C1GE05	ADF-H domain-containing protein
C1GFT4	Cytoskeleton assembly control protein
C1GJ13	coronin-1B/1C/6
C1GKE7	Arp2/3 complex 34 kDa subunit
C1GLX7	Cofilin
C1GM22;C1G3F2	Tubulin alpha chain
C1GN88	RPEL repeat protein
**Signal transduction**	
C1FYL8	Rab-GAP TBC domain-containing protein
C1FYR1	Rab family, other
C1FZF8	Rho-GTPase-activating protein
C1G0A1	GDP/GTP exchange factor

C1G0G7	Non-specific serine/threonine protein kinase
C1G6I9	Chimerin
C1G749	Target of rapamycin complex subunit LST8
C1GB04	14-3-3 protein epsilon
C1GB79	Mitogen-activated protein kinase
C1GDI1	Guanyl-nucleotide exchange factor
C1GED5	Guanine nucleotide-binding protein subunit beta
C1GFR7	GTPase activating protein
C1GG23	AMPKBI domain-containing protein
C1GGW3	Glucose repression regulatory protein TUP1
C1GJJ1	Serine/threonine-protein phosphatase
C1GK66	Calcium/calmodulin-dependent protein kinase
C1GKC8	Ras GTPase-activating protein-binding protein 2
C1GMA5	cAMP-dependent protein kinase regulatory subunit
**No annotation found**	
A0A0A0HRV0	Integral membrane protein
A0A0A0HSY2	
A0A0A0HT22	AMPK1_CBM domain-containing protein
A0A0A0HTY7	
C1FYH3	NA
C1FYI0	
C1FYP5	
C1FZ07	PHD-type domain-containing protein
C1FZH0	F-box domain-containing protein
C1FZM0	MGS207 protein
C1FZP5	C2H2-type domain-containing protein
C1FZV0	
C1G031	Short-chain dehydrogenase
C1G060	C2H2-type domain-containing protein
C1G080	
C1G0G5	
C1G0J8	
C1G0N1	
C1G0S3	
C1G101	NA
C1G1D0	TAM domain methyltransferase
C1G1F0	
C1G1F8	
C1G1H9	Lgl_C domain-containing protein
C1G1L3	Hsp70-like protein
C1G1R0	
C1G1S6	
C1G1U8	
C1G267	Vacuolar membrane protein pep3
C1G2F3	
C1G2U9	NA

C1G2W9	
C1G317	
C1G3J7	
C1G3W9	GTPase activating protein
C1G4K0	
C1G4N7	
C1G4Y0	
C1G521	
C1G558	
C1G5B6	
C1G5G9	
C1G5K7	
C1G5S4	
C1G5V2	
C1G5X0	Carrier domain-containing protein
C1G606	Parasitic phase-specific protein PSP-1
C1G614	
C1G647	NA
C1G659	SAP domain-containing protein
C1G673	
C1G6B9	RNA binding protein
C1G6M1	WH2 domain-containing protein
C1G6P0	CORD and CS domain-containing protein
C1G6V4	
C1G726	ThiJ/PfpI family protein
C1G776	cobW domain-containing protein
C1G7A9	Rhodanese domain-containing protein
C1G7T7	CobW domain-containing protein
C1G825	
C1G8E3	BSD domain-containing protein
C1G8P2	
C1G8R8	
C1G8U8	
C1G8V1	
C1G8W5	RED_N domain-containing protein
C1G991	
C1G9F5	FCP1 homology domain-containing protein
C1GA88	
C1GAH4	
C1GAQ7	
C1GAY7	NA
C1GB06	
C1GBH1	
C1GBH2	
C1GCD4	
C1GD13	

C1GD64	
C1GD94	
C1GDR5	
C1GDW6	
C1GDZ9	
C1GE44	
C1GEL5	
C1GEL7	
C1GES0	apoptosis-inducing factor 2
C1GFA1	
C1GFT1	NA
C1GG25	
C1GG74	
C1GGJ5	
C1GGL6	
C1GH51	
C1GH86	
C1GHF4	
C1GHL9	NA
C1GHP7;C1G8U0	
C1GIH9	
C1GII7	
C1GII8	
C1GIP9	NA
C1GIR3	
C1GJ28	Stress-response A/B barrel domain-containing protein
C1GJ98	
C1GJH6	NA
C1GJH8	NA
C1GJJ0	
C1GJP9	
C1GJT4	
C1GK89	
C1GKF7	
C1GKR0	
C1GKX2	
C1GL73	
C1GLF5	
C1GLX2	WW domain-containing protein
C1GM11	
C1GM66	
C1GM71	
C1GMC8	Rick_17kDa_Anti domain-containing protein
C1GMJ4	RING-type domain-containing protein
C1GMN0	AmmeMemoRadiSam system protein B
C1GMS2	

C1GN52	GPR/FUN34 family protein
C1GN66	
