# Supplementary material for: Transcriptional profiling of a fungal granuloma reveals a low metabolic activity of Paracoccidioides brasiliensis yeasts and an actively regulated host immune response
Source: Front Cell Infect Microbiol. 2023 Oct 5;13:1268959. doi: 10.3389/fcimb.2023.1268959 (PMC10585178; doi:10.3389/fcimb.2023.1268959)
Supplement: Supplementary file 12 [file Table_11.pdf]

Supplementary Table 11. Upregulated proteins of the isolated yeasts

Acession number	Protein	Expression status	Log (Fold Change)	Adjusted p-value
**Energy metabolism**				
C1FYW8	chitinase [EC:3.2.1.14]	UP (12W)	2,287651	0,01163489
C1G786	phosphatidylinositol 4-phosphatase [EC:3.1.3.-]	UP (12W)	2,073699	0,01163489
C1G736	acyl-CoA oxidase [EC:1.3.3.6]	UP(D)	1,997825	0,000799021
C1GM15	O-acetylhomoserine/O-acetylserine sulfhydrylase [EC:2.5.1.49 2.5.1.47]	UP (12W)	1,849912	0,01816612
C1G4C1	1-phosphatidylinositol phosphodiesterase [EC:4.6.1.13]	UP(D)	1,73708	0,000889849
C1FZV1	ribulose-phosphate 3-epimerase [EC:5.1.3.1]	UP (8W)	1,712969	0,005373348
C1G893	Acyl-coenzyme A thioesterase THEM4	UP (12W)	1,671263	0,025542115
C1GKK3	F-type H+-transporting ATPase subunit delta	UP(D)	1,500595	0,00012915
C1GCH2	Cytochrome b mRNA-processing protein 4	UP (8W)	1,451101	0,016683432
C1G5J9	NADH-ubiquinone oxidoreductase	UP(D)	1,386248	2,4505E-05
C1GCI0	malate synthase [EC:2.3.3.9]	UP (8W)	1,281083	0,006588026
C1GLZ6	citrate synthase [EC:2.3.3.1]	UP(D)	1,256515	6,79495E-05
C1G137	electron-transferring-flavoprotein dehydrogenase [EC:1.5.5.1]	UP(D)	1,245486	0,000389073
C1G440	fructose-1,6-bisphosphatase I [EC:3.1.3.11]	UP (8W)	1,228492	0,003306043
C1G8L6	Cytochrome b5 heme-binding domain-containing protein	UP (8W)	1,180241	0,034203032
C1GLI3	fumarate hydratase, class II [EC:4.2.1.2]	UP (8W)	1,16717	0,003306043
C1GKU3	hexokinase [EC:2.7.1.1]	UP(D)	1,166301	9,52822E-05
C1G0Z9	Cytochrome b5 heme-binding domain-containing protein	UP (8W)	1,156461	0,026541001
C1G3G7	isocitrate lyase [EC:4.1.3.1]	UP (8W)	1,131485	0,020515279
C1GHR9	Acyl-CoA dehydrogenase	UP (8W)	1,109947	0,00687719
A0A0A0HX82	phosphoglucomutase [EC:5.4.2.2]	UP (8W)	1,104917	0,035563884
C1G056	pyruvate dehydrogenase E1 component beta subunit [EC:1.2.4.1]	UP(D)	1,102255	4,81286E-05
C1G890	sulfite reductase (NADPH) hemoprotein beta-component [EC:1.8.1.2]	UP(D)	1,094721	0,000104273
C1GEA0	V-type H+-transporting ATPase subunit D	UP (8W)	1,089669	0,030675825
C1G6E6	acetyl-CoA C-acetyltransferase [EC:2.3.1.9]	UP (12W)	1,075482	0,042917819
C1GCI7	isocitrate lyase [EC:4.1.3.1]	UP(D)	1,073775	3,01463E-05
C1G9X3	2-phosphoglycerate dehydratase	UP(D)	1,073383	3,96715E-05
C1G6C3	sulfite oxidase [EC:1.8.3.1]	UP (12W)	1,06423	0,022208889

C1GLV8	F-type H+-transporting ATPase subunit beta [EC:7.1.2.2]	UP(D)	1,054902	1,34334E-05
C1G6K0	isocitrate dehydrogenase (NAD+) [EC:1.1.1.41]	UP(D)	1,038524	8,32334E-05
C1GA89	inorganic pyrophosphatase [EC:3.6.1.1]	UP(D)	1,036182	7,68103E-05
A0A0A0HRM2	V-type H+-transporting ATPase subunit A [EC:7.1.2.2]	UP(D)	1,032296	0,002071441
C1G294	succinyl-CoA synthetase alpha subunit [EC:6.2.1.4 6.2.1.5]	UP(D)	1,023307	2,4505E-05
C1GCX3	2,3-bisphosphoglycerate-independent phosphoglycerate mutase [EC:5.4.2.12]	UP(D)	1,020784	8,26625E-05
C1G278	acyl-CoA dehydrogenase [EC:1.3.8.7]	UP (8W)	1,013341	0,021884365
C1G547	pyruvate dehydrogenase E1 component alpha subunit [EC:1.2.4.1]	UP (8W)	1,00685	0,020389391
A0A0A0HUI2	NADH dehydrogenase [ubiquinone] 1 alpha subcomplex assembly factor 3	UP (12W)	1,006754	0,026397315
C1GGE5	acetyl-CoA C-acetyltransferase [EC:2.3.1.9]	UP (8W)	1,006458	0,00766075
C1GKQ0	Pyruvate decarboxylase	UP (8W)	0,998956	0,018887049
C1GIX7	pyruvate dehydrogenase E2 component (dihydrolipoamide acetyltransferase) [EC:2.3.1.12]	UP(D)	0,992972	3,04379E-05
C1G1H4	pyruvate decarboxylase [EC:4.1.1.1]	UP (8W)	0,981041	0,031728351
A0A0A0HST0	F-type H+-transporting ATPase subunit g	UP (8W)	0,975137	0,016824995
C1G002	6-phosphofructokinase 1 [EC:2.7.1.11]	UP(D)	0,974763	0,000203639
C1FZY1	L-lactate dehydrogenase (cytochrome) [EC:1.1.2.3]	UP(D)	0,969166	0,000414153
C1GKI5	6-phosphogluconolactonase [EC:3.1.1.31]	UP (12W)	0,957917	0,0402877
C1G1C8	fructose-bisphosphate aldolase, class II [EC:4.1.2.13]	UP (8W)	0,955297	0,018970081
C1GI20	triosephosphate isomerase (TIM) [EC:5.3.1.1]	UP(D)	0,953067	3,69337E-05
C1G421	acetyl-CoA acyltransferase 1 [EC:2.3.1.16]	UP(D)	0,948955	0,000242791
C1G7N9	acetyl-CoA acyltransferase 1 [EC:2.3.1.16]	UP(D)	0,948085	0,000299791
C1GBX3	oxalate---CoA ligase [EC:6.2.1.8]	UP(D)	0,945242	0,000224532
C1G0C7	succinyl-CoA synthetase beta subunit [EC:6.2.1.4 6.2.1.5]	UP(D)	0,933972	0,00015078
C1GAG3	isocitrate dehydrogenase [EC:1.1.1.42]	UP(D)	0,931739	0,000284775
C1GCI8	citrate synthase [EC:2.3.3.1]	UP(D)	0,926697	0,000379134
C1GJI4	transaldolase [EC:2.2.1.2]	UP(D)	0,91853	0,000210172
C1GIX4	malate dehydrogenase [EC:1.1.1.37]	UP (8W)	0,918035	0,00687719

C1G5V6	F-type H+-transporting ATPase subunit alpha	UP(D)	0,907412	0,000157398
C1GMZ1	multifunctional beta-oxidation protein [EC:4.2.1.- 1.1.1.-]	UP(D)	0,893796	8,21503E-05
C1G8R5	6-phosphogluconate dehydrogenase [EC:1.1.1.44 1.1.1.343]	UP (12W)	0,89173	0,034781743
C1GC82	transketolase [EC:2.2.1.1]	UP(D)	0,887039	0,000155336
C1G2P3	enoyl-CoA hydratase [EC:4.2.1.17]	UP(D)	0,886518	0,000267669
C1G8H3	cytochrome b5	UP (8W)	0,869989	0,042340628
C1G7K8	cytochrome c peroxidase [EC:1.11.1.5]	UP(D)	0,860697	0,000634175
A0A0A0HUM 5	aconitate hydratase [EC:4.2.1.3]	UP(D)	0,859641	0,000267669
C1G5Y7	Cytochrome b5 heme-binding domain-containing protein	UP(D)	0,85448	0,000299791
C1GMD0	NADH dehydrogenase (ubiquinone) Fe-S protein 8 [EC:7.1.1.2]	UP (12W)	0,852547	0,049199686
C1GK20	D-xylose reductase	UP (8W)	0,834838	0,010164498
C1G2W2	pyruvate kinase [EC:2.7.1.40]	UP(D)	0,831881	7,68103E-05
C1GBZ4	glutamate dehydrogenase (NADP+) [EC:1.4.1.4]	UP (12W)	0,824982	0,03059419
C1GLX6	cytochrome c oxidase subunit 4	UP (12W)	0,820956	0,040504859
C1GG42	NADH dehydrogenase (ubiquinone) 1 alpha subcomplex subunit 6	UP(D)	0,81728	0,000904473
A0A0A0HY53	NADPH-dependent diflavin oxidoreductase 1	UP(D)	0,781448	0,000783867
C1G9P1	isocitrate dehydrogenase (NAD+) [EC:1.1.1.41]	UP (8W)	0,780621	0,028162637
C1GLB8	malate dehydrogenase [EC:1.1.1.37]	UP (8W)	0,733022	0,028710327
C1G256	NADH dehydrogenase [ubiquinone] 1 alpha subcomplex subunit	UP (8W)	0,685861	0,033558288
C1G5F6	glyceraldehyde 3-phosphate dehydrogenase (phosphorylating) [EC:1.2.1.12]	UP (8W)	0,64594	0,040733231
**Virulence factors**				
C1GLU3	Alcohol dehydrogenase	UP (8W)	1,97199	0,003306043
C1G2U7	superoxide dismutase, Fe-Mn family [EC:1.15.1.1]	UP(D)	1,400316	3,96715E-05
C1G7L4	alpha-1,3-glucan synthase [EC:2.4.1.183]	UP(D)	1,217064	0,000224532
C1GDG7	Beta-1,6-glucan boisynthesis protein	UP (8W)	1,169829	0,015915364
C1G489	superoxide dismutase, Fe-Mn family [EC:1.15.1.1]	UP (8W)	1,167965	0,005443781
**Amino acid metabolism**				
C1GMI2	4-hydroxyphenylpyruvate dioxygenase [EC:1.13.11.27]	UP (8W)	1,88694	0,003306043
C1G7F9	3-deoxy-7-phosphoheptulonate synthase [EC:2.5.1.54]	UP(D)	1,830968	9,02668E-05
C1G949	ATP phosphoribosyltransferase	UP (8W)	1,700403	0,019833298
C1G792	threonine dehydratase [EC:4.3.1.19]	UP (8W)	1,622606	0,003306043

C1G0F9	kynureninase [EC:3.7.1.3]	UP (8W)	1,610815	0,005443781
C1G4U7	Glycine cleavage system H protein	UP (8W)	1,494766	0,047711396
C1G8V0	aspartate aminotransferase, cytoplasmic [EC:2.6.1.1]	UP (8W)	1,476232	0,007296661
C1GB53	anthranilate synthase component I [EC:4.1.3.27]	UP(D)	1,460526	3,82641E-05
C1GD55	5-oxoprolinase (ATP-hydrolysing) [EC:3.5.2.9]	UP(D)	1,419538	0,000117426
C1GHZ0	3-hydroxyisobutyryl-CoA hydrolase [EC:3.1.2.4]	UP (8W)	1,352655	0,00766075
C1GHM0	Histidine biosynthesis trifunctional protein	UP (8W)	1,329082	0,025112452
C1GJ05	dihydroxy-acid dehydratase [EC:4.2.1.9]	UP(D)	1,268743	0,000108069
C1G4R2	S-adenosylmethionine synthetase [EC:2.5.1.6]	UP(D)	1,266777	0,000168286
C1GLT7	5-methyltetrahydropteroyltriglutamate--homocysteine methyltransferase [EC:2.1.1.14]	UP(D)	1,200347	5,40638E-05
C1GJD0	3-methylcrotonyl-CoA carboxylase alpha subunit [EC:6.4.1.4]	UP(D)	1,180525	3,69337E-05
C1G3P8	malonate-semialdehyde dehydrogenase (acetylating) / methylmalonate-semialdehyde dehydrogenase [EC:1.2.1.18 1.2.1.27]	UP(D)	1,176664	1,17191E-05
C1GAY3	proline dehydrogenase [EC:1.5.5.2]	UP (8W)	1,156817	0,005276659
C1G2X0	homoisocitrate dehydrogenase [EC:1.1.1.87]	UP(D)	1,153866	0,000141366
C1GGQ7	dihydrolipoamide dehydrogenase [EC:1.8.1.4]	UP(D)	1,142627	3,96715E-05
A0A0A0HTR8	omega-amidase [EC:3.5.1.3]	UP (8W)	1,116326	0,003306043
C1G3G3	gamma-glutamyltranspeptidase / glutathione hydrolase [EC:2.3.2.2 3.4.19.13]	UP (8W)	1,1077	0,017093056
A0A0A0HRQ4	glutaryl-CoA dehydrogenase [EC:1.3.8.6]	UP(D)	1,09719	0,000242791
C1G6V9	aminomethyltransferase [EC:2.1.2.10]	UP(D)	1,090372	0,000514068
C1G8P1	2-oxoisovalerate dehydrogenase E1 component beta subunit [EC:1.2.4.4]	UP(D)	1,088197	0,002723494
C1GLM1	asparagine synthase (glutamine-hydrolysing) [EC:6.3.5.4]	UP(D)	1,087294	0,000815211
C1G3V5	aspartate aminotransferase, mitochondrial [EC:2.6.1.1]	UP (8W)	1,080226	0,021163629
C1GIE3	acetylornithine aminotransferase [EC:2.6.1.11]	UP(D)	1,078031	0,000352749
C1G9P8	glutamine---fructose-6-phosphate transaminase (isomerizing) [EC:2.6.1.16]	UP (12W)	1,03215	0,018150971

C1GBD8	3-oxoacid CoA-transferase [EC:2.8.3.5]	UP(D)	1,030689	0,000251289
C1G020	glycine dehydrogenase [EC:1.4.4.2]	UP(D)	1,025612	0,000361455
C1G8C8	2-oxoisovalerate dehydrogenase E1 component alpha subunit [EC:1.2.4.4]	UP (12W)	1,022733	0,035275544
C1FYJ9	pentafunctional AROM polypeptide [EC:4.2.3.4 4.2.1.10 1.1.1.25 2.7.1.71 2.5.1.19]	UP(D)	1,014849	0,000246651
C1GJD4	3-methylcrotonyl-CoA carboxylase beta subunit [EC:6.4.1.4]	UP (8W)	1,005172	0,029479502
C1GF86	Cys-Gly metallodipeptidase DUG1 [EC:3.4.13.-]	UP(D)	0,990064	0,000299791
C1FZL3	dipeptidyl aminopeptidase B [EC:3.4.14.-]	UP(D)	0,981364	7,68103E-05
A0A0A0HX63	acetolactate synthase I/II/III large subunit [EC:2.2.1.6]	UP (12W)	0,980707	0,021736563
C1G312	ornithine--oxo-acid transaminase [EC:2.6.1.13]	UP(D)	0,971222	0,001750463
C1G596	3-hydroxyisobutyrate/3-hydroxypropionate dehydrogenase [EC:1.1.1.31 1.1.1.59]	UP (12W)	0,95798	0,023164795
C1G7L2	3-isopropylmalate dehydratase [EC:4.2.1.33]	UP(D)	0,957441	0,000334937
C1GJ38	anthranilate synthase / indole-3-glycerol phosphate synthase / phosphoribosylanthranilate isomerase [EC:4.1.3.27 4.1.1.48 5.3.1.24]	UP (8W)	0,954045	0,032849372
C1G452	saccharopine dehydrogenase (NADP+, L-glutamate forming) [EC:1.5.1.10]	UP(D)	0,936795	0,000490826
C1GI69	tryptophan synthase [EC:4.2.1.20]	UP(D)	0,929358	0,000157398
C1G6H2	threonine synthase [EC:4.2.3.1]	UP (8W)	0,928831	0,017024344
C1GDG5	cystathionine beta-synthase [EC:4.2.1.22]	UP(D)	0,921903	3,96715E-05
C1GLF6	L-2-aminoadipate reductase [EC:1.2.1.95]	UP (8W)	0,918797	0,01136855
C1GJD3	isovaleryl-CoA dehydrogenase [EC:1.3.8.4]	UP(D)	0,907459	4,72181E-05
C1GCG7	Arginine-requiring protein 2	UP (8W)	0,903039	0,043289385
C1GBT4	1-pyrroline-5-carboxylate dehydrogenase [EC:1.2.1.88]	UP(D)	0,899338	0,000192931
C1G1E5	hydroxymethylglutaryl-CoA synthase [EC:2.3.3.10]	UP(D)	0,863688	0,000330967
C1G7A3	succinate-semialdehyde dehydrogenase / glutarate-semialdehyde dehydrogenase [EC:1.2.1.16 1.2.1.79 1.2.1.20]	UP(D)	0,80927	0,000205583
C1G0L5	Choline dehydrogenase	UP(D)	0,807895	0,000190119

C1FYL2	argininosuccinate synthase [EC:6.3.4.5]	UP (8W)	0,738199	0,020389391
**Lipid metabolism**				
C1G239	Protein PBN1	UP (12W)	2,94041	0,021905016
C1GM85	phospholipid:diacylglycerol acyltransferase [EC:2.3.1.158]	UP(D)	2,142193	2,03704E-05
C1G6N9	ethanolamine kinase [EC:2.7.1.82]	UP (8W)	2,088055	0,026728024
C1GDJ1	acetyl-CoA carboxylase / biotin carboxylase 1 [EC:6.4.1.2 6.3.4.14 2.1.3.15]	UP(D)	1,642269	8,26625E-05
C1GMV6	Squalene synthase	UP(D)	1,60452	0,000345886
C1G064	fatty acid synthase subunit alpha, fungi type [EC:2.3.1.86]	UP (8W)	1,60077	0,016683432
C1GKG2	17beta-estradiol 17-dehydrogenase / very-long-chain 3-oxoacyl-CoA reductase [EC:1.1.1.62 1.1.1.330]	UP(D)	1,478377	0,000783867
C1FYG6	Phosphatidate cytidylyltransferase, mitochondrial	UP (12W)	1,415348	0,049944763
C1GDH4	leukotriene-A4 hydrolase [EC:3.3.2.6]	UP(D)	1,334074	0,00076067
C1G054	mitochondrial enoyl-[acyl-carrier protein] reductase / trans-2-enoyl-CoA reductase [EC:1.3.1.- 1.3.1.38]	UP(D)	1,283947	0,000118
C1GBJ2	ATP citrate synthase	UP(D)	1,230404	1,20156E-05
C1GBJ3	ATP citrate synthase	UP(D)	1,202373	3,04379E-05
C1G2R7	acyl-coenzyme A thioesterase 13 [EC:3.1.2.-]	UP (8W)	1,111151	0,014300697
C1GGY2	3-oxoacyl-(acyl-carrier-protein) reductase	UP (8W)	1,106916	0,004704236
C1GDE5	propionyl-CoA synthetase [EC:6.2.1.17]	UP(D)	1,097055	0,000330967
C1GD48	ethanolamine-phosphate cytidylyltransferase [EC:2.7.7.14]	UP (8W)	1,024057	0,034729177
C1G0P4	long-chain acyl-CoA synthetase [EC:6.2.1.3]	UP (12W)	0,976811	0,017605147
C1GC21	sterol carrier protein 2 [EC:2.3.1.176]	UP (12W)	0,849111	0,046563052
C1GMW7	sphingolipid C9-methyltransferase [EC:2.1.1.317]	UP (12W)	0,774656	0,038118607
**Gene/protein regulation**				
C1G5S5	small nuclear ribonucleoprotein G	UP(D)	3,440148	7,40048E-06
C1GML3	mRNA (guanine-N7-)-methyltransferase [EC:2.1.1.56]	UP (8W)	2,892278	0,004911557
C1GIZ3	elongation factor 3	UP(D)	2,62974	2,4505E-05
C1GMY8	Leucine aminopeptidase 1	UP(D)	2,609778	0,001398865
C1G3W8	geranylgeranyl transferase type-2 subunit alpha [EC:2.5.1.60]	UP(D)	2,256317	0,001164899
C1G283	large subunit ribosomal protein L8e	UP(D)	2,208918	1,17191E-05
C1GBM4	large subunit ribosomal protein L26e	UP (8W)	2,15011	0,016346151
C1GA83	ATP-dependent Clp protease, protease subunit [EC:3.4.21.92]	UP(D)	2,13273	0,000127385

C1GGN1	signal recognition particle subunit SRP14	UP (8W)	2,119582	0,02857375
C1FYI4	DNA-directed RNA polymerase subunit	UP (8W)	2,111115	0,014400463
A0A0A0HTF2	tRNA ligase [EC:6.5.1.3]	UP(D)	2,094767	0,000271085
C1GC24	nucleolar protein 15	UP (8W)	2,060639	0,005373348
C1G4B4	Zn(2)-C6 fungal-type domain-containing protein	UP (8W)	2,048338	0,027199995
C1G9F6	protein phosphatase 1 regulatory subunit 7	UP(D)	2,043921	8,21503E-05
C1G3W6	U3 small nucleolar RNA-associated protein 5	UP (8W)	2,039218	0,026277361
A0A0A0HVY3	cullin 1	UP (12W)	2,006905	0,021736563
C1G8D9	serine/threonine-protein phosphatase 2A activator	UP (8W)	2,004832	0,010388376
C1G942	large subunit ribosomal protein L10e	UP(D)	1,924594	1,34334E-05
C1GMR0	small subunit ribosomal protein S29	UP(D)	1,908814	0,000203639
C1GLP2	SAP domain-containing protein	UP (12W)	1,903358	0,026993774
A0A0A0HV62	Ribosomal protein L19	UP(D)	1,882064	6,51881E-05
C1GB13	large subunit ribosomal protein L23e	UP(D)	1,855043	2,03704E-05
A0A0A0HUZ4	26S protease subunit RPT4	UP (8W)	1,827766	0,013621638
C1FZ26	3-demethylubiquinone-9 3-methyltransferase	UP (12W)	1,807556	0,034503547
C1GEM5	5'-3' exoribonuclease 1 [EC:3.1.13.-]	UP (8W)	1,799992	0,043289385
C1GCT4	SBDS domain-containing protein	UP (8W)	1,799138	0,021206174
C1GLD3	FACT complex subunit SPT16	UP(D)	1,778408	7,40048E-06
C1G5E9	Peptidylprolyl isomerase	UP (8W)	1,758151	0,020249991
C1GJ57	RRM domain-containing protein	UP(D)	1,741767	0,000889849
C1GAL5	Prp8 binding protein	UP (8W)	1,734938	0,030608798
C1G9X9	large subunit ribosomal protein L36e	UP(D)	1,710834	0,000330967
C1GE49	small subunit ribosomal protein S5	UP (8W)	1,703213	0,005516536
C1FYJ7	histone H3	UP (12W)	1,68621	0,021989098
C1FYQ6	protein TIF31	UP(D)	1,684905	8,85577E-05
A0A0A0HX33	actin-related protein 8	UP (12W)	1,664309	0,041170587
C1G7Q9	small nuclear ribonucleoprotein E	UP (8W)	1,66351	0,013118378
C1GCS4	splicing factor 3B subunit 3	UP (8W)	1,629771	0,018970081
C1GF85	60S ribosomal protein L20	UP (8W)	1,616	0,017179205
C1G7T1	zinc-finger protein CreA/MIG	UP (8W)	1,585041	0,014402336
C1G9C0	large subunit ribosomal protein L15e	UP(D)	1,575588	1,17191E-05
C1GFX4	translation initiation factor 2 subunit 1	UP(D)	1,57216	0,000108069
C1GK69	DNA-directed RNA polymerases I and III subunit RPAC1	UP (8W)	1,570786	0,026515476
C1G650	elongation factor G	UP (12W)	1,565953	0,049199686
C1GHJ0	large subunit ribosomal protein L17e	UP(D)	1,5587	5,14954E-05
C1GEE2	protein disulfide-isomerase A6 [EC:5.3.4.1]	UP (8W)	1,545698	0,012912291
A0A0A0HUJ5	large subunit ribosomal protein L3e	UP(D)	1,539754	3,01463E-05

C1G9I8	PRA1 family protein 1	UP (8W)	1,516922	0,01155815
C1GJJ6	chloride channel, nucleotide-sensitive, 1A	UP (8W)	1,505049	0,034776299
C1G8T3	mitochondrial protein import protein ZIM17	UP (8W)	1,500885	0,034875515
C1G172	large subunit ribosomal protein L27e	UP(D)	1,474931	3,96715E-05
C1GKL8	protein transport protein SEC23	UP(D)	1,46937	3,04379E-05
C1G540	large subunit ribosomal protein L7/L12	UP (8W)	1,466679	0,037383913
C1G654	THO complex subunit 2	UP (8W)	1,462957	0,043943784
C1FZ57	large subunit ribosomal protein L32e	UP(D)	1,446479	0,000513647
C1GGW1	nuclear cap-binding protein subunit 1	UP (8W)	1,419163	0,006836697
A0A0A0HTY8	nucleolar protein 58	UP(D)	1,401489	0,00064283
C1G015	pre-mRNA-splicing factor RBM22/SLT11	UP (8W)	1,400251	0,012238638
C1GKR7	large subunit ribosomal protein L24e	UP(D)	1,383279	0,000160866
C1G186	translation initiation factor 3 subunit E	UP (8W)	1,370763	0,021880757
C1GFI8	translation initiation factor 2A	UP (8W)	1,361679	0,003306043
C1FYJ6	histone H4	UP (12W)	1,35582	0,016446335
C1GAW6	large subunit ribosomal protein L34e	UP(D)	1,349162	0,000117482
C1GA20	large subunit ribosomal protein L11e	UP(D)	1,345788	0,000108069
C1G619	ribosome assembly protein SQT1	UP (12W)	1,343881	0,012496752
C1G5J0	small subunit ribosomal protein S15e	UP (8W)	1,335105	0,042150048
C1G016	complex III assembly factor LYRM7	UP (8W)	1,330954	0,01509806
C1G5N0	casein kinase II subunit alpha [EC:2.7.11.1]	UP(D)	1,31369	0,000931279
A0A0A0HVT2	26S proteasome regulatory subunit T3	UP(D)	1,298684	1,17191E-05
C1G0P0	molecular chaperone DnaK	UP (8W)	1,293241	0,005759296
C1GLJ7	small subunit ribosomal protein S26e	UP (12W)	1,281673	0,045542537
C1G945	large subunit ribosomal protein L30e	UP (12W)	1,277072	0,017924577
C1G7C1	26S proteasome regulatory subunit N2	UP(D)	1,274031	3,03631E-05
C1FZ00	large subunit ribosomal protein L37Ae	UP(D)	1,270958	0,000588591
C1GAT3	splicing factor U2AF 35 kDa subunit	UP(D)	1,270958	0,000514068
C1GBC4	mannosyl-oligosaccharide alpha-1,2-mannosidase [EC:3.2.1.113]	UP(D)	1,267656	8,26625E-05
C1G5P0	pre-mRNA-splicing helicase BRR2 [EC:3.6.4.13]	UP(D)	1,267304	0,000134116
C1GAF6	mitochondrial FAD-linked sulfhydryl oxidase [EC:1.8.3.2]	UP (12W)	1,255138	0,048940396
C1FYE5	glutamyl-tRNA synthetase [EC:6.1.1.17]	UP (8W)	1,249736	0,027076205
C1G1M5	ATP-dependent Clp protease ATP-binding subunit ClpB	UP(D)	1,231603	1,34334E-05
C1G561	aminoacyl tRNA synthase complex-interacting multifunctional protein 1	UP (8W)	1,22154	0,01136855

C1GIL9	arginyl-tRNA synthetase [EC:6.1.1.19]	UP (8W)	1,216406	0,005965772
C1G4C0	N-alpha-acetyltransferase 15/16, NatA auxiliary subunit	UP(D)	1,213493	7,68103E-05
A0A0A0HVH2	large subunit ribosomal protein L31e	UP (8W)	1,196067	0,003306043
C1GA10	ATP-dependent RNA helicase DDX3X [EC:3.6.4.13]	UP(D)	1,170383	0,000222134
C1FZL1	26S proteasome regulatory subunit T1	UP(D)	1,168201	0,000192931
C1G686	translation initiation factor 6	UP (12W)	1,168028	0,038574499
C1GEJ7	transitional endoplasmic reticulum ATPase	UP(D)	1,162316	1,34334E-05
C1GL26	translation initiation factor 3 subunit B	UP(D)	1,162189	0,000117426
C1GL49	replication factor A1	UP(D)	1,153131	0,000301604
C1GLD7	dolichyl-phosphate-mannose-protein mannosyltransferase [EC:2.4.1.109]	UP (12W)	1,146952	0,010907089
C1GJ94	tRNA threonylcarbamoyladenosine dehydratase	UP (12W)	1,143352	0,015401213
C1GK43	C2H2-type domain-containing protein	UP (12W)	1,139825	0,035718382
A0A0A0HVP9	small subunit ribosomal protein S30e	UP(D)	1,13966	0,000251289
C1GDG9	Mitochondrial inner membrane protease ATP23	UP (8W)	1,131916	0,033611475
C1G390	phenylalanyl-tRNA synthetase beta chain [EC:6.1.1.20]	UP (8W)	1,130716	0,025085175
C1GF47	large subunit ribosomal protein L23Ae	UP (8W)	1,130133	0,014815223
A0A0A0HVN3	ATP-dependent RNA helicase DDX5/DBP2 [EC:3.6.4.13]	UP(D)	1,124478	2,4505E-05
C1FZ06	NA	UP(D)	1,107628	1,34334E-05
C1GJT7	mannosyl-oligosaccharide alpha-1,3-glucosidase [EC:3.2.1.207]	UP(D)	1,10585	0,00023807
C1GAG5	translation initiation factor 3 subunit I	UP (12W)	1,099492	0,044193099
C1G8V3	tyrosyl-tRNA synthetase [EC:6.1.1.1]	UP(D)	1,099311	0,000811179
C1G6F6	heat shock 70kDa protein 1/2/6/8	UP (8W)	1,098813	0,005276659
C1GJI0	Probable endonuclease LCL3	UP (8W)	1,095519	0,035360636
C1G373	translation initiation factor 3 subunit C	UP (8W)	1,094932	0,006800777
C1FZ84	Translocon-associated protein	UP (12W)	1,08839	0,011398206
C1G3P9	calnexin	UP(D)	1,087582	1,93952E-05
C1GBG1	aspartyl-tRNA synthetase [EC:6.1.1.12]	UP (8W)	1,086092	0,028162637
C1G5M9	valyl-tRNA synthetase [EC:6.1.1.9]	UP(D)	1,082316	4,61283E-05
C1G7F3	Piwi domain-containing protein	UP (12W)	1,081471	0,016510517
C1G159	26S proteasome regulatory subunit T5	UP (8W)	1,075184	0,016060643
C1G631	26S proteasome regulatory subunit T2	UP(D)	1,074279	0,000437759
C1GEN5	large subunit ribosomal protein L4e	UP(D)	1,071924	3,04379E-05
C1GAE3	mitochondrial transcription factor 1	UP(D)	1,071131	5,60622E-05

C1FYW9;C1GLA5	Ubiquitin-60S ribosomal protein L40	UP (8W)	1,06592	0,02857375
C1G570	prolyl-tRNA synthetase [EC:6.1.1.15]	UP(D)	1,06561	7,68103E-05
C1G6V3	Ubiquitin-like domain-containing protein	UP (8W)	1,062992	0,035329695
C1GAQ4	DNA-directed RNA polymerase II subunit RPB2 [EC:2.7.7.6]	UP(D)	1,062347	0,000242791
C1GLX8	chaperonin GroEL	UP (8W)	1,056111	0,00766075
C1GG76	small subunit ribosomal protein S18e	UP(D)	1,054132	2,71675E-05
C1G2H7	MYG1 protein	UP (8W)	1,053521	0,006800777
C1GCN9	mannosyl-oligosaccharide glucosidase [EC:3.2.1.106]	UP(D)	1,052265	0,000955992
C1GF61	seryl-tRNA synthetase [EC:6.1.1.11]	UP(D)	1,049551	0,000159309
C1G4I3	obg-like ATPase 1	UP (8W)	1,047117	0,003306043
C1GG12	glutaminyl-tRNA synthetase [EC:6.1.1.18]	UP(D)	1,042749	3,96715E-05
C1GF71	histone H2B	UP (12W)	1,041082	0,018150971
C1GB62	leucyl-tRNA synthetase [EC:6.1.1.4]	UP (12W)	1,037888	0,034503547
C1GL98	polyadenylate-binding protein	UP (8W)	1,033177	0,01466991
C1GD28	protein transport protein SEC13	UP (12W)	1,027963	0,010753681
C1GLI9	elongation factor 2	UP(D)	1,027267	2,03704E-05
C1G0I9	Glucosidase 2 subunit beta	UP (8W)	1,026761	0,035158003
C1GCZ3	UDP-glucose:glycoprotein glucosyltransferase [EC:2.4.1.-]	UP (8W)	1,026419	0,044426383
C1GMJ8	T-complex protein 1 subunit epsilon	UP(D)	1,022092	9,19141E-05
C1G5V0	Nucleic acid-binding protein	UP (8W)	1,019347	0,030843909
C1GG28	elongation factor 1-gamma	UP(D)	1,018335	7,5032E-05
A0A0A0HUI8	Histone H2A.Z	UP (12W)	1,013555	0,032627654
C1FZI7	Vacuolar protein sorting/targeting protein 10	UP (12W)	1,010704	0,040518217
C1GL41	T-complex protein 1 subunit beta	UP (8W)	1,010658	0,039335286
C1G4I4	Translation machinery-associated protein 20	UP (12W)	1,007096	0,048940396
C1FYD3	UBC core domain-containing protein	UP(D)	1,006869	0,000587855
C1GA62	alpha-mannosidase [EC:3.2.1.24]	UP(D)	1,000186	5,48833E-05
C1G8H6	endoplasmic reticulum chaperone BiP [EC:3.6.4.10]	UP(D)	0,998596	0,000423833
C1FYK2	isoleucyl-tRNA synthetase [EC:6.1.1.5]	UP(D)	0,997139	0,000437759
C1G6R0	protein SSD1	UP (12W)	0,991494	0,034781743
C1GJL2	cysteinyl-tRNA synthetase [EC:6.1.1.16]	UP(D)	0,988661	0,000462567
C1G8B2	syntaxin 8	UP (12W)	0,984268	0,038765677
C1GB82	protein transport protein SEC24	UP(D)	0,980118	0,00066464
C1G9Z7	translation initiation factor 2 subunit 3	UP (12W)	0,979993	0,032572703
C1G6R9	protein mago nashi	UP (8W)	0,979824	0,034729177
C1GDY2	U2 small nuclear ribonucleoprotein A'	UP (8W)	0,970768	0,034729177

C1G349	protein disulfide-isomerase [EC:5.3.4.1]	UP (12W)	0,961615	0,01806641
C1GI40	serine incorporator 1	UP (12W)	0,959496	0,026955327
C1GLI2	heat shock 70kDa protein 1/2/6/8	UP(D)	0,959262	0,000118
C1GJ52	phospholipase A-2-activating protein	UP (8W)	0,954214	0,01254854
C1GMI6	lysyl-tRNA synthetase, class II [EC:6.1.1.6]	UP(D)	0,952846	0,000534774
C1G4T3	elongation factor Tu	UP (12W)	0,947064	0,010753681
C1G6I0	heat shock 70kDa protein 4	UP(D)	0,943117	0,000143959
C1G7B9	transcription elongation factor SPT5	UP(D)	0,929019	0,000293052
C1FYQ2	T-complex protein 1 subunit gamma	UP(D)	0,924727	3,69337E-05
C1G1H8	histone chaperone ASF1	UP(D)	0,915864	0,000823078
C1FZL0	T-complex protein 1 subunit delta	UP(D)	0,904342	1,62924E-05
C1FZG2	alanyl-tRNA synthetase [EC:6.1.1.7]	UP(D)	0,903844	0,000882487
C1G554	ubiquitin-activating enzyme E1 [EC:6.2.1.45]	UP (8W)	0,902624	0,030843909
C1G9A3	Uracil-DNA glycosylase	UP(D)	0,901583	0,000252883
C1G3P2	histidyl-tRNA synthetase [EC:6.1.1.21]	UP(D)	0,895699	0,001465654
C1G571	T-complex protein 1 subunit alpha	UP(D)	0,893508	0,000175009
C1G6U0	ATP-dependent Clp protease ATP-binding subunit ClpB	UP (8W)	0,891804	0,025121662
C1G8E5	peptide chain release factor subunit 3	UP(D)	0,885301	0,000709328
C1GJZ7	small subunit ribosomal protein S21e	UP (8W)	0,883043	0,01634925
A0A0A0HUX3	Proliferating cell nuclear antigen	UP (8W)	0,880445	0,04645427
C1G391	small subunit ribosomal protein S3e	UP(D)	0,870084	3,07307E-05
C1GFY2	ADP-ribose 1''-phosphate phosphatase	UP (8W)	0,867321	0,019249059
C1G514	Hsp90 chaperone protein kinase-targeting subunit	UP (8W)	0,858898	0,043289385
C1GLJ3	T-complex protein 1 subunit eta	UP(D)	0,857928	0,000330967
C1GF57	nucleosome assembly protein 1-like 1	UP (12W)	0,856219	0,038574499
C1G632	ubiquitin-conjugating enzyme (huntingtin interacting protein 2) [EC:2.3.2.23]	UP (12W)	0,855249	0,038393014
C1GKC9	molecular chaperone HtpG	UP(D)	0,84908	0,000395866
C1G1B6	Non-histone chromosomal protein 6	UP (8W)	0,844169	0,030843909
C1G9T0	translation initiation factor 3 subunit A	UP(D)	0,843625	8,26625E-05
C1G741	LsmAD domain-containing protein	UP (12W)	0,818457	0,04712458
C1GLA9	translation initiation factor 5B	UP(D)	0,798049	0,000175009
C1GGR5	small subunit ribosomal protein S20e	UP (8W)	0,793211	0,018011162
C1GF12	glycyl-tRNA synthetase [EC:6.1.1.14]	UP(D)	0,788962	0,000297057
A0A0A0HYV6	large subunit ribosomal protein L5e	UP (8W)	0,78714	0,033696224
C1GC92	prohibitin 1	UP (12W)	0,771972	0,021736563
C1G9A5	protein disulfide-isomerase A1 [EC:5.3.4.1]	UP(D)	0,743746	0,000242791
C1GGQ1	peptidyl-prolyl isomerase D [EC:5.2.1.8]	UP (8W)	0,733369	0,027076205

C1GKM9	protein disulfide-isomerase A6 [EC:5.3.4.1]	UP(D)	0,715722	0,000379134
C1G6W9	protein LSM14	UP (12W)	0,672222	0,048940396
**Transport**				
C1GGG3	Phosphate transporter	UP (12W)	2,442638	0,044193099
C1G0S7	EF-hand domain-containing protein	UP (12W)	1,665559	0,010753681
C1GAX4	ATP-binding cassette, subfamily B, vacuolar membrane transporter HMT1/ACLQ	UP (12W)	1,635563	0,043449634
C1G5L4	dynein cytoplasmic 1 intermediate chain	UP (12W)	1,458331	0,021736563
C1GF89	Mitochondrial thiamine pyrophosphate carrier 1	UP (8W)	1,45395	0,005815857
C1FYM2	vesicle-fusing ATPase [EC:3.6.4.6]	UP(D)	1,245729	8,26625E-05
C1GE52	actin-related protein 3	UP(D)	1,237219	0,001837096
C1G6Y4	carnitine O-acetyltransferase [EC:2.3.1.7]	UP (12W)	1,189555	0,010753681
C1GHE5	importin subunit beta-1	UP(D)	1,187745	0,000620203
C1G819	solute carrier family 25, member 33/36	UP (8W)	1,13966	0,028162637
C1GLV2	solute carrier family 25 (peroxisomal adenine nucleotide transporter), member 17	UP (8W)	1,041889	0,018887049
C1GGI3	Golgi transport protein Sly1	UP (8W)	1,018161	0,046317235
C1G3T6	P-type Na+/K+ transporter [EC:7.2.2.3 7.2.2.-]	UP (12W)	1,016642	0,025234124
C1G635	ATP-binding cassette, subfamily F, member 2	UP(D)	0,990747	0,000529237
C1G750	Dynein light chain	UP (12W)	0,983748	0,018150971
C1GEX3	ATP-binding cassette, subfamily C (CFTR/MRP), member 1 [EC:7.6.2.3]	UP(D)	0,977466	0,000651657
A0A0A0HW80	vacuolar iron transporter family protein	UP(D)	0,970404	0,000224532
C1G8G5	capping protein (actin filament) muscle Z-line, alpha	UP(D)	0,969929	0,001813377
C1GID7	carnitine O-acetyltransferase [EC:2.3.1.7]	UP(D)	0,952259	0,000175009
C1G6X9	High-affinity methionine permease	UP (8W)	0,940089	0,027543958
C1G1D8	oxysterol-binding protein-related protein 9/10/11	UP(D)	0,936698	0,001839063
C1GBG4	coatomer subunit gamma	UP(D)	0,935058	8,00793E-05
C1FZH6	dynamin 1-like protein [EC:3.6.5.5]	UP (8W)	0,910345	0,020249279
C1GLM2	voltage-dependent anion channel protein 2	UP (12W)	0,907959	0,03059419
A0A0A0HWS3	vacuolar protein sorting-associated protein 1	UP(D)	0,903541	0,000203639
A0A0A0HT88	Long-chain fatty acid transporter	UP (8W)	0,868167	0,018215419
C1GIB2	protein transport protein SEC31	UP(D)	0,865188	0,000330967
C1GA14	clathrin heavy chain	UP (12W)	0,859461	0,034014219

C1FZ88	importin subunit alpha-6/7	UP(D)	0,838203	0,000322749
C1G9E6	mitochondrial import inner membrane translocase subunit TIM10	UP(D)	0,831546	0,001599547
C1G8G0	importin-4	UP (12W)	0,830463	0,035118646
C1G4B9	mitochondrial import inner membrane translocase subunit TIM54	UP (8W)	0,821235	0,037862655
C1GAF5	coatomer subunit alpha	UP(D)	0,787953	0,001225385
A0A0A0HWM 5	mitochondrial import receptor subunit TOM70	UP(D)	0,763927	0,002096976
C1G6U8	P-type Ca2+ transporter type 2C [EC:7.2.2.10]	UP (12W)	0,763312	0,035368073
C1G3C4	solute carrier family 25 (mitochondrial adenine nucleotide translocator), member 4/5/6/31	UP(D)	0,740206	0,000267021
C1GB16	mitochondrial import inner membrane translocase subunit TIM50	UP (12W)	0,721871	0,042111956
**Other metabolisms**				
A0A0A0HQV2	NAD(P)H-hydrate epimerase [EC:5.1.99.6]	UP (8W)	3,062277	0,00918935
A0A0A0HWR 3	phosphoribosylformylglycinamidine synthase [EC:6.3.5.3]	UP (8W)	1,833492	0,018011162
C1GMH1	biotin synthase [EC:2.8.1.6]	UP(D)	1,751156	0,000224532
C1GAV1	Nicotinamidase	UP (12W)	1,741082	0,037489965
C1G168	succinate---hydroxymethylglutarate CoA-transferase [EC:2.8.3.13]	UP(D)	1,573543	0,000385662
A0A0A0HR31	phosphoribosylaminoimidazole carboxylase [EC:4.1.1.21]	UP (12W)	1,465811	0,034368589
C1G0E1	urate oxidase [EC:1.7.3.3]	UP (8W)	1,446694	0,016560411
C1GCJ6	2-methylcitrate dehydratase [EC:4.2.1.79]	UP (8W)	1,442844	0,013650279
C1GCX5	glycine hydroxymethyltransferase [EC:2.1.2.1]	UP(D)	1,436739	2,03704E-05
C1FYK8	cyclin-dependent kinase [EC:2.7.11.22]	UP (8W)	1,409371	0,014049764
C1GFX8	manganese-transporting P-type ATPase [EC:7.2.2.-]	UP (12W)	1,395128	0,009555529
C1G7B2	glutaminyl-peptide cyclotransferase [EC:2.3.2.5]	UP (8W)	1,385701	0,02857375
C1G5I8	carbamoyl-phosphate synthase large subunit [EC:6.3.5.5]	UP (8W)	1,374854	0,011719078
C1GLI4	glucosamine-phosphate N-acetyltransferase [EC:2.3.1.4]	UP (8W)	1,368562	0,02090407
C1GBS0	carbamoyl-phosphate synthase / aspartate carbamoyltransferase [EC:6.3.5.5 2.1.3.2]	UP(D)	1,331486	0,000835911
C1G7D5	Pantetheine-phosphate adenylyltransferase	UP (12W)	1,312928	0,045542537
C1GDH9	extracellular matrix protein 14 [EC:3.4.17.-]	UP (8W)	1,321069	0,0056646
C1GKQ4	chitin synthase [EC:2.4.1.16]	UP (12W)	1,144736	0,021736563

C1G9D5	urease [EC:3.5.1.5]	UP (8W)	1,312536	0,011082888
C1GB78	uroporphyrinogen-III synthase [EC:4.2.1.75]	UP (8W)	1,31213	0,008729208
C1G5Z2	acetyl-CoA hydrolase [EC:3.1.2.1]	UP (8W)	1,283635	0,028211518
C1GBF1	AFG3 family protein [EC:3.4.24.-]	UP (8W)	1,243799	0,006412745
C1GEY6	pyridoxal 5'-phosphate synthase pdxS subunit [EC:4.3.3.6]	UP (8W)	1,240117	0,00687719
C1FYF6	adenylosuccinate synthase [EC:6.3.4.4]	UP (8W)	1,222841	0,014400463
C1FYZ8	Epoxide hydrolase	UP (12W)	1,221769	0,041170587
C1G5S1	Glutathione S-transferase kappa	UP (8W)	1,187116	0,037383913
A0A0A0HSU0	insulysin [EC:3.4.24.56]	UP (12W)	1,166518	0,043130713
C1G0X3	2-succinylbenzoate-CoA ligase	UP(D)	1,16117	0,000107991
C1G0D4	catalase [EC:1.11.1.6]	UP(D)	1,14811	0,000692846
C1G7R6	metallopeptidase MepB [EC:3.4.24.-]	UP (8W)	1,141494	0,005516536
C1G0S4	biotin---protein ligase [EC:6.3.4.9 6.3.4.10 6.3.4.11 6.3.4.15]	UP(D)	1,126305	0,000889849
C1GCZ0	Allantoinase	UP (8W)	1,115062	0,04023474
C1GAY4	peptide-methionine (S)-S-oxide reductase [EC:1.8.4.11]	UP (8W)	1,094903	0,005276659
C1GEY4	Xaa-Pro aminopeptidase [EC:3.4.11.9]	UP (8W)	1,08198	0,007353391
A0A0A0HTY3	1,4-alpha-glucan branching enzyme [EC:2.4.1.18]	UP (8W)	1,064572	0,008670622
C1GA13	phosphoribosylaminoimidazolecarboxamide formyltransferase / IMP cyclohydrolase [EC:2.1.2.3 3.5.4.10]	UP(D)	1,055029	0,00054087
C1GA17	pyruvate carboxylase [EC:6.4.1.1]	UP(D)	1,021473	4,70896E-05
C1G167	riboflavin synthase [EC:2.5.1.9]	UP (8W)	1,005041	0,013621638
C1GIX8	precorrin-2 dehydrogenase / sirohydrochlorin ferrochelatase [EC:1.3.1.76 4.99.1.4]	UP(D)	0,992198	0,000159309
C1GLG2	adenylate kinase [EC:2.7.4.3]	UP(D)	0,985231	0,000811179
C1GHS5	Amidase	UP (12W)	0,980662	0,021736563
C1GAT8	UTP--glucose-1-phosphate uridylyltransferase [EC:2.7.7.9]	UP(D)	0,978168	0,000108069
C1GB27	adenylosuccinate lyase [EC:4.3.2.2]	UP(D)	0,977705	0,000308052
C1G7J4	aminopeptidase 2 [EC:3.4.11.-]	UP(D)	0,968431	0,000127385
C1G7L5	arylesterase / paraoxonase [EC:3.1.1.2 3.1.8.1]	UP(D)	0,955125	0,000231128
C1GD24	dipeptidyl-peptidase III [EC:3.4.14.4]	UP(D)	0,954726	0,00107244
C1G164	phosphoacetylglucosamine mutase [EC:5.4.2.3]	UP (8W)	0,90123	0,034875515
C1GBQ7	chorismate mutase [EC:5.4.99.5]	UP (12W)	0,90117	0,025552362
C1GD57	Xaa-Pro dipeptidase [EC:3.4.13.9]	UP(D)	0,894158	0,000352749
C1GGV9	Aminopeptidase	UP(D)	0,870813	0,000268322
C1G9D6	cytochrome-b5 reductase [EC:1.6.2.2]	UP (8W)	0,84809	0,031954553
C1G713	H+-translocating NAD(P) transhydrogenase [EC:1.6.1.2 7.1.1.1]	UP (12W)	0,840913	0,01806641

C1GML7	phosphoenolpyruvate carboxykinase (ATP) [EC:4.1.1.49]	UP (8W)	0,831839	0,01155815
C1G9K7	phosphomannomutase [EC:5.4.2.8]	UP (8W)	0,822025	0,02090407
C1FYN6	UDP-galactopyranose mutase	UP (12W)	0,799344	0,021736563
C1G8F8	Ser/Thr protein phosphatase	UP (12W)	0,75944	0,044193099
C1G215	Phosphoglycolate phosphatase	UP (8W)	0,686611	0,035573455
C1G7T4	ATP-dependent Lon protease [EC:3.4.21.53]	UP (12W)	0,659998	0,041170587
C1GIJ3	Murein transglycosylase	DOWN (8W)	-1,77496	0,044788211
**Cell cycle**				
C1GA76	structural maintenance of chromosome 1	UP(D)	1,901341	0,000243877
C1GL30	Velvet domain-containing protein	UP (12W)	1,379177	0,021989098
C1GJ83	cell division control protein 11	UP(D)	1,172387	4,61283E-05
C1GII4	septin 7	UP(D)	1,141771	0,00018346
C1G389	serine/threonine-protein phosphatase 2A regulatory subunit A	UP (12W)	1,128973	0,013179723
C1GEM8	cell division control protein 12	UP(D)	1,115969	3,69337E-05
C1G994	ANAPC4_WD40 domain-containing protein	UP(D)	1,095215	3,96715E-05
C1GCT8	GTP-binding nuclear protein Ran	UP(D)	0,985808	7,68103E-05
**Signal transduction**				
C1GDE6	GTPase KRas	UP(D)	1,464659	0,000330967
C1GF62	Rheb small monomeric GTPase RhbA	UP (12W)	1,330107	0,044193099
C1GKC8	Ras GTPase-activating protein-binding protein 2	UP (8W)	1,079013	0,034445052
C1FZF8	Rho-GTPase-activating protein	UP (12W)	1,06306	0,029656674
C1GLV1	Ras-related protein Rab-1A	UP(D)	0,944363	0,000190119
C1G2S7	Rab GDP dissociation inhibitor	UP (8W)	0,915304	0,02857375
C1GB04	14-3-3 protein epsilon	UP(D)	0,89245	0,000108499
C1GEA4	AGC/PKC protein kinase	UP (12W)	0,808043	0,021989098
**Cytosketelon**				
C1GM22;C1G3F2	Tubulin alpha chain	UP (12W)	1,610509	0,01806641
C1FZT8	tubulin alpha	UP (12W)	1,011304	0,018150971
C1GFT4	Cytoskeleton assembly control protein	UP(D)	0,928838	0,000192526
C1GJ13	coronin-1B/1C/6	UP (12W)	0,811961	0,018150971
C1GDK5	Fimbrin	UP(D)	0,765673	0,00029774
**No annotation found**				
C1G2U9	NA	UP (8W)	2,212297	0,011412341
C1GIP9	NA	UP (12W)	2,116714	0,028013083
C1G7I0	NA	UP(D)	1,78265	0,000133721
C1G6F3	NA	UP (8W)	1,769059	0,015915364
C1GAT1	Amidase	UP (12W)	1,634443	0,007951887
C1G422	NA	UP (8W)	1,597073	0,02857375
C1G379		UP (8W)	1,579459	0,041534769

C1G5X0	Carrier domain-containing protein	UP(D)	1,512006	0,000258272
C1G2F0	NA	UP (12W)	1,51143	0,045189178
C1GA00	NA	UP (8W)	1,490337	0,008977012
C1G0C9	NA	UP (12W)	1,484294	0,021989098
C1G6Q3	NA	UP (8W)	1,340086	0,024125416
C1GFT1	NA	UP (12W)	1,228773	0,043449634
C1G4G5	uncharacterized protein	UP (12W)	1,176648	0,03059419
C1GBH7	NA	UP (12W)	1,142773	0,03646764
C1GK41	NA	UP(D)	1,11431	0,000567838
C1GJH6	NA	UP (8W)	1,061917	0,018238692
C1G101	NA	UP (8W)	1,061851	0,022464047
C1FYH3	NA	UP(D)	1,000493	0,000711383
C1GHL9	NA	UP(D)	0,958979	0,001050039
C1GJH8	NA	UP (8W)	0,933274	0,040972086
C1GMJ4	RING-type domain-containing protein	UP (12W)	0,911585	0,039029295
C1GBD3	NA	UP(D)	0,840433	0,000190119
C1GES0	apoptosis-inducing factor 2	UP (12W)	0,781637	0,021736563
C1G8P5	NA	UP (12W)	0,778281	0,034503547
C1G647	NA	UP (12W)	0,761338	0,041170587
C1GFJ7	NA	UP (12W)	0,748749	0,021736563
C1G3V2	NA	UP (12W)	0,747881	0,048940396
C1GAY7	NA	UP (8W)	0,727696	0,032954605
